# Multiple Primary Melanoma: A Five-Year Prospective Single-Center Follow-Up Study of Two MC1R R/R Genotype Carriers

**DOI:** 10.3390/life13102102

**Published:** 2023-10-23

**Authors:** Ana Maria Fagundes Sortino, Bianca Costa Soares de Sá, Marcos Alberto Martins, Eduardo Bertolli, Rafaela Brito de Paula, Clovis Antônio Lopes Pinto, Waldec Jorge David Filho, Juliana Casagrande Tavoloni Braga, João Pedreira Duprat Neto, Dirce Maria Carraro, Maria Paula Curado

**Affiliations:** 1 Clínica Dermatológica Dermatis, Rua Joaquim Floriano 466, Itaim Bibi, São Paulo 04534-002, SP, Brazil; 2 Hospital Sírio-Libanês, Rua Dona Adma Jafet 115, Bela Vista, São Paulo 01308-050, SP, Brazil; 3 AC Camargo Cancer Center, Rua Pires da Mota 1.167, Aclimação, São Paulo 01529-001, SP, Brazil; 4 Centro Universitário Saúde ABC, Surgery Department, Avenida Lauro Gomes 2000, Vila Sacadura Cabral, Santo André 09060-870, SP, Brazil; 5 A Beneficência Portuguesa de São Paulo–BP Mirante, Rua Martiniano de Carvalho 965, Bela Vista, São Paulo 01323-001, SP, Brazil; 6 Hospital Alemão Oswaldo-Cruz, Rua Treze de Maio 1815, Bela Vista, São Paulo 01323-903, SP, Brazil; waldecjorge@uol.com.br

**Keywords:** dysplastic nevus syndrome, skin cancer, melanoma, multiple primary melanoma, nevus associated melanoma, second primary melanoma, synchronous primary cutaneous melanomas, MC1R, total body skin photograph, digital dermoscopy, sequential digital dermoscopy imaging, reflectance confocal microscopy

## Abstract

**Simple Summary:**

This 5-year prospective single-center follow-up study of multiple primary melanomas in two first-degree relatives with *MC1R R/R* genotype is an eye opener for the need of strict melanocytic lesions monitoring in high-risk patients, combining clinical expertise and different, yet synergic, skin imaging technologies. Our cohort of 60 cutaneous melanomas present in only two individuals show a high percentage (83.3%). Second primary melanomas, alas 16.7% of our cases were invasive melanomas with Breslow’s thickness up to 1.5 mm. This proportion of potentially aggressive melanomas cannot be disregarded. We bring awareness to all medical specialties, government health managers, and health care insurances for the need of rigorous monitoring of high-risk patients and their families, allowing better chances of earlier cutaneous melanoma diagnosis and longer survivals.

**Abstract:**

Background: Multiple primary melanoma (MPM) is a diagnostic challenge even with ancillary imaging technologies available to dermatologists. In selected patients’ phenotypes, the use of imaging approaches can help better understand lesion characteristics, and aid in early diagnosis and management. Methods: Under a 5-year prospective single-center follow-up, 58 s primary melanomas (SPMs) were diagnosed in two first-degree relatives, with fair skin color, red hair, green eyes, and personal history of one previous melanoma each. Patients’ behavior and descriptive demographic data were collected from medical records. The information on the first two primary melanomas (PMs) were retrieved from pathology reports. The characteristics of 60 melanomas were collected from medical records, video dermoscopy software, and pathology reports. Reflectance confocal microscopy (RCM) was performed prior to excision of 22 randomly selected melanomas. Results: From February 2018 to May 2023, two patients underwent a pooled total of 214 excisional biopsies of suspect lesions, resulting in a combined benign versus malignant treatment ratio (NNT) of 2.0:1.0. The number of moles excised for each melanoma diagnosed (NNE) was 1.7:1.0 and 6.9:1.0 for the female and male patient respectively. The in-situ melanoma/invasive melanoma ratio (IIR) demonstrated a higher proportion of in-situ melanomas for both patients. From June 2018 to May 2023, a total of 58 SPMs were detected by the combination of total body skin exam (TBSE), total body skin photography (TBSP), digital dermoscopy (DD), and sequential digital dermoscopy imaging (SDDI) via comparative approach. The younger patient had her PM one month prior to the second and third cutaneous melanomas (CMs), characterizing a case of synchronous primary CM. The male older relative had a total of 7 nonsynchronous melanomas. Conclusions: This CM cohort is composed of 83.3% in-situ melanoma and 16.7% invasive melanoma. Both patients had a higher percentage of SPM with clinical nevus-like morphology (84.5%), global dermoscopic pattern of asymmetric multiple component (60.3%) and located on the lower limbs (46.6%). When RCM was performed prior to excision, 81% of SPM had features suggestive of malignancy. As well, invasive melanomas were more frequent in the lower limbs (40%). In the multivariate model, for the two high-risk patients studied, the chance of a not associated with nevus (“de novo”) invasive SPM diagnosis is 25 times greater than the chance of a diagnosis of a nevus-associated invasive SPM.

## 1. Introduction

A higher risk of melanoma development is thoroughly described in literature for patients with fair skin phenotype, red hair, green and blue eyes, high count of melanotic nevi (>60 lesions) and five or more excised dysplastic nevi [1,2,3]. Acquired nevi may also serve as precursor lesion for nevus-associated melanoma (NAM), representing approximately 30% of overall melanoma cases [4,5]. Cutaneous melanoma (CM) is known to mainly occur “de novo”, not associated with a nevus, especially in older patients compared to NAM [5]. An even greater risk of a second primary melanoma (SPM) happens in the presence of personal and/or family history of melanoma, with identified high and/or intermediate penetrance genetic mutations [3,6,7,8,9].

Multiple primary melanoma (MPM) is known to occur in 0.2 to 8.6% of patients diagnosed with one previous melanoma [1,2,10]. In spite of the fact that literature shows a significant decrease in tumor thickness of SPM, an overall worse survival was described for individuals with MPM [1]. Likewise, it’s been reported that 26 to 40% of MPM patients have synchronous SPM [2,10,11]. Non-invasive histomorphological evaluation, together with genetic studies, improves melanoma risk identification and early diagnosis, for a patient-tailored management [12]. Strict surveillance of high-risk patients is indicated for avoidance of missing a fast-growing tumor and for prognosis improvement [6,8,13,14,15,16,17].

Total body mapping (TBM) includes photographic documentation of the entire body surface followed by digital dermoscopy (DD) of selected melanocytic lesions, aiming to compare their evolution over time and identify new lesions [18,19]. A combination of clinical total body skin exam (TBSE), total body skin photography (TBSP), and sequential digital dermoscopy imaging (SDDI), may ameliorate early-stage melanoma diagnosis (in-situ and thinner Breslow depth) [13,15,20,21,22]. The additional level for examination of a suspect melanocytic lesion is by reflectance confocal microscopy (RCM), an in vivo, noninvasive, diagnostic tool that increases sensitivity for malignancy, improving physician’s confidence, and resulting in a lower number of excised lesions [12,16,23,24,25,26,27,28,29].

Half of the Brazilian population has European ancestry and are considered white. Skin color distribution of patients with melanoma in Brazil was 91% in fair skin individuals [30]. In this study we aim to provide insight into multiple primary melanoma (MPM), according to the rule of 3 [31] of a Brazilian family (fathers’ sister, father and daughter), by doing a prospectively documented follow-up of two first-degree relatives (father and daughter), with skin phototype I, red hair, green eyes, multiple blistering sunburns in childhood/adolescence, and personal history of one previous primary melanoma (PM) each. They are under ongoing strict monitoring by TBSE, TBSP, SDDI and RCM for selected lesions. We seek to understand the impact of follow-up examinations of two high-risk patients on SPM diagnosis (clinical, dermoscopic and RCM), test for correlations within time of SDDI follow-up to suspect melanoma, and histological characteristics of diagnosed melanomas during the study period. We are now at a 5-year follow-up and have a cohort of 60 diagnosed melanomas to date.

## 2. Materials and Methods

### 2.1. Study Design and Inclusion/Exclusion Criteria

This is a prospective observational study of two high-risk related individuals. Due to the high number of nevi, both patients were referred to the dermatologist, suggesting enrollment in routine dermatological clinical examination and TBM. Since June 2018, they are followed by the same dermatologist (corresponding author), with long term experience in skin cancers (over 20 years), at a private dermatological skin cancer clinic in São Paulo, SP, Brazil. They are also followed by a plastic surgeon and a clinical oncologist (co-authors). Over the study period they were seen by two other dermatologists, both coauthors (one specialized in familial melanoma), and two surgical oncologists (co-authors). The consultations and surgeries happened in different institutions in São Paulo, SP, Brazil, according to the attending doctor affiliation.

Both patients were regularly monitored by clinical TBSE, including scalp and visible mucosal areas; non-polarized TBSP from June 2018 to September 2020 and polarized TBSP from October 2020 to May 2023; handheld dermoscopy with a 10-fold magnification of all lesions (DermLite^®^ LLC, Aliso Viejo, CA, USA), allowing lesion selection for DD; and SDDI, using a high-definition video camera. All digital dermoscopic images, collected from June 2018 to May 2023, were contact polarized dermoscopy (CPD) with a standard 20-fold magnification. Suspicious nevi had additional non-contact polarized dermoscopic (NCPD) and contact non-polarized dermoscopy (CNPD) images, sometimes with magnification of 30 to 40-fold. (FotoFinder^®^; TeachScreen Software GmbH, Bad Birnbach, Germany). Reflectance confocal microscopy (RCM) (Vivascope^®^ 1500 and Vivascope^®^ 3000, Caliber I.D. Inc., Rochester, NY, USA) was performed only for equivocal lesions, with suspicious dermoscopic structures or featureless, and read by the same dermatologist. The instruments and methods have been described elsewhere [28,32].

The initial number of recorded nevi for SDDI was 455 (female) and 172 (male). Patients were scheduled for follow-up in 1, 3 or 6 months, depending on the findings. TBSPs were taken mostly within a 6 to 12-month interval. Specific body areas and close-up photographs of highly suspicious moles were captured, the lesions gained new numbered DD positions, to facilitate their identification by the surgeon performing the excisional biopsies. That led to more than one position number for the same lesion. The final number of recorded positions were 689 and 313, for female and male respectively.

All data were encoded by lesion position number and used anonymously. Eligibility criteria were all melanomas diagnosed from February 2018 to May 2023. All excised nevi, keratinocytic lesions (benign and malignant), and vascular lesions were described and had their distribution calculated but were excluded from further statistical analysis.

Surgeries were performed mostly by the same plastic surgeon and a few by surgical oncologists. All excised lesions were sent for histological evaluations at A.C.Camargo Cancer Centre (ACCCC) pathology laboratory and read by at least two highly specialized pathologists (co-authors) with extensive experience in dermatopathology and melanocytic tumors, assuring accurate and complete pathologic reporting.

### 2.2. Data and Analysis

Demographic data of the two studied patients, such as gender, skin phototypes according to Fitzpatrick scale [33], age at first diagnosis, age at SPM excisions, anatomical location, laterality, and genetic mutation status, were extracted from clinical, photographic, and pathology/laboratory records.

We aimed to elucidate the time of SDDI follow-up (months between first and last digital dermoscopy prior to excision) and to learn the interval (months) between the last dermoscopy and the excisional biopsy. In regard to analytical and descriptive data of the 60 excised melanomas, we evaluated 58 SPMs clinically by age at excision, location (acral, head/neck, lower limbs, perineal, dorsal trunk, ventral trunk and upper limbs), laterality (left, midline and right), five morphological clusters identified as clinical pattern [34] (typical melanoma, nevus-like, amelanotic/nonmelanoma skin cancer-like, seborrheic keratosis-like and lentigo/lentigo maligna-like), clinical pigmentation (pigmented, hyperpigmented, hypopigmented, amelanotic/pink lesion), palpability (yes or no), and borders (well-defined, ill-defined or not defined). For the two PM missing/unknown data were excluded from statistical analysis.

Data was collected on global dermoscopic pattern, melanoma specific dermoscopic patterns, use and usefulness of RCM [35,36,37,38]. Six global dermoscopic pattern were selected for lesion evaluation (asymmetric multiple component, symmetric multiple component, asymmetric bicomponent, symmetric bicomponent, homogeneous and starburst). Up to six melanoma specific dermoscopic patterns per SPM diagnosed were identified and annotated from a total of 30 melanoma specific patterns from literature (atypical pigment network, patchy peripheral atypical pigment network, angulated lines, negative network, atypical streaks/pseudopods, atypical dots, atypical globules, tiered globules, tan structureless areas/hypopigmented structureless areas, prominent skin markings, multiple small hyperpigmented areas, atypical blotches/irregular hyperpigmented structureless areas, raised blue-white structureless area/blue-white vail, flat blue-white structureless area, granularity/peppering, regression scar-like depigmentation, shiny white structures, structureless pink, structureless brown, structureless blue, structureless blue black nodule, comma/curved vessels, dotted vessels, serpentine/small lines vessels, corkscrew vessels, polymorphous vessels, milky red areas, milky red globules, targetoid structures as globule or dotted vessel, and brown circles).

Lesions that were indeterminate (featureless under dermoscopy) or in body sites that the patient would’ve rather avoid surgery had RCM performed and registered (yes or no) and mentioned if RCM was suggestive of malignancy (yes or no). When malignancy was suspected the lesion was excised. For this study we only looked at RCM performed on confirmed 22 SPMs.

Pathology reports of 60 CM (2 PMs; 58 SPMs) were assessed to collect macroscopic lesion size in millimeters (mm), diagnosis and Breslow thickness (in-situ melanoma, invasive melanoma < 1 mm and invasive melanoma > 1 mm), type of melanoma (superficial spreading, lentiginous, nodular, acral lentiginous, amelanotic, nevoid, spitzoid, desmoplastic, lentigo maligna and lentigo maligna melanoma), associated with a preexisting nevus (yes or no) and type of nevi found in NAM (juncional nevus, compound nevus, dermal nevus and dysplastic nevus), histological regression (yes or no), intratumoral inflammatory infiltrate (yes or no), compromised margins after excisional biopsy (yes or no), wide local excision (yes or no), sentinel lymph node biopsy (SLNB) (yes or no). Our cohort has 50 in-situ and 10 invasive melanomas. Invasive SPM had a Breslow thickness range from 0.2 to 1.5 mm (nine cases of invasive melanoma < 1 mm and one invasive melanoma > 1 mm) and the number of mitoses per 10 microscopic field diameter was zero in eight cases and 1 in two cases.

Moreover, we checked the relationship between diagnosis (in-situ × invasive CM) and the studied variables: location, laterality, clinical pattern, clinical pigmentation, palpable lesion, borders, global dermoscopic pattern, RCM performed, RCM suggestive of malignancy, nevus associated, size (mm), histological regression, intratumoral inflammatory infiltrate, margins after biopsy, and wide local excision.

This study was conducted in accordance with the Declaration of Helsinki [39]. The images are anonymous and not identifiable, and patients signed written informed consent to be photographed and followed. A second patient consent for the use and publication of collected data and images was signed by both patients.

### 2.3. Statistical Analysis

We calculated ratios for the treatment of benign excised lesions (acquired nevi, SK, and hemangioma) for each skin cancer diagnosed (BCC, SCC, and AK): number-needed-to-treat (NNT); the number of excised benign nevi for each diagnosed melanoma (number needed to excise: NNE); and the number of lesions excised by patient request for each diagnosed melanoma (patient number needed to excise: PNNE). The proportion of in-situ versus invasive melanomas (in-situ/invasive ratio: IIR) was calculated to check if a smaller NNE was associated with a higher proportion of invasive melanomas.

Information on quantitative variables was summarized as mean, standard deviation, median, interquartile range, minimum and maximum values, and number of valid observations. Information on qualitative variables was summarized as simple frequency and percentage. To compare more than two independent groups in relation to qualitative variables, the chi-square test was considered or, when necessary, the likelihood ratio test. To compare more than two independent groups in relation to quantitative variables with Gaussian distribution, the analysis of variance model (ANOVA) was used followed by Bonferroni multiple comparisons or, in the case of variables without Gaussian distribution, the non-parametric Kruskal-Wallis test followed by non-parametric Mann-Whitney tests with Bonferroni correction. To compare two independent groups in relation to quantitative variables with Gaussian distribution, the parametric Student’s *t*-test was used or, in the case of variables without Gaussian distribution, the non-parametric Mann-Whitney test. To compare two quantitative variables with each other (correlation between two quantitative variables), Pearson’s correlation coefficient was used (when both variables had a Gaussian distribution) or Spearman’s correlation coefficient (when at least one of the variables did not have a Gaussian distribution). Missing data were excluded, and results were based on valid data. The significance level adopted is 5% (*p*-value ≤ 0.05).

To verify if there is a relationship between diagnosis (in-situ melanoma × invasive melanoma) and the variables: location, laterality, clinical pattern, clinical pigmentation, palpable lesion, borders, global dermoscopic pattern, RCM performed, RCM suggestive of malignancy, nevus associated, size (mm), histological regression, intratumoral inflammatory infiltrate, margins after biopsy, and wide local excision, the chi-square test was used or, when necessary, Fisher’s exact test or likelihood ratio. Significant variables at a maximum significance level of 10% (*p*-value ≤ 0.10) were selected for a multivariate logistic regression model. The significance level adopted is 5% (*p*-value ≤ 0.05).

To calculate the cumulative trend/risk of a new melanoma for male and female in relation to the number of melanomas, estimation curves were calculated to appraise the cumulative number of melanomas over time. Statistical analysis used IBM^®^ SPSS^®^ Statistics V.22 (IBM Corp., Armonk, NY, USA) and were performed in June 2023.

## 3. Results

**Figure 1:**
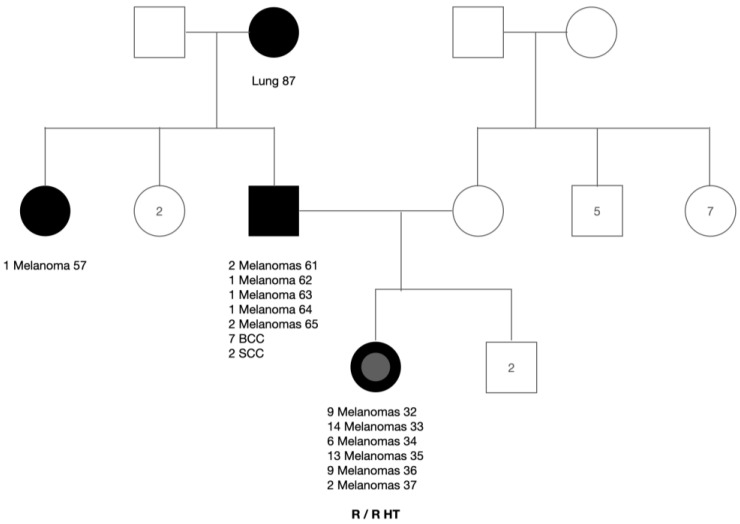
Heredogram of family studied.

In this prospective cohort, the family has three members with diagnosis of melanoma (Figure 1). Father’s sister, diagnosed in August 2011, with an upper limb invasive primary NAM, superficial spreading type, Breslow thickness of 1.4 mm, 2 mitoses per 10 microscopic field diameter, and intratumoral inflammatory infiltrate (not followed). Father with a previous diagnosis of lower limb invasive “de novo” PM (February 2018), along with more than 150 nevi. After the aunt and father’s diagnosis, the daughter requested the plastic surgeon for the excision of her upper limb in-situ nevus-associated PM (May 2018). The surgeon referred her to the dermatologist due to over 450 nevi for follow-up.

**Figure 2:**
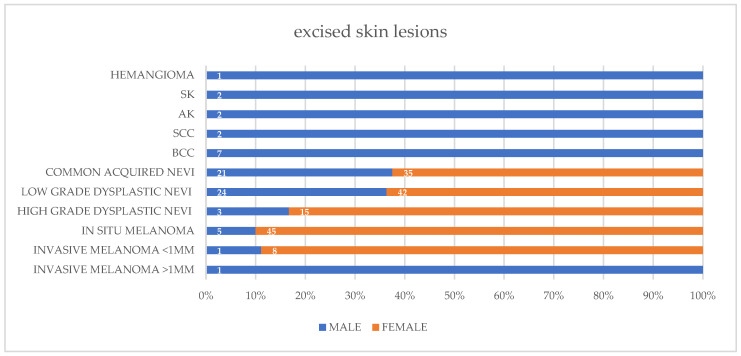
Proportion of histological diagnosis of 214 excised lesions, by patient gender, from February 2018 to May 2023.

From February 2018 to May 2023, both patients underwent excisions of suspect lesions. A total of 214 excisional biopsies were performed with 1–2 mm margins, resulting in 140 nevi, 60 melanomas, 7 basal cell carcinomas (BCC), 2 squamous cell carcinomas (SCC), 2 actinic keratosis (AK), 2 seborrheic keratosis (SK) and one hemangioma (Table S1). Regarding melanocytic nevi there were 56 common acquired nevi, 66 low-grade dysplastic nevi and 18 high-grade dysplastic nevi. The proportion of diagnosed lesions by patient gender is presented in Figure 2. The treatment ratio of benign excised lesions for each skin cancer diagnosed: number-needed-to-treat (NNT) was 1.7 female lesion and 2.8 male lesions excised for each skin cancer. Combining both genders the NNT was 2.0 benign lesions excised for each skin cancer. The ratio of acquired nevi excised for each cutaneous melanoma diagnosed: number-needed-to-excise (NNE) is a metric used to express the efficiency of diagnostic accuracy of CM [40]. In our cohort the NNE was 1.7 female nevus and 6.9 male nevi excised for each melanoma. Combining both genders the NNE was 2.3 acquired nevi excised for each CM (Table 1).

**Figure 3:**
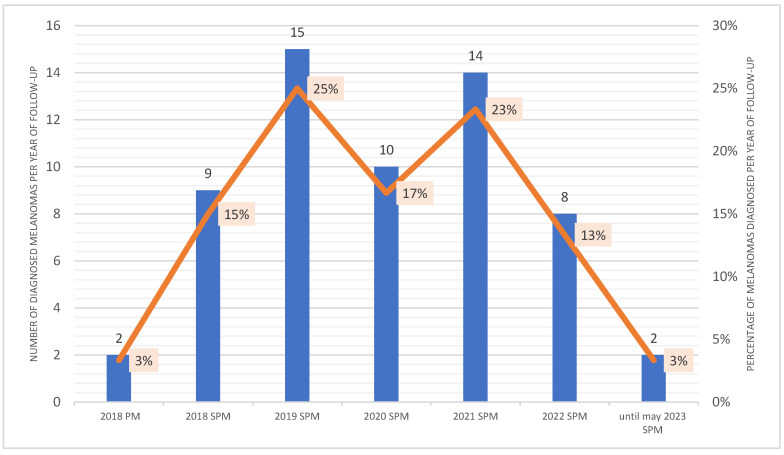
Proportion of melanomas diagnosed from February 2018 to May 2023.

**Figure 4:**
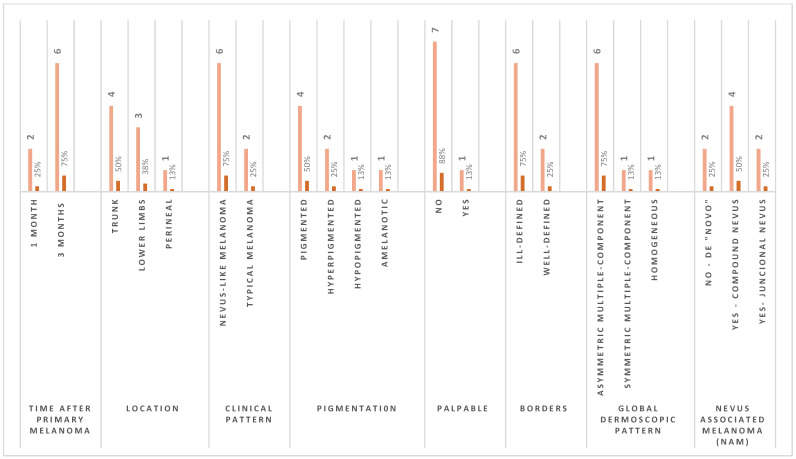
Total number (light orange bars) and percentages (dark orange bars) of 8 synchronous SPMs diagnosed on the female patient, at first and third month. Their location, clinical pattern, pigmentation, palpability, borders, global dermoscopic pattern and histological association with nevus are summarized above.

From June 2018 to May 2023, a total of 58 SPMs were detected by the combination of TBSE, TBSP, DD, and SDDI (using the comparative approach). Figure 3 exhibits the proportion of melanomas diagnosed during the intervention. Over the follow-up period, RCM was performed prior to excision of 22 (38%) lesions that were dermoscopically indeterminate or in body sensitive areas, of those 18 (81%) had RCM features suggestive of malignancy. Synchronous melanomas are lesions diagnosed within 3 months of the first diagnosis [2]. The female patient had her PM one month prior to the second and third synchronous CM, characterizing a case of synchronous primary cutaneous melanomas, and corresponding to 0.5% of CM patients [2]. A total of 8 synchronous in-situ SPMs were diagnosed during the initial 3-month period of her follow-up (Figure 4), probably one of the highest numbers of synchronous CM published to date. Figures S1a,b and S2 show both synchronous diagnosed at the first TBM.

### 3.1. Baseline Demographic Characteristic

Two first-degree relatives, both with skin phototype I, freckles, green eyes, red hair, over 150 melanocytic nevi, and intense photodamage, had their first CM with a mean age at diagnosis of 46.5 (SD: 20.5 years). They started TBM after PM diagnosis (daughter in June and father in July 2018). The number of digital dermoscopy positions in the first and last mapping were, respectively, 455 and 689 for the female and 172 and 313 for the male. The mean number of nevi that had digital dermoscopy performed at baseline was 313.5 (SD: 200.1 nevi). The female was followed for 56 months, while the male had a 51 months follow-up, with a mean of 53.5 (SD: 3.5 months). Among other findings in Table S1, one of them corresponds to the total number of melanomas excised in the period (53 in the daughter and 7 in the father) and this finding will be further analyzed.

With respect to genetic testing, in November 2018, thru saliva sample, the genomic DNA of the daughter was isolated and a customized genetic panel, including *ACD*, *BAP1*, *CDKN2A*, *CDK4*, *POT1*, *TERT*, *TERF2IP*, *MITF* and *MC1R* genes. No pathogenic variants known for hereditary melanoma were identified, however the female patient showed heterozygosis of two variants in the *MC1R* gene, conferring a melanoma risk. In June 2022, thru peripheral blood sample, an expanded genetic panel for hereditary cancer predisposition was performed, analyzing 141 genes. Likewise, no pathogenic variants were identified, further variants of undetermined clinical significance (VUS) have been identified in the *MEN1* and *MSH3* genes. No variations in copy number (CNVs) were detected. Both father and the father’s sister did their first genetic testing for isolated *MEN1* gene, from peripheral blood, in April 2023, and no pathogenic variants were found. In July 2023, the father underwent a customized analysis of 30 genes thru peripheral blood sample: *BAP1*, *BRCA1*, *BRCA2*, *CDK4*, *CDKN2A*, *CHEK2*, *EZH2*, *FDX1*, *GNAQ*, *GNAS*, *HRAS*, *KIT*, *KRAS*, *MC1R*, *MITF*, *MLH1*, *MSH2*, *MSH3*, *NBN*, *NOTCH1*, *NRAS*, *PALB2*, *PMS2*, *PTEN*, *RAD51D*, *RB1*, *SMO*, *TERT*, *TP53* and *TYR*. No high-risk genes, nor VUS and no CNVs were reported. He bears heterozygosis of two variants in the *MC1R* gene, the same ones that his daughter carries. The genetic profile of MPM studied patients are in Table 2.

### 3.2. Descriptive Analysis of Excised Melanomas

According to the results in Table 3, the ages at first diagnosis, for the daughter and father, were 32-year-old and 61-year-old respectively. Both had a higher percentage of SPM in the lower limbs (daughter: 45.3%; father: 42.9%). Regarding laterality, the daughter had a higher percentage of SPM on the left side (67.9%) while the father had them on the right side (57.1%) of the body.

**Figure 5:**
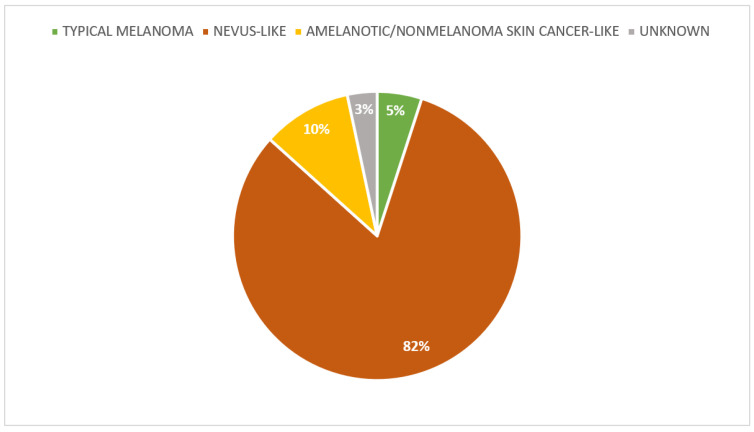
Distribution of three morphological clusters (clinical patterns) found on 60 cutaneous melanomas, for both genders.

In our cohort of 58 SPMs, the most prevalent clinical pattern was nevus-like (daughter: 83%; father: 71.4%). Conversely, we didn’t find tumors that clinically had a morphology of a seborrheic keratosis-like nor lentigo/lentigo maligna-like (Figure 5). The predominant pigmentation pattern was hypopigmented (daughter: 40.4%; father: 83.3%). Patients had a higher percentage of flat (non-palpable) melanomas (daughter: 60.4%; father: 83.3%). Most SPM showed ill-defined borders (daughter: 63.5%; father: 100%). Figure S1d,e shows a “de novo” in-situ SPM with a nevus-like clinical pattern. The tumor is flat, ill-defined, and hypopigmented.

**Figure 6:**
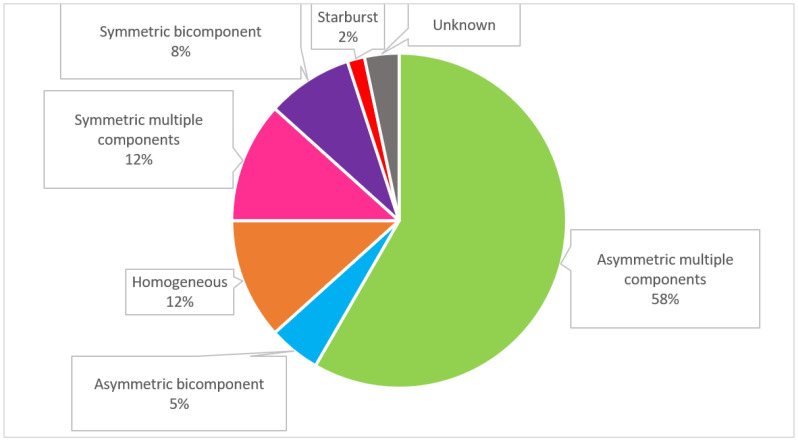
Distribution of global dermoscopic patterns found on 60 cutaneous melanomas, for both genders.

**Figure 7:**
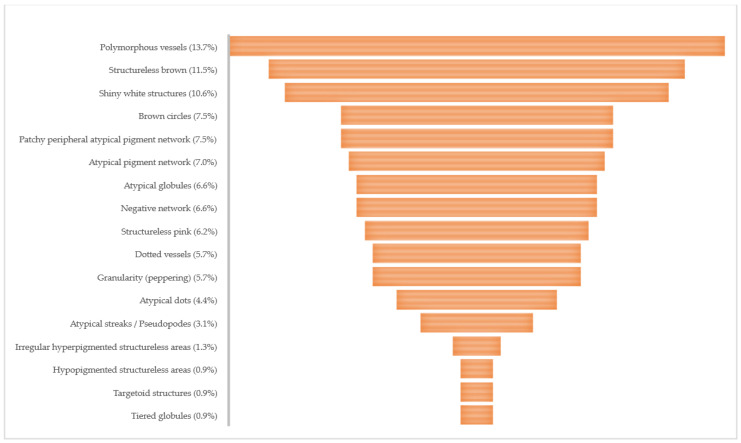
Frequency of 17 melanoma-specific dermoscopic structures found, in both in-situ and invasive melanomas, on our cohort of 58 SPMs.

**Figure 8:**
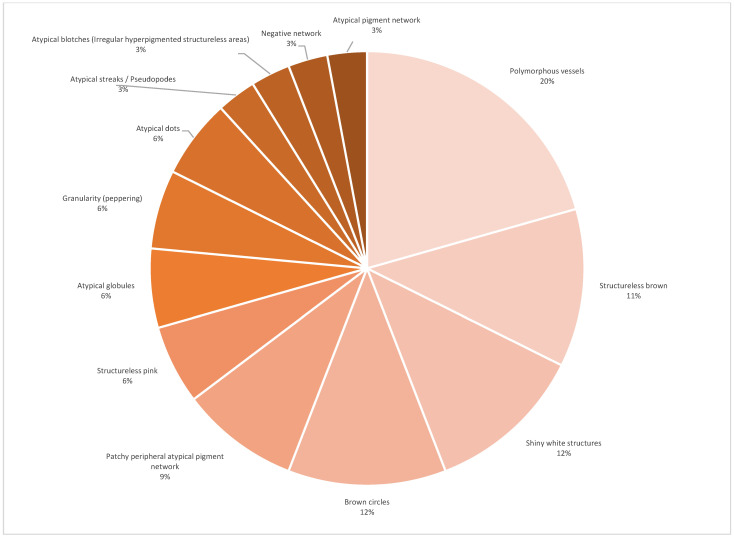
Frequency of 13 melanoma-specific dermoscopic structures found on our cohort of 10 invasive SPMs.

The global dermoscopic patterns with the highest frequency for SPM were asymmetric multiple component for the daughter (64.2%) and symmetric multiple component for the father (42.8%). Figure 6 shows the distribution of global dermoscopic patterns of 60 melanomas for both genders. From the list of thirty dermoscopic melanoma specific structures, we found 17 (57%) structures to suspect melanoma (Figure 7). When only invasive SPM were analyzed the number of visualized melanoma structures decreased to 13 (43%) (Figure 8). In our cohort, the total mean number of melanoma-specific structures per tumor was 3.9 with a standard deviation of 1.2. None of the of 58 SPMs showed angulated lines, prominent skin markings, multiple small hyperpigmented areas, raised blue-white structureless area/blue-white vail, flat blue-white structureless area, regression scar-like depigmentation, structureless blue, and structureless blue black nodule.

The mean time of SDDI follow-up (months between first and last dermoscopy) to suspect a melanoma was 19.4 months (SD: 16.6 months). RCM was performed prior to the excision of 36.7% of diagnosed SPM (daughter: 39.6%; father: 14.3%). The daughter had 85.7% of MCR suggestive of malignancy, while the father had none. The mean time from the last dermoscopy to excision was 1.1 month (SD: 1.1 month).

On the pathology report, the mean size of SPM was 6 mm (SD: 2.4 mm). Histologically, in-situ melanoma (daughter: 84.9%; father: 71.4%) were the majority of SPM diagnosis. The most prevalent histologic type was superficial spreading melanoma (daughter: 98.1%; father: 85.7%). Figure S3 shows the only in-situ NAM, lentigo maligna type, on the daughter and Figure S4 displays the single in-situ NAM, lentiginous type, on the father. Eighty-one percent of NAMs occurred on the daughter (juncional: 7.5%, compound: 35.8%, dermal: 32.1% and dysplastic: 5.7%) and most “de novo” melanomas on the father (57.1%). From Figures S5–S10 examples of NAM and “de novo” SPMs are presented. The female patient had one melanoma on the neck (Figure S8) and one on the perineal area (Figure S9). SPM with histological regression was rare (daughter: 7.5%) or completely absent (father: 0%), case shown on Figure S3. But then, histological intratumoral inflammatory infiltrate was frequent (daughter: 83%; father: 57.1%). Figure S11 illustrates two cases of CMs, one in-situ and one invasive, with histological inflammatory infiltrate and no regression. On the other hand, Figure S12, demonstrates two cases of CMs, both in-situ, without histological inflammatory infiltrate nor regression. All 7 melanomas on the father had pathological tumor-free margins after excisional biopsy, but the daughter had four (7.6%) melanomas with compromised/coincident margins. Wide local excision was accepted by both patients at the beginning of follow-up and for SPM diagnosed at the time. When the number of SPM diagnosis increased wide local excision were always discussed, yet they were refused by the daughter for 83% of diagnosed SPM and the father refused 57.1% of re-excisions.

### 3.3. Correlation Analysis of Excised Melanomas

**Figure 9:**
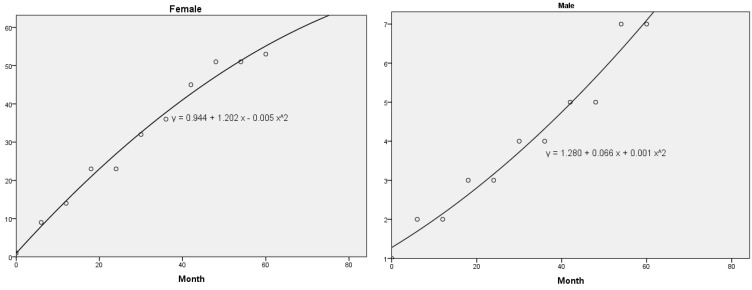
Curves of the quadratic model for daughter and father: number of melanomas as a function of time (in months).

Table S2 presents the accumulated number of melanomas in 5 years (60 months) for father, daughter, and both. Based on these data, some models (linear, quadratic, and cubic) were adjusted to estimate the cumulative number of melanomas as a function of time (in months). For father and daughter, the three models fit very well with R^2^ above 0.95 (95%). Figure 9 shows the quadratic model that corresponds to y = 0.944 + 1.202 x − 0.005 x^2 for the female and to y = 1.280 + 0.066 x + 0.001 x^2 for the male. One year from now the number of melanomas expected (x = 60 months + 12 months = 72 months) corresponds to approximately a total of 62 CMs (x = 1 PM + 52 diagnosed SPMs + 9 estimated new SPMs = 62 CMs) for daughter and 12 CMs (x = 1 PM + 6 diagnosed SPMs + 5 estimated new SPMs = 12 CMs) for the father.

We performed several tests to observe correlations among time of SDDI follow-up amid clinical, dermoscopic and RCM variables. There was statistical significance among time of SDDI follow-up and RCM performed (*p* = 0.002). When a suspicious melanocytic lesion under SDDI was selected for RCM evaluation, the mean follow-up was 10 months (SD: 12.2 months), conversely diagnosed melanomas without the aid of RCM had a mean follow-up of 25.2 months (SD: 16.4 months) (Table 4). Figure S13 shows an example of a nevus-like, “de novo” invasive SPM that had a follow-up of 6.9 months. When RCM was performed the lesion showed features of malignancy, being immediately excised. Figure S14a–c displays a nevus-like, in-situ NAM that didn’t have RCM done and was followed for 22.9 months.

Correlation between time of SDDI follow-up and NAM showed statistical significance (*p* = 0.016). The mean time of follow-up to diagnose a melanoma associated with a dysplastic nevus was 41.7 months (SD: 3 months), significantly greater than all other mean times of NAM follow-up, shown on Figure S14d–f. There was no correlation within other nevi type, nonetheless the mean time of follow-up to diagnose a melanoma associated with juncional nevus was 11.7 months (SD: 13 months), with compound nevus was 14.2 months (SD: 13.6 months), with dermal nevus was 23.9 months (SD: 16 months) and not associated with nevus (“de novo”) was 17.1 months (SD: 19.1 months) (Table 5).

We found correspondence among time of SDDI follow-up and SPM with histological presence of intratumoral inflammatory infiltrate (*p* < 0.001). The mean SDDI follow-up time of SPM without histological inflammatory infiltrate was 37 months (SD: 9.7 months), significantly longer than the mean SDDI follow-up time of 14.8 months (SD: 14.9 months) for SPM with histological intratumoral inflammatory infiltrate (Table 6). Figures S11 and S12, respectively.

We didn’t find a statistically significant correlation between time of SDDI follow-up and clinical pattern of the melanoma (*p* = 0.633, Table S3), global dermoscopic pattern (*p* = 0.632, Table S4), melanoma pigmentation pattern (*p* = 0.338, Table S5), melanoma palpability (*p* = 0.110, Table S6), melanoma borders (*p* = 0.270, Table S7), MCR suggestive of malignancy (*p* = 0.764, Table S8), the size of the melanomas in the pathology reports (*p* = 0.228, Table S9), diagnosis and Breslow thickness of melanoma (*p* = 0.731, Table S10), and histological regression of the melanoma (*p* = 0.866, Table S11).

Tables S12–S14 demonstrate lack of correlation among clinical pattern, global dermoscopic pattern, and body location with both histological diagnosis of invasive melanoma (Breslow thickness <1 mm and >1 mm) and in-situ melanoma. Nevertheless, we found a higher percentage of SPM with clinical nevus-like morphology (84.5%), with a predominant global dermoscopic pattern of asymmetric multiple component (60.3%) and located on the lower limbs (45%). Regarding laterality, Table S15 shows a higher frequency of SPM on lower limbs (46.6%) of either side (left: 51.3%; right: 36.8%). Figure S15 shows two adjacent NAM, one in-situ and one invasive, on the right lower limb. Figure S16 presents the thickest invasive “de novo” SPM on the female patient popliteal region.

Correlation tests between time of last SDDI follow-up to excisional biopsy (months) were not significant for SPM location (*p* = 0.783, Table S16), for clinical pigmentation (*p* = 0.303, Table S17), and for lesion palpability (*p* = 0.437, Table S18).

According to the results in Table S19, at a significance level of 5%, only clinical pigmentation (*p* = 0.035) and nevus-associated melanoma (*p* = 0.001) variables presented a significant relationship with pathological diagnosis (in-situ × invasive melanoma). Patients with invasive melanoma had a significantly higher percentage of clinical hypopigmented SPM (77.8%), while patients with in-situ melanoma had most of SPM distributed in hypopigmented and pigmented categories (71.5%). Furthermore, 22.2% were amelanotic invasive SPM and neither patients had pigmented or hyperpigmented invasive SPM. For both patients, with respect to NAM, a significant number of in-situ SPMs were associated with common nevus (junctional, compound, and dermal) whereas most invasive SPMs were “de novo”, not associated with nevus (Figures S17–S21).

As well, at a significance level of 10%, clinical pigmentation and NAM are the only variables that presented a significant relationship with pathologic diagnosis. Thus, a multivariate logistic regression model was built to verify the relationship between pathologic diagnosis and both variables simultaneously. The results of this model are in Table 7.

At the 5% significance level, only the NAM variable continued to show a significant relationship with pathologic diagnosis in the multivariate model (*p* = 0.006). The clinical pigmentation variable lost statistical significance mainly because none of the invasive SPMs had clinical pigmented or hyperpigmented appearance. This made it not possible to calculate the chances of diagnosing an invasive SPM regarding the pigmentation variable. On the other hand, the chance of a nevus-associated invasive SPM diagnosis is 0.04 times greater than the chance of a not associated with nevus (“de novo”) invasive SPM. That is, in our cohort, the chance of a “de novo” invasive SPM diagnosis is 25 times greater than the chance of a diagnosis of a nevus-associated invasive SPM (Figures S22 and S23).

According to the results in Tables S20–S22, at a significance level of 5%, there are no significant differences between father and daughter in relation to the amount of melanoma specific structures (*p* = 0.305), nevus-associated melanoma in terms of tumor location (*p* = 0.412), and nevus-associated melanoma in relation to histological regression (*p* = 1.000).

## 4. Discussion

Day-to-day practice on skin cancer clinics involves the challenge of CM early diagnosis. High-risk patients (i.e., patients with a personal or familial history of melanoma, extensive photodamage, high nevus count, nevi of variable colors and/or multiple sizes) may be carriers of mutations in either moderate/low or high melanoma susceptibility genes. The opportune acknowledgment of PM and MPM thru clinical exams defy clinicians, because naked-eye recognition of early melanomas, that mimic moles, can be difficult, even more so when patients have multiple nevi, making excision not feasible or desirable [6,16,42,43]. The advent of dermoscopy, TBSP, DD, SDDI and RCM has been an enlightening path for dermatologists dedicated to skin cancer diagnosis, treatment, and surveillance. The tie of skin imaging technologies with cutaneous cancer diagnosis is a reality. For over three decades they have influenced how dermatologists perceive benign or malignant pigmented and non-pigmented lesion in correlation to phototype, phenotype, and genotype of patients [9,12,16,23,37,44,45,46,47,48,49,50].

From February 2018 to May 2023, we prospectively studied two first-degree relatives with fair skin, history of multiple blistering sunburns in childhood and adolescence, an exceedingly high number of melanocytic nevi, and one primary melanoma at baseline each. They weren’t exposed to artificial tanning beds. Both patients were followed by TBSE, handheld dermoscopy, TBSP, DD, SDDI and RCM. The use of imaging technologies was of fundamental aid for early diagnosis. Guitera et al., in a cohort of 171 new melanomas detected among 593 patients, acknowledged that the use of TBSP and SDDI assisted in the diagnosis of 67% of CM. They detected a Breslow thickness < 1 mm for 96% of CM. Regarding MPM there was a predilection for male patients, and they found up to 6 tumors in affected individuals [43].

During the study intervention, father and daughter were compliant with consultations and with excisional biopsies of suspect lesions. A total of 214 full thickness biopsies with 2 mm margins were performed, most in a private ambulatory setting and a few in a hospital setting, yet all excised lesions were sent to ACCCC pathology laboratory to be read by two or more pathologists specialized in skin tumors (Figure 2). Our cohort is composed of 60 melanomas (2 PMs and 58 SPMs) diagnosed within a 5-year period, with a higher number of MPM for the female patient. According to the eight edition of the American Joint Committee on Cancer (AJCC), our MPM series has fifty in-situ CM (T0: 83%) and ten invasive CM (pT1a: 17%). Marghoob et al. revealed an inverse association between the total nevus count and CM thickness, demonstrating that patients with a higher number of nevi had thinner CM and more in-situ CM, independently of sex and age [51].

The melanoma “signature” in Brazil was reported by de Melo et al., demonstrating a higher percentage of female patients (51.9%), between 40–69 years old (59.4%), and from Southeastern Brazilian states (47.3%). There was a prevalence of white skin color (75%) and melanomas diagnosed at stage I and II (53.2%) [52]. The Cancer Observatory of ACCCC, a hospital cancer registry (HCR), discloses information on cases of cancer diagnosed and treated at the Institution. From 2000 to 2020, a total of 98,711 cases of cancer were diagnosed in patients treated at ACCCC. In the proportion distribution of cases, skin tumors occupy the first position, comprising 32.2% of the institution’s cancers. Melanoma was the third most frequent neoplasm for men, with 2752 cases, behind non-melanoma skin cancer (NMSC) and prostate cancer. For women, melanoma was the sixth most frequent tumor, with 2522 cases, behind breast, NMSC, thyroid, cervix, and colon/rectum tumors. The CM estimated global survival in 5 years, for the period between 2000 and 2017, by clinical stage I and by gender, was 96.5% for women and 95.3% for men. For stage IV, survival dropped to 30.7% and 41.2% for female and male respectively [53].

A 22-year-period Australian cohort of 2057 melanoma cases found MPM in 4.8% of studied individuals. Of those nighty-nine patients, only 46 patients met inclusion criteria, with a total of 114 MPM and an average number of 2.5 CM per patient (range: 2–10 MPM per patient) [54]. Previous studies show an up to 59% of SPM occurring within the first year following a PM diagnosis. Furthermore, a cumulative incidence of postbaseline incident melanomas diagnosed was significantly higher in the first two years of follow-up [6,55]. The risk of additional PM was studied in an Australian population-based cohort of 2613 PM, 16.5% in-situ and 83.5% invasive (of which 68% had a Breslow thickness of ≤1.0 mm). The number of primary melanomas per individual ranged from one to 16. The median time between primary melanomas decreased with each new SPM, from 3.7 years between the first and the second melanoma to 0.7 years between the sixth or higher order and subsequent tumors [56]. Moloney et al., in a prospective 5-year follow-up study, found a 12.7% risk of new PM development by year 2, and 18.2% by year 4 [6].

El Sharouni, et. al, in a cohort study of 2284 patients with 4967 MPM in the Netherlands, encountered a 4% prevalence of MPM, in line with the literature range of 0.2% to 12.7%. Only 15% (339) patients had synchronic MPM. A total of 36.8% of SPMs were found during the first two years of follow-up, and 27.3% of second MPM more than five years after diagnosis of the PM, with a maximum time of 16.7 years between the first and second CM. They found two up to ten SPMs diagnosed per patient. Regarding stages, 48.7% of SPMs had the same, 35.1% had lower, and 16.2% had higher T stage in relation to the first PM. Breslow thickness was significantly decreased for SPMs, but the likelihood of dying was 31% higher among patients with MPM, a statistically significant finding [1].

In our cohort, the proportion of melanomas diagnosed during the 5-year intervention is seen on Figure 3. The daughter had a total of 53 MPM (PM: 1, SPM: 52) diagnosed by age 37, and of those 8 were synchronic tumors (Figure 4). The father had a total of 7 MPM (PM: 1, SPM: 6) by age 65. Fifty-seven percent of SPMs were diagnosed in the first 2 years of intervention and 40% from 2021 to the present time, showing that there is an ongoing diagnosis of both NAM and “de novo” SPM, in accordance with previous studies [6,56].

The coexistence of nevus and melanoma components on histopathological examination of a melanocytic lesion represents the NAM group, that is known to account for approximately one-third of all CM, whereas “de novo” melanoma (DNM) represents two-thirds of CM [57,58,59,60,61]. NAMs were more frequent on the female (43, 81.1%) and “de novo” melanomas the majority on the older male (4, 57.1%). Combining both genders, our MPM cohort presented 76.7% NAM and 23.3% DNM, an inverse distribution when compared to recent literature of 24.1 to 29.7% NAM and 70.3 to 75.9% DNM [57,58,62]. Other studies demonstrated prevalence of NAM ranging from 8.4 to 85% [60,63,64].

A significantly lower mean Breslow thickness for NAMs than DNMs has been described in literature [5,58,65]. It has been discussed that it’s extremely difficult or even impossible to determine if the lesion had originally been associated with a nevus or not in all DNM cases [58,60,65]. NAM and DNM may just be distinct subtypes and not different due to the obliteration of nevus remains in thicker melanomas [62]. The male and older patient had the majority of “de novo” SPM (in-situ: 2.50%; invasive < 1 mm: 1.25%; invasive > 1 mm: 1.25%). He had three in-situ NAM (associated with compound, dermal and dysplastic nevus) and no invasive NAM. In our cohort, the chance of a “de novo” invasive SPM diagnosis is 25 times greater than the chance of a diagnosis of a nevus-associated invasive SPM. Figures S8e–h, S13, S16, S21, S22, and S23 show examples of “de novo” invasive SPM, revealing that strict monitoring of high-risk patients with TBM is indispensable, regardless of age and gender.

The literature indicates that NAM is often seen on younger patients with multiple atypical nevi, generally superficial spreading melanoma histological subtype, and has a lower Breslow thickness and better prognosis compered to DNM [57,58,60,65], a description that fits our female patient. Her high frequency of NAMs were associated with both common and atypical nevus types (juncional: 4, 7.5%; compound: 19, 35.8%; dermal: 17, 32.1%; dysplastic: 3, 5.7%). Of those NAMs, 40 (93%) were in-situ and 3 (7%) were invasive (2 associated with dermal nevi, one with dysplastic nevus, both <1 mm Breslow thickness). Figures S3, S6, S7, S8a–d, S11e–h, S14 and S15 are examples that help understand the evolution of NAMs, demonstrating that SPM in patients with MPM do become invasive and need early diagnosis and intervention. Literature highlights that the majority of NAMs develop in common acquires nevi, with predilection for dermal nevi, a type of nevus that usually develops in early childhood [5,58]. Conversely, a high proportion of melanomas associated with dysplastic nevi (DN) has also been found [6,61].

Considering both patients, we accomplished a NNT of two benign lesions excised for each skin cancer (Table 1), a number in accordance with most specialized skin cancer hospitals and clinics, yet higher than the benign to malignant excision ratio of 0.8:1.0 reported by Guitera et al. [43]. A systematic review of 46 articles on the number-needed-to-biopsy (NNB) metric for all biopsied tumors found a mean worldwide NNB of 15.6, and a range of 2.2 to 287, demonstrating a wide variation within geographic locations and clinicians [66]. The number needed to excise (NNE) is a ratio of the number of excised benign nevi for each diagnosed melanoma [40]. In a meta-analysis, doctors specialized in skin cancer had the lowest NNE of 5.85, followed by general dermatologists (NNE = 9.6) [67]. Nelson et al. found a lower worldwide NNE of 7.5 for all dermatologists [66]. For the period of our follow-up, combining both genders the NNE was 2.3 acquired nevi excised for each diagnosed CM, nearly the same reported by Guitera et al. of 2.4:1.0, and lower than most ratios described in literature [43,66,67], confirming a better specificity of the dermoscopic comparative approach [37]. The NNE for the female patient was 1.7 nevus for each melanoma, whereas for the father we found a higher NNE ratio of 6.9 (Table 1). We hypothesize that the difference is due to older age, more extensive sun damage, presence of body hair, and multiple benign and malignant non-melanocytic skin lesions.

NNE is of limited utility as a proxy for estimating CM diagnostic efficacy without also considering the Breslow thickness of excised melanomas for a specified number of benign moles removed [40]. The in-situ/invasive ratio (IIR) is the proportion of in-situ/invasive (combined <1 and >1 mm Breslow thickness) melanomas diagnosed per patient, to determine whether a smaller NNE is associated with a higher proportion of invasive melanomas. In our cohort high IIRs for in-situ melanomas were achieved (female: 84.9%; male: 71.4%) showing that early melanomas weren’t missed, as well as invasive melanomas were being diagnosed early (Table 1).

Karapetyan et al. studied 330 patients with a median age of 51 years and found that, compared to patients with a single melanoma, MPM individuals were younger at diagnosis of their first PM, more likely stage 0, or I, with family history of melanoma, atypical moles, dysplastic nevi, and had indoor tanning exposure. In a multivariate analysis family history of CM, presence of atypical/dysplastic nevi, recreational sun exposure, and indoor tanning remained associated with the occurrence of MPM [55]. It’s been postulated that the production of reactive oxygen species and immunosuppressive cytokines in the setting of UV-induced DNA damage favors epidermal cell mutation, an immunosuppressive stromal environment, and tumor progression [68].

Melanoma can mimic many cutaneous lesions, impairing correct diagnosis. Awareness of the diversity of melanoma appearances and their associated clinical factors may help dermatologists improve their diagnostic accuracy [34]. The spectrum of melanoma morphologies is unexpectedly diverse, which may have implications for visual clinical diagnosis, possibly requiring a more specific training. Klebanov, et al. have identified five common melanoma morphological clusters: typical melanoma (A: asymmetry, B: irregular borders, C: multiple colors, and D: diameter > 6 mm), nevus-like melanoma, amelanotic/NMSC-like melanoma, SK-like melanoma, and lentigo/lentigo maligna-like melanoma [34]. In our cohort only three morphologies were present and the most frequent was nevus-like melanoma, 83% and 71.4% in female and male respectively. We didn’t find SPMs with seborrheic keratosis-like and lentigo/lentigo maligna-like clinical morphology. A distribution of SPMs clinical patterns can be seen in Figure 5.

The literature on melanoma tumor site is vast, with a known predisposition for lower limb and trunk for women and men, respectively [1,2]. Conversely a 5-year study of 311 high-risk melanoma patients found most PM on the trunk followed by upper limbs in both genders [6]. In Brazil, PM are diagnosed in the trunk (27.1%) followed by lower limbs/hips (26.2%), head and neck (19%), and upper limbs/shoulders (14%) [52]. A recent study of 93,729 melanoma patients, from January 2016 to December 2019, found the upper limb and trunk as the most common anatomical location for female and male respectively. An additional finding was that women have a three times higher predisposition for CM on the breast, peri-anus, and lower limb including hip regions. In contrast, female patients were more than 50% less likely to have CM on the scalp, neck, and ears [69]. The estimated probability of the locus of the SPM being the same as that for the PM is 34% [55]. Concerning body site, patients with *RHC-MC1R* variants tend to develop melanomas mainly on the trunk and the arms [9]. In our prospective cohort, SPMs were mostly located on the lower limbs (female: 24, 45.3%; male: 3, 42.9%), followed by dorsal and ventral trunk combined (female: 18, 34%; male: 2, 28.6%), and upper limb (female: 8, 15.1%; male: 2, 28.6%). Only the female patient had melanomas on special sites (perineal: 1, neck:1, acral–dorsum of the left foot: 1). Regarding laterality, the daughter had a higher percentage of SPMs on the left side (67.9%) while the father had them on the right side (57.1%) of the body, not statistically significant.

In our findings MPM ranged from 3 to 15 mm with a mean size of 6 mm (SD: 2.4 mm), measured on the pathology report. Melanomas with smaller diameters have been reported to be frequently thin, however in a 537 invasive melanoma case-series, Dessinioti et al. found 57 (10.6%) small-diameter invasive melanomas, with a median Breslow thickness of 0.8 mm. Of those, 5 (8.9%) presented with metastasis [70]. Nazzaro et al. studied 103 melanomas ≤ 5 mm in diameter, 44 (43%) in-situ and 59 (57%) invasive mini-melanomas [71]. We found a majority of in-situ MPM (daughter: 84.9%; father: 71.4%), in relation to thin invasive MPM with a Breslow thickness < 1 mm (daughter: 15.1%; father: 14.3%) and invasive MPM with a Breslow thickness > 1 mm (father: 14.3%). Invasive melanomas were more frequent in the lower limbs (40%). Dessinioti et al. found that DNMs on the lower limbs were more likely to occur in female patients [62]. The daughter thickest SPM was on the posterior leg (Figure S16), however the father’s thickest invasive SPM occurred on the lateral arm (Figure S23). The proportion of in-situ MPM was higher in our cohort than in a previous prospective study, from 2006 to 2009, by Moloney et.al. that found 51% of in-situ PM, 40% of thin invasive PM (Breslow thickness < 1 mm), and 9% of invasive PM (Breslow thickness > 1 mm) postbaseline incident melanomas excised [6]. The most prevalent histologic type was superficial spreading melanoma (daughter: 98.1%; father: 85.7%). The only additional subtypes found were one extra-facial lentigo maligna (Figure S3) and one lentiginous melanoma (Figure S4).

For both patients, in-situ SPMs were 61.2% amelanotic/hypopigmented and 38.8% pigment/hyperpigmented. Regarding invasive SPMs one-hundred percent were pink/tan lesions (amelanotic: 22.2%; hypopigmented: 77.8%). In the univariate analysis pigmentation presented a significant relationship with pathological diagnosis, 35 (71.5%) of in-situ SPMs were either hypopigmented or pigmented, while seven (77.8%) of invasive SPMs were hypopigmented. In the multivariate analysis, clinical pigmentation lost statistical significance with pathologic diagnosis. A population-based study established an amelanotic CM frequency of 8% within the range of 2% to 20% reported in the literature. The authors found an association with older age, head/neck site and sun-damaged skin. Lack of a coexisting nevus was independently associated with amelanotic CM. In addition, they demonstrated that CM with higher AJCC tumor stage were more likely to be amelanotic. A 5-year melanoma-specific survival of 88% in amelanotic CM and 95% in pigmented CM was found [72].

In previous studies, no association was found between the *MC1R* variants and high counts of acquired nevi in families with hereditary melanoma [9,73]. Carriers of RHC-*MC1R* variants generally develop hypopigmented melanocytic lesions, called “white nevi” or “red melanomas” and display larger nevi and melanomas [9]. Both patients presented mostly with tan/skin-colored and pink lesions. In addition, many common and dysplastic nevi shared the same color pallet. The father had 83.3% clinically hypopigmented SPMs. The daughter had 40.4% hypopigmented and 25% amelanotic SPMs, that is, most of her melanomas (65.4%) had pink or tan shades. Physicians and even more so dermatologists should be aware that melanomas can arise in association with old and stable dermal nevi, and consequently, they should never forget to examine such nevi by dermoscopy during routine visits [58].

Due to the high number of nevi (>450), the daughter had more frequent follow-ups in other to be possible to preform DD and SDDI of most melanocytic lesions. Patients with many nevi may have a higher frequency of NAMs [5,74]. These patients could thus benefit from sequential digital dermoscopy in addition to total-body photography [63]. Moloney et al. found a median (range) time from baseline visit to PM detection of 17.9 months [6]. Our mean time of SDDI follow-up to suspect a new melanoma was 19.4 months (SD: 16.6 months). To detect a DNM, present at baseline, the mean time of SDDI follow-up was 17.1 months (SD: 19.1 months). When a “mole” is present at baseline imaging, there was a very similar time range of follow-up to detect a NAM or DNM. Conversely, diagnosis of a melanoma associated with a dysplastic nevus had a mean follow-up time of 41.7 months (SD: 3 months), significantly greater than all other NAM follow-up intervals. We found dysplastic nevi associated melanoma more difficult to diagnose by dermoscopy. Pampena et al. showed NAMs were independently more associated with a longer follow-up than DNMs [57]. That is in line with the position to excise suspect lesion, since a dysplastic nevus is a histological diagnosis and dermoscopy is less helpful sorting DN from CM [20,75]. We found correspondence among time of SDDI follow-up and SPM with histological presence of intratumoral inflammatory infiltrate. The mean SDDI follow-up time of SPM without histological inflammatory infiltrate was 37 months (SD: 9.7 months), significantly longer than the mean SDDI follow-up time of 14.8 months (SD: 14.9 months) for SPM with histological intratumoral inflammatory infiltrate. A finding that warrants exploration in future studies. SPMs with histological regression were rare (daughter: 7.5%) or completely absent (father: 0%), in accordance with the finding that DNM was less likely to have regression present when compared to NAM [62].

Reiter et. all followed a total of 106 patients, phototype I or II, for a median time of 16.4 years. In their population, 71 (67%) patients had a history of melanoma. Contrary to prior reports, they found that most nevi in adults increase in diameter, while few nevi shrink. Fading at the center of the lesion was noted in 15% of nevi that grew compared with 35% of nevi that shrunk. Nevi appear to have long-term changes and most even-growing nevi do not necessarily point to malignancy [76]. Nevi growth decreased as patients aged, on the other hand, disappearance of nevi was not related to age and this phenomenon was uncommon even among the oldest participants. New nevi appear in 10–33% of adults, yet in higher numbers among young patients. Women had 46% fewer new nevi than men per 15 years, that finding was statistically significant. High-risk patients acquire new nevi throughout life with very few nevi disappearing over time. In addition, it appears that nevus volatility is associated with melanoma. One possible explanation is that patients who develop melanoma harbor a genetic predisposition for melanocytic proliferation, which also affects the melanocytes in their nevi. Another reason could be that the development of melanoma induces systemic effects, such as immunological, which lead to changes in the patient’s nevi [76].

In our cohort, if a new lesion or a symmetrically enlarging former nevus didn’t show suspicious dermoscopic patterns it was included for DD follow-up. Excision was immediately advised for new palpable lesions, new flat lesions with melanoma specific dermoscopic features, and existing nevi with irregular growth and/or gain/loss of dermoscopic suspect structures, independent of the nevi pigmentation pattern. The mean time from the last dermoscopy to excision was 1.1 month (SD: 1.1 month). All 7 melanomas on the father had pathological tumor-free margins after excisional biopsy, but the daughter had 4 (7%) melanomas with compromised/coincident margins. Wide local excision was performed for 17% of the female SPMs and for 42.9% of the male SPMs.

Considering lesion palpability, a higher percentage of SPMs were flat lesions (daughter: 60.4%; father: 83.3%). Moreover, the amount of palpable dermal nevi is high for both patients. Under dermoscopy, using the comparative approach, dermal nevi tend to look alike. On clinical practice, they frequently are regarded as common dermal nevi and scape DD follow-up. In our series, the one melanoma diagnosed by the patient (she requested excision) during SDDI was a mimicker of a common dermal nevus, without melanoma specific dermoscopic patterns, a melanoma that scaped physician’s diagnosis (Figure S8a–d). In toto the daughter found two melanomas (PM: 1 and SPM: 1), she further requested excision of 16 nevi. The PNNE (patient number needed to excise) of 8 common nevi to each melanoma diagnosed was much higher than the dermatologist’s NNE of 1.5 (nevi: 76 and SPM: 51), collaborating with the importance for in person dermatological consultations of high-risk patients. It has been discussed that a great proportion of patients were able to detect their first PM, yet history of a previous CM was not associated with an increase in the ability of patients to detect SPMs themselves. As well, literature shows that melanoma patients who skipped follow-up visits had a significantly increased Breslow thickness for their second melanoma. Up to 95% of SPMs are detected by physicians, thus lower Breslow thickness may be associated with dermatologic surveillance [1].

In this prospective study of two high-risk patients cohort, most SPMs showed ill-defined borders (female: 63.5%; male: 100%) and were flat lesions (Figures S5–S7, S14, S16, and S17 are a few examples). There is a predominance of “complex” global dermoscopic pattern (defined as multicomponent) in patients diagnosed with CM [12,35,59]. Rishpon et al. found multicomponent pattern, including the presence of network and globules in the same nevus, to be associated with melanoma [77]. The observation of eccentric hyperpigmentation and formation of globules have been described as well for carriers of RHC-*MC1R* variants [9]. A systematic review and meta-analysis on dermoscopic patterns and structures for melanoma detection found a high sensitivity for global multicomponent pattern [36]. Nonetheless, it’s been described a significant number of melanoma patients with predominant reticular and homogeneous patterns, which are less often associated with an increased risk [35,65]. For our study we classified melanocytic lesions as either symmetric or asymmetric bicomponent (2 dermatoscopic structures), symmetric or asymmetric multicomponent (3 or more dermatoscopic structures), homogeneous, and starburst. Figure 6 presents the global dermoscopic patterns distribution of our cohort of 60 cutaneous melanomas, for both patients. The two global dermoscopic patterns with the highest frequency for SPMs were asymmetrical multicomponent for the daughter (65.4%) and symmetrical multicomponent for the father (42.9%).

We retrieved thirty melanoma-specific dermoscopic structures from literature [23,36,38,45,78,79,80,81,82,83,84,85,86]. The attending dermatologist selected up to six present melanoma-specific dermoscopic clues for each case (58 SPMs). Only 17 (56.7%) of known structures were appointed and their frequency are shown on Figure 7. The ten most prevalent structures were polymorphous vessels, structureless brown, shiny white structures, brown circles, patchy peripheral atypical pigmented network, atypical pigmented network, atypical globules, negative network, structureless pink, and dotted vessels. Reiter et al. found NAMs were 2.5 times more likely to show negative pigment network than DNM, and that in-situ NAMs had twice more structureless areas than DNM [65]. Nazzaro et al. identified two dermoscopic predictors of invasiveness of flat, non-facial, mini-melanomas, namely: blue white veil and negative pigment network. Further, an atypical vascular pattern was present only in invasive melanomas but didn’t reach statistical significance [71]. In our cohort of ten invasive SPM visualized melanoma dermoscopic structures decreased to 13 (Figure 8). The five most prevalent were polymorphous vessels, structureless brown, shiny white structures, brown circles, and patchy peripheral atypical pigmented network. These findings probably differ from literature because the totality of invasive SMP were pink/tan lesions of both small and larger diameters.

We found a mean 3.9 (SD: 1.2) melanoma-specific features per lesion, confirming the need to suspect malignancy when one or more melanoma specific dermoscopic structure is present. Ramji et al., studied 124 in-situ CM and found that they predominantly increased in morphological complexity over time, displaying more than 3 clues to malignancy [50]. It’s described in the literature that *MC1R* variants, especially the RHC, modulate the color and the dermoscopic pattern of CM and nevi, resulting in hypopigmentation and the presence of fewer dermoscopic structures, which emphasizes the vascular pattern of these tumors [9,44].

Monitoring is not recommended for lesions with specific melanoma dermoscopic features nor nodules. TBSP and SDDI is tailored for identification of featureless melanoma not diagnosable by clinical examination alone [87]. It’s important to notice that without TBSP the featureless invasive melanoma in patients with multiple clinically dermal nevi could easily go unnoticed at this stage (Figures S21 and S22 are a few examples). The importance of TBM is evident in Figure S23 that shows a symmetric bicomponent dermoscopy (structureless tan and granularity around follicle). Another important clinical observation is the presence of hair in an invasive melanoma, showing that a melanoma can have hair follicles even thick invasive ones.

Yélamos et al. performed RCM examinations to assess lesions that were dermoscopically equivocal. RCMs were read at the same time by the same dermatologist performing the exam [16], the same fashion used during our study. From a total of 60 melanomas, RCM was performed prior to the excision of 22 (36.7%) SPMs (female: 21, 39.6%; male: 1, 14.3%). We used RCM to confirm the need for biopsy, especially for lower limbs clinically suspect nevi. RCM strengthens physician’s diagnostic confidence under real conditions in the clinical setting [16]. Differentiation of NAM and DNM by means of dermoscopy has been proved to be very difficult, yet by RCM remnants of a preexisting nevus can sometimes be seen [5,29,59,65]. The well-known features of melanoma in RCM such as pagetoid cells as bright (reflective) roundish and dendritic within the epidermis, epidermal hyporeflective pagetoid cells (sometimes forming nests), non-edged papillae, atypical cells at the dermoepidermal junction (DEJ), atypical juncional nesting, dermal nests (dense/sparse and cerebriform), and presence of atypical cells infiltrating the papillary dermis [26,27,29,59] were used to suggest malignancy when one or more of these features were present. The lesion architecture under RCM was assessed for presence of abrupt transition, localized distribution of atypical cells at DEJ, and presence of compact dense dermal nests [29]. In our study, the relationship between diagnosis and RCM wasn’t statistically significant, nevertheless it was performed for 18 (36%) in-situ SPMs and 4 (40%) invasive SPMs, being suggestive of malignancy in 15 (83.3%) in-situ SPMs and 3 (75%) invasive SPMs. At bedside examination the female had 85.7% of RCMs suggestive of malignancy, while the father had none. Yet the father’s RCM “negative” lesion was sent immediately for excision for being a new and palpable lesion (Figure S23). Table 4 shows a significant correlation between follow-up and RCM performed, proving it was a valuable tool that helped shorten nevus follow-up time for melanoma diagnosis to a mean of 10 months (SD: 12.2 months).

Melanomas can be sporadic or hereditary [88]. High penetrance genes associated with familial melanoma are *CDKN2A* and *CDK4*. Pathogenic variants in *CDKN2A* correspond to 10% of families with two cases of melanoma and 30 to 40% of families with 3 or more cases, showing association with pancreatic cancer. The prevalence of *CDK4* variant varies from 1 to 3%. Other pathogenic variants have been identified in new high penetrance genes, such as *BAP1*, *POT1*, *ACD* genes, *TERF2IP* and *TERT*. They may be involved in different susceptibility pathways to melanoma [89,90,91]. Nonetheless, the majority (>70%) of familial melanoma cases are of unknown genetic etiology [41,92].

According to “next generation sequencing”, our female patient carries two variants of undetermined clinical significance (VUS), as follows: (1) *MEN1*, chr11: 64.577.500 NM_000244.3: c.82G>C: p.(Gly28Arg)–heterozygosity (52.04%). The gene MEN1 is related to Multiple Endocrine Neoplasia Syndrome type 1, of autosomal dominant inheritance (OMIM:613733). Multiple Endocrine Neoplasia Syndrome type 1 is characterized by an elevated risk for endocrine tumors of the parathyroid, pituitary, gastric and biliopancreatic tract [93,94,95]. (2) *MSH3*, chr5: 79.952.279 NM_002439.5: c.287C>T: p.(Pro96Leu)–heterozygosity (48.14%). The *MSH3* gene is associated with familial adenomatous polyposis type 4, of autosomal recessive inheritance (OMIM:617100). Biallelic pathogenic variants in *MSH3* gene are associated with the development of multiple colonic adenomas in adulthood, which often progress to colorectal cancer. Proliferative lesions in other tissues may occur [95,96,97].

Variants in genes of low to moderate penetrance related to skin pigmentation and differentiation of melanocytes, such as the *MC1R* and *MITF* genes, are also of great importance in determining the risk for cutaneous melanoma [41,90,98]. The *MC1R* gene is highly polymorphic, more than 200 variant alleles have been described [41,98]. The highest levels of pheomelanin are associated with the “red hair color” (RHC) phenotype (i.e., fair skin, red hair, ephelides and sun light sensitivity) and it is caused by specific polymorphisms in the *MC1R* locus [9]. Susceptibility variants in the *MC1R* gene are classified as RHC (R) and non-RHC (r). Genetic variants in the *MC1R* gene are the most prevalent melanoma genetic risk factor in the white population. Still, 20 to 40% of CM cases occur in individuals with wildtype *MC1R* [74]. Both R and r alleles are associated with melanoma and have similar population attributable melanoma risk, independently of their effect on pigmentation, as previously suggested [9,74,98,99].

Both studied patients have two R heterozygotic variants of *MC1R*, c.425G>A: p.(Arg142His) and c.451C>T: p.(Arg151Cys), conferring them not only melanoma risk, but also increased risk for BCC and SCC due to the R151C variant. Furthermore, the R142H variant seems to correlate with blue/green eyes [9]. The risk of developing melanoma appears to be higher as the number of *MC1R* variants increase in the carrier; it doubles in subjects with a single variant and can go as high as six times in an individual with two or more variants [9,99]. The father has a high count of moles, yet the daughter has an impressive number of nevi (>450). Duffy et.al. have shown that having more than 20 large naevi (≥5 mm diameter) associated with *MC1R* R/R genotype increase up to 25-fold the melanoma risk, compared with people with zero to four nevi and *MC1R* WT/WT genotype [46]. In other studies, no association was found between the *MC1R* variants and high counts of acquired nevi in families with hereditary melanoma [9].

During the 5-year follow-up, they have undergone biochemical and imaging exams without evidence of systemic disease to date. For life, skin examination and TBM has been advised in a periodic fashion. Patients with many large naevi and the RHC phenotype, particularly those with an *MC1R* R/R genotype, have an unusually high risk of melanoma, similar to familial melanoma and should undergo intensive skin surveillance, that may include TBSP and SDDI, both have proved to be useful in the early diagnosis of melanoma [19]. Ongoing correlation between clinical and genetic findings is advisable, as well as multispecialty medical consultations for both patients.

Particularly in young women, it seems that the increased production of female sex hormones can alter the effect of the RHC-*MC1R* variants on the skin phenotype and the risk of melanoma [9]. The female patient noticed a rapid onset of acquired nevi after her teenage years. She had menarche at 13-year-old and has taken “progestogen-only” oral contraceptive (OC) continuously for up to eight years (desogestrel 75 mg given at a constant dose, daily and without a break, from 2010 to 2018). The OC was stopped due to an enlarged uterus and fibroids. Sun Q, et al. conducted a meta-analysis of 38 studies that met quality requirements, involving over 3.5 million participants, and found an increased risk of melanoma in women who had a long-term use of OC (≥5 years) [100]. Other studies don’t show an association between OC use and risk of CM [101]. Epidemiological evidence suggests an association of melanocytic proliferation with hormonal and proinflammatory pathways [74]. Suppression of various immune system components can sway the immune response in favor of, or in opposition to, carcinogenesis, tumor progression, and metastasis. Immunosuppressive drugs may have additional actions on antiproliferative or cancer-promoting pathways independent of the immune system. Evaluation of individual immunosuppressive agents is key for appropriately assessing a patient’s risk [68]. OC use is causally associated with an increased risk of depression in adolescents as well as in adults, especially shortly after the initiation [102]. Depression can lead to systemic immune suppression, which could potentially alter the anti-tumor immune response [103].

Our study has several limitations. It’s a small sized prospective, single-center study of only two high-risk related patients, impairing generalizability to the general population. Nonetheless, combined they had a high initial number of 672 monitored melanocytic nevi. Thru the follow-up period a total of sixty melanomas were diagnosed, allowing plenty of room for analysis and the considerations made. Furthermore, the rarity of more than twenty melanomas in a single individual had to be explored. This is a pilot MPM study, and our results should be interpreted with caution. Notwithstanding, our work may serve as a foundation for future studies with larger sample sizes using hospital and population-based data.

In addition, we hypothesize that there are many unaware carriers of genes that confers increased risk for melanoma, that are clinically not considered high-risk patients, and may be scaping early melanoma detection. Even patients with known pathologic gene variants, for other types of cancers, when undergo a genetic test, won’t be aware if they are carries or not of low/moderate melanoma risk genes, especially highly polymorphic ones, because they won’t be disclosed on test results. These groups of patients could benefit not only from melanoma prevention education and photoprotection, but also from skin and nevi monitoring by TBM. We propose for genetic laboratories to disclosure melanoma risk low/moderate genes, so patients can be advised accordingly. We also suggest for governments, health insurances, and skin cancer task forces to review their positions on total body mapping with TBSP, DD, SDDI, and RCM, when indicated.

## 5. Conclusions

Nevus-like melanomas and amelanotic/hypopigmented melanomas are known to scape clinical detection. This study is an eye opener for the need of strict monitoring using a combination of different, yet synergic, skin imaging technologies, along with patient compliance to TBSE and TBM. Prevention of melanoma is unlikely to be achieved solely by screening, nonetheless it’s an important tool for diagnosis at a surgical treatable stage. Better selection of patients, including those carriers of low/moderate melanoma risk genes, increased access, and referral to dermatology/skin cancer clinics may be parts of the missing links to better mortality rates. Direct or indirect immunosuppressive agents need more in-depth studies and may disclosure another clue for the failure in lowering mortality rates. Furthermore, an increased awareness of governments and health care insurances is necessary, along with an urgency for inclusion and reimbursement of new melanoma diagnosis technologies, allowing more patients a chance to earlier diagnosis and longer survivals.

## Figures and Tables

**Table 1:** Exhibits the treatment ratio of benign excised lesions for each skin cancer diagnosed: number-needed-to-treat (NNT), the ratio of moles excised for each melanoma diagnosed: number-needed-to-excise (NNE), and the proportion of in-situ/invasive (<1 and >1 mm Breslow thickness combined) melanomas diagnosed per patient: in-situ/invasive ratio (IIR).

	Benign × Malignant Treatment Ratio			Nevi × Melanoma Excision Ratio			In-Situ/Invasive Ratio	
	Benign	Malignant	NNT	Nevi	Melanoma	NNE	In-Situ CM (%)	Invasive CM (%)
Female	92	53	1.7	92	53	1.7	84.9	15.1
Male	51	18	2.8	48	7	6.9	71.4	28.6
Total	143	71	2.0	140	60	2.3		

**Table 2:** Genetic profile of two multiple primary melanoma patients.

Patient	Age at First Melanoma	Age at Last Melanoma	Skin Phototype, RHC Phenotype *	Atypical Mole Syndrome Phenotype (>150 Nevi)	*MC1R* Variants	*MC1R* Risk Classification **	VUS ***	Number of Primary Melanomas	1st or 2nd Degree Relatives with Melanoma
Male	61	65	I; yes	yes	R151C; R142H	R; R	-	7	2
Female	32	37	I; yes	yes	R151C; R142H	R; R	*MEN1* *MSH3*	53	2

* Red hair color (RHC); ** R: variants associated with red hair color and more than 2× increased risk for melanoma; r: variants not associated with red hair color and 1–2× increased risk for melanoma [9,41]. *** VUS: variants of undetermined clinical significance.

**Table 3:** Descriptive analysis of excised melanomas.

Variables	Female (*N* = 1)	Male (*N* = 1)	All Patients (*N* = 2)
*Time of Sddi Follow-Up to Suspect Melanoma* *(Months between first and last digital dermoscopy)*			
Mean (Standard deviation)	19.1 (15.8)	22.5 (23.4)	19.4 (16.6)
Median (25th percentile–75th percentile)	14 (4–35.5)	20.6 (1.7–46.1)	14 (2.3–35.7)
Minimum–Maximum	0–45.9	0–46.1	0–46.1
Total number of melanomas	52	6	58
*Time of Last Sddi to Excisional Biopsy (Months)*			
Mean (Standard deviation)	1.2 (1.2)	1.1 (0.5)	1.1 (1.1)
Median (25th percentile–75th percentile)	1 (0.6–1.2)	1.3 (0.7–1.4)	1 (0.6–1.2)
Minimum–Maximum	0.1–7.4	0.4–1.5	0.1–7.4
Total number of melanomas	50	6	58
*Age at Excision*			
Mean (Standard deviation)	34.1 (1.5)	63 (1.7)	37.5 (9.5)
Median (25th percentile–75th percentile)	34 (33–35)	63 (61–65)	35 (33–36)
Minimum–Maximum	32–37	61–65	32–65
Total number of melanomas	53	7	60
*Anatomical Location*			
Acral	1 (1.9%)	0 (0%)	1 (1.7%)
Head & neck	1 (1.9%)	0 (0%)	1 (1.7%)
Lower limbs	24 (45.3%)	3 (42.9%)	27 (45%)
Perineal	1 (1.9%)	0 (0%)	1 (1.7%)
Trunk dorsal	9 (17%)	1 (14.3%)	10 (16.7%)
Trunk ventral	9 (17%)	1 (14.3%)	10 (16.7%)
Upper limbs	8 (15.1%)	2 (28.6%)	10 (16.7%)
Total number of melanomas	53 (100%)	7 (100%)	60 (100%)
*Body Side (Laterality)*			
Left	36 (67.9%)	3 (42.9%)	39 (65%)
Midline	2 (3.8%)	0 (0%)	2 (3.3%)
Right	15 (28.3%)	4 (57.1%)	19 (31.7%)
Total number of melanomas	53 (100%)	7 (100%)	60 (100%)
*Clinical Pattern*			
Amelanotic/Nonmelanoma skin cancer-like	5 (9.4%)	1 (14.3%)	6 (10%)
Nevus-like	44 (83%)	5 (71.4%)	49 (81.7%)
Typical melanoma	3 (5.7%)	0 (0%)	3 (5%)
Unknown	1 (1.9%)	1 (14.3%)	2 (3.3%)
Total number of melanomas	53 (100%)	7 (100%)	60 (100%)
*Clinical Pigmentation*			
Missing	1	1	2
Amelanotic (pink lesion)	13 (25%)	0 (0%)	13 (22.4%)
Hyperpigmented (dark brown/black lesion)	3 (5.8%)	0 (0%)	3 (5.2%)
Hypopigmented (tan lesion)	21 (40.4%)	5 (83.3%)	26 (44.8%)
Pigmented (brown lesion)	15 (28.8%)	1 (16.7%)	16 (27.6%)
Total number of melanomas	52 (100%)	6 (100%)	58 (100%)
*Palpable Lesion (Palpability)*			
Missing	0	1	1
No	32 (60.4%)	5 (83.3%)	37 (62.7%)
Yes	21 (39.6%)	1 (16.7%)	22 (37.3%)
Total number of melanomas	53 (100%)	6 (100%)	59 (100%)
*Borders*			
Missing	1	1	2
Ill-defined	33 (63.5%)	6 (100%)	39 (67.2%)
Not defined	1 (1.9%)	0 (0%)	1 (1.7%)
Well-defined	18 (34.6%)	0 (0%)	18 (31%)
Total number of melanomas	52 (100%)	6 (100%)	58 (100%)
*Global Dermoscopic Pattern*			
Asymmetric multiple component	34 (64.2%)	1 (14.3%)	35 (58.3%)
Asymmetric bicomponent	2 (3.8%)	1 (14.3%)	3 (5%)
Homogeneous	6 (11.3%)	1 (14.3%)	7 (11.7%)
Symmetric multiple component	4 (7.5%)	3 (42.8%)	7 (11.7%)
Symmetric bicomponent	5 (9.4%)	0 (0%)	5 (8.3%)
Starburst	1 (1.9%)	0 (0%)	1 (1.7%)
Unknown	1 (1.9%)	1 (14.3%)	2 (3.3%)
Total number of melanomas	53 (100%)	7 (100%)	60 (100%)
*Number of Melanoma Specific Structures per Lesion*			
Mean (Standard deviation)	4 (1.3)	3.5 (0.5)	3.9 (1.2)
Median (25th percentile–75th percentile)	4 (3–5)	3.5 (3–4)	4 (3–5)
Minimum–Maximum	1–6	3–4	1–6
Total number of melanomas	52	6	58
*RCM Performed*			
NO	32 (60.4%)	6 (85.7%)	38 (63.3%)
YES	21 (39.6%)	1 (14.3%)	22 (36.7%)
Total number of melanomas	53 (100%)	7 (100%)	60 (100%)
*RCM Suggestive of Malignancy*			
Missing	32	6	38
NO	3 (14.3%)	1 (100%)	4 (18.2%)
YES	18 (85.7%)	0 (0%)	18 (81.8%)
Total number of melanomas	21 (100%)	1 (100%)	22 (100%)
*Lesion Size on Pathology Report (mm)*			
Mean (Standard deviation)	5.9 (2)	6.7 (4.3)	6 (2.4)
Median (25th percentile–75th percentile)	5 (4.5–7)	7 (3–8)	5 (4–7)
Minimum–Maximum	3–12	3–15	3–15
Total number of melanomas	52	7	59
*Pathologic Diagnosis*			
Melanoma in-situ	45 (84.9%)	5 (71.4%)	50 (83.3%)
Invasive melanoma < 1 mm	8 (15.1%)	1 (14.3%)	9 (15%)
Invasive melanoma > 1 mm	0 (0%)	1 (14.3%)	1 (1.7%)
Total number of melanomas	53 (100%)	7 (100%)	60 (100%)
*Histology Type*			
Lentigo Maligna	1 (1.9%)	0 (0%)	1 (1.7%)
Lentiginous Melanoma	0 (0%)	1 (14.3%)	1 (1.7%)
Superficial Spreading Melanoma	52 (98.1%)	6 (85.7%)	58 (96.6%)
Total number of melanomas	53 (100%)	7 (100%)	60 (100%)
*Nevus-Associated Melanoma*			
No, “de novo”	10 (18.9%)	4 (57.1%)	14 (23.3%)
Yes, juncional nevus	4 (7.5%)	0 (0%)	4 (6.7%)
Yes, compound nevus	19 (35.8%)	1 (14.3%)	20 (33.3%)
Yes, dermal nevus	17 (32.1%)	1 (14.3%)	18 (30%)
Yes, dysplastic nevus	3 (5.7%)	1 (14.3%)	4 (6.7%)
Total number of melanomas	53 (100%)	7 (100%)	60 (100%)
*Histological Regression*			
No	49 (92.5%)	7 (100%)	56 (93.3%)
Yes	4 (7.5%)	0 (0%)	4 (6.7%)
Total number of melanomas	53 (100%)	7 (100%)	60 (100%)
*Intratumoral Inflamatory Infiltrate*			
No	9 (17%)	3 (42.9%)	12 (20%)
Yes	44 (83%)	4 (57.1%)	48 (80%)
Total number of melanomas	53 (100%)	7 (100%)	60 (100%)
*Margins After Biopsy*			
Free	49 (92.4%)	7 (100%)	56 (93.4%)
Compromised	1 (1.8%)	0	1 (1.6%)
Coincident	3 (5.8%)	0	3 (5%)
Total number of melanomas	53 (100%)	7 (100%)	60 (100%)
*Wide Local Excision*			
No	44 (83%)	4 (57.1%)	48 (80%)
Yes	9 (17%)	3 (42.9%)	12 (20%)
Total number of melanomas	53 (100%)	7 (100%)	60 (100%)
*Indication For Slnb*			
NO	8 (100%)	1 (50%)	9 (90%)
YES	0 (0%)	1 (50%)	1 (10%)
Total number of melanomas	8 (100%)	2 (100%)	10 (100%)

**Table 4:** Time correlation (in months) between the first to the last dermoscopy and RCM performed of 58 SPMs.

Time of SDDI Follow-Up by RCM Performed ^1^	No	Yes	*p*-Value
Mean (Standard deviation)	25.2 (16.4)	10 (12.2)	0.002 ^##^
Median (25th percentile–75th percentile)	29 (12.2–40.2)	6.3 (0.8–14)	
Minimum–Maximum	0–46.1	0–35.7	
Total number of melanomas	36	22	

^1^ PMs were excluded from the analyzes. ^##^ non-parametric Mann-Whitney test.

**Table 5:** Time correlation (in months) between the first to the last dermoscopy and nevus-associated melanoma (NAM) of 58 SPMs.

Time of SDDI Follow-Up by NAM ^1^	No, “De Novo”	Yes, Compound NEVUS	Yes, Dermal NEVUS	Yes, Dysplastic NEVUS	Yes, Juncional NEVUS	*p*-Value
Mean (Standard deviation)	17.1 (19.1)	14.2 (13.6)	23.9 (16)	41.7 (3)	11.7 (13)	0.016 ^#^
Median (25th percentile–75th percentile)	6.6 (1.7–35.7)	13.1 (0.8–25.6)	29.8 (12.2–36.1)	40.2 (40.2–43.1)	11.9 (0.4–22.9)	
Minimum–Maximum	0–45.9	0–35.7	0–46.1	40.2–46.1	0–22.9	
Total number of melanomas	13	20	17	4	4	

^1^ PMs were excluded from the analyzes. ^#^ non-parametric Kruskal-Wallis test.

**Table 6:** Time correlation (in months) between the first to the last dermoscopy and intratumoral inflammatory infiltrate of 58 SPMs.

Time of SDDI Follow-Up by Intratumoral Inflammatory Infiltrate ^1^	No	Yes	*p*-Value
Mean (Standard deviation)	37 (9.7)	14.8 (14.9)	<0.001 ^##^
Median (25th percentile–75th percentile)	40.2 (29.2–43.9)	9.5 (1.4–31)	
Minimum–Maximum	19.4–46.1	0–45.9	
Total number of melanomas	12	46	

^1^ PMs were excluded from the analyzes. ^##^ non-parametric Mann-Whitney test.

**Table 7:** Relationship between the pathologic diagnosis with the variables clinical pigmentation and NAM.

Pathologic Diagnosis ^1^ (Invasive Melanoma × In-Situ Melanoma)	OR IC (95%)	*p*-Value
*Clinical Pigmentation*		0.946
hyperpigmented × amelanotic	0 (0; -)	0.999
hypopigmented × amelanotic	0.46 (0.04; 5.65)	0.541
pigmented × amelanotic	0 (0; -)	0.998
*Nevus-Associated Melanoma*		0.023
yes, common nevi (juncional, compound and dermal) × no, “de novo”	0.04 (0; 0.39)	0.006
yes, dysplastic nevi × no, “de novo”	0.72 (0.03; 15.2)	0.830

^1^ Multivariate logistic regression model.

## Data Availability

Data supporting the reported results can be found in the tables, figures, and supplements within the text and Supplementary Materials.

## Supplementary Materials

The following supporting information can be downloaded at: https://www.mdpi.com/article/10.3390/life13102102/s1, tables from S1 to S22 and figures from S1 to S23.

## Abbreviations

MPM: multiple primary melanoma, SPM: second primary melanoma, PM: primary melanoma, NMSC: non-melanoma skin cancer, SK: seborrheic keratosis, RCM: reflectance confocal microscopy, NNT: number-needed to treat, NNE: number-needed to excise, IIR: in-situ/invasive melanoma ratio, PNNE: patient number-needed to excise, TBSE: total body skin exam, TBM: total body mapping, TBSP: total body skin photography, DD: digital dermoscopy, SDDI: sequential digital dermoscopy imaging, CM: cutaneous melanoma, NAM: nevus-associated melanoma, DNM: “de novo” melanoma, DEJ: dermoepidermal junction, RHC: “red hair color”, OC: oral contraceptive.
