# Supplementary material for: Multiple Primary Melanoma: A Five-Year Prospective Single-Center Follow-Up Study of Two MC1R R/R Genotype Carriers
Source: Life (Basel). 2023 Oct 23;13(10):2102. doi: 10.3390/life13102102 (PMC10608495; doi:10.3390/life13102102)
Supplement: Supplementary file 1 [file life-13-02102-s001.zip › life-2583678-supplementary.pdf]

life

# e-Supplement

**Table S1.** Demographic characteristics of the two studied patients.

| Variables | Daughter (N=1) | Father (N=1) | All Patients (N=2) |
|---|---|---|---|
| *SEX* | | | |
| Female | 1 | 0 | 1 |
| Male | 0 | 1 | 1 |
| *YEAR OF FIRST MELANOMA DIAGNOSIS* | | | |
| 2018 | 1 | 1 | 2 |
| *AGE AT BASELINE* | | | |
| 32 | 1 | 0 | 1 |
| 61 | 0 | 1 | 1 |
| Mean (Standard deviation) | | | 46.5 (20.5) |
| *AGE AT END OF THE STUDY* | | | |
| 37 | 1 | 0 | 1 |
| 65 | 0 | 1 | 1 |
| Mean (Standard deviation) | | | 51 (19.8) |
| *PHENOTYPE* | | | |
| I | 1 | 1 | 2 |
| *EYE COLOR* | | | |
| Green/hazel | 1 | 1 | 2 |
| *HAIR COLOR* | | | |
| Red | 1 | 1 | 2 |
| *TOTAL NEVI* | | | |
| > 150 | 1 | 1 | 2 |
| *FAMILY HISTORY OF MELANOMA* | | | |
| Yes | 1 | 1 | 2 |
| *NEVI REGISTERED ON BASELINE SDDI* | | | |
| 455 | 1 | 0 | 1 |
| 172 | 0 | 1 | 1 |
| Mean (Standard deviation) | | | 313.5 (200.1) |
| *POSITIONS REGISTERED ON FINAL SDDI* | | | |
| 689 | 1 | 0 | 1 |
| 313 | 0 | 1 | 1 |
| Mean (Standard deviation) | | | 501 (265.9) |
| *FOLLOW-UP IN MONTHS* | | | |
| 56 | 1 | 0 | 1 |
| 51 | 0 | 1 | 1 |
| Mean (Standard deviation) | | | 53.5 (3.5) |
| *EXCISED LESIONS FOR THE PERIOD* | | | |

| | | | |
|---|---|---|---|
| 145 | 1 | 0 | 1 |
| 69 | 0 | 1 | 1 |
| Mean (Standard deviation) | | | 107 (53.7) |
| ***EXCISED NEVI FOR THE PERIOD*** | | | |
| 92 | 1 | 0 | 1 |
| 48 | 0 | 1 | 1 |
| Mean (Standard deviation) | | | 70 (31.1) |
| ***EXCISED MELANOMAS FOR THE PERIOD*** | | | |
| 53 | 1 | 0 | 1 |
| 7 | 0 | 1 | 1 |
| Mean (Standard deviation) | | | 30 (32.5) |
| ***EXCISED BCCs FOR THE PERIOD*** | | | |
| 0 | 1 | 0 | 1 |
| 7 | 0 | 1 | 1 |
| Mean (Standard deviation) | | | 3.5 (4.9) |
| ***EXCISED SCCs FOR THE PERIOD*** | | | |
| 0 | 1 | 0 | 1 |
| 2 | 0 | 1 | 1 |
| Mean (Standard deviation) | | | 1 (1.4) |
| ***EXCISED AKs FOR THE PERIOD*** | | | |
| 0 | 1 | 0 | 1 |
| 2 | 0 | 1 | 1 |
| Mean (Standard deviation) | | | 1 (1.4) |
| ***EXCISED SKs FOR THE PERIOD*** | | | |
| 0 | 1 | 0 | 1 |
| 2 | 0 | 1 | 1 |
| Mean (Standard deviation) | | | 1 (1.4) |
| ***EXCISED HEMANGIOMA FOR THE PERIOD*** | | | |
| 0 | 1 | 0 | 1 |
| 1 | 0 | 1 | 1 |
| Mean (Standard deviation) | | | 0.5 (0.7) |

**Table S2.** Trend/cumulative risk of new SPM for father and daughter relative to number of cutaneous melanomas (CM) per semester. Table A2 presents the accumulated number of CM in 5 years (60 months) for father, daughter, and both.

| TIME IN MONTHS | CUMULATIVE No. CM (FEMALE) | CUMULATIVE No. CM (MALE) | CUMULATIVE No. CM (TOTAL) |
|---|---|---|---|
| Month 0 | 1 | 1 | 2 |
| Up to month 6 | 9 | 2 | 11 |
| Up to month 12 | 14 | 2 | 16 |
| Up to month 18 | 23 | 3 | 26 |
| Up to month 24 | 23 | 3 | 26 |
| Up to month 30 | 32 | 4 | 36 |
| Up to month 36 | 36 | 4 | 40 |
| Up to month 42 | 45 | 5 | 50 |
| Up to month 48 | 51 | 5 | 56 |
| Up to month 54 | 51 | 7 | 58 |
| Up to month 60 | 53 | 7 | 60 |

[1] Based on these data, some models were adjusted to estimate the cumulative number of melanomas as a function of time (linear model, quadratic model, and cubic model).

**Table S3.** Time correlation (in months) between the first to the last dermoscopy and melanoma clinical pattern of 58 SPM.

| TIME OF SSDI FOLLOW-UP BY CLINICAL PATTERN[1] | AMELANOTIC/ NONMELANOMA SKIN CANCER-LIKE | NEVUS-LIKE | TYPICAL MELANOMA | p-value |
|---|---|---|---|---|
| Mean (Standard deviation) | 17.5 (17.9) | 19.9 (16.3) | 14.9 (25.1) | 0.633[#] |
| Median (25th percentile – 75th percentile) | 16 (1.6 – 35.5) | 14 (4.5 – 35.7) | 0.8 (0 – 43.9) | |
| Minimum - Maximum | 0 – 35.7 | 0 – 46.1 | 0 – 43.9 | |
| Total number of melanomas | 6 | 49 | 3 | |

[1]PMs were excluded from the analyzes. [#]non-parametric Kruskal-Wallis test.

**Table S4.** Time correlation (in months) between the first to the last dermoscopy and global dermatoscopic pattern of 58 SPM.

| TIME OF SSDI FOLLOW-UP BY GLOBAL DERMOSCOPIC PATTERN[1] | ASYMMETRIC MULTIPLE COMPONENTS | ASYMMETRIC BICOMPONENT | HOMOGENEOUS | SYMMETRIC MULTIPLE COMPONENTS[2] | SYMMETRIC BICOMPONENT | p-value |
|---|---|---|---|---|---|---|
| Mean (Standard deviation) | 19.5 (15.2) | 21.7 (21.3) | 13.6 (15.8) | 24.5 (19.5) | 17.5 (24) | 0.632[#] |
| Median (25th percentile – 75th percentile) | 19.4 (4.5 - 31) | 12.2 (6.9 – 46.1) | 6 (0 – 35.5) | 35.6 (1.9 – 37.5) | 0 (0 – 43.9) | |
| Minimum – Maximum | 0 – 45.9 | 6.9 – 46.1 | 0 – 35.7 | 0 – 46.1 | 0 – 43.9 | |
| Total number of melanomas | 35 | 3 | 7 | 8 | 5 | |

[1]PMs were excluded from the analyzes. [2]The starburst pattern lesion was included as symmetric multiple components for the analyzes. [#]non-parametric Kruskal-Wallis test.

**Table S5.** Time correlation (in months) between the first to the last dermoscopy and pigmentation pattern of 58 SPM.

| TIME OF SSDI FOLLOW-UP BY PIGMENTATION PATTERN[1] | AMELANOTIC | HYPERPIGMENTED | HYPOPIGMENTED | PIGMENTED | p-value |
|---|---|---|---|---|---|
| Mean (Standard deviation) | 19.4 (16.4) | 4.9 (7.9) | 21.8 (17.8) | 18.3 (15.5) | 0.338[#] |
| Median (25th percentile – 75th percentile) | 14 (1.6 – 35.5) | 0.8 (0 - 14) | 16.7 (4.1 – 40.2) | 22.1 (2.9 – 29.6) | |
| Minimum - Maximum | 0 – 43.9 | 0 - 14 | 0 – 46.1 | 0 – 46.1 | |
| Total number of melanomas | 13 | 3 | 26 | 16 | |

[1]PMs were excluded from the analyzes. [#]non-parametric Kruskal-Wallis test.

**Table S6.** Time correlation (in months) between the first to the last dermoscopy and palpable lesion of 58 SPM.

| TIME OF SSDI FOLLOW-UP BY PALPABLE LESION[1] | NO | YES | p-value |
|---|---|---|---|
| Mean (Standard deviation) | 16.8 (16.4) | 24.1 (16.2) | 0.110[##] |
| Median (25th percentile – 75th percentile) | 12.2 (1.4 – 29.8) | 31 (12.2 – 36.1) | |
| Minimum - Maximum | 0 – 46.1 | 0 – 43.9 | |
| Total number of melanomas | 37 | 21 | |

[1]PMs were excluded from the analyzes. [##]non-parametric Mann-Whitney test.

**Table S7.** Time correlation (in months) between the first to the last dermoscopy and borders of 58 SPM.

| TIME OF SSDI FOLLOW-UP BY BORDERS[1] | ILL-DEFINED OR NOT-DIFINED | WELL-DEFINED | p-value |
|---|---|---|---|
| Mean (Standard deviation) | 21.5 (17.7) | 14.8 (13.1) | 0.270[##] |
| Median (25th percentile – 75th percentile) | 22.1 (1.5 – 39.5) | 9.5 (4.5 – 28.3) | |
| Minimum - Maximum | 0 – 46.1 | 0 – 35.7 | |
| Total number of melanomas | 40 | 18 | |

[1]PMs were excluded from the analyzes. [##]non-parametric Mann-Whitney test.

**Table S8.** Time correlation (in months) between the first to the last dermoscopy and RCM suggestive of malignancy of 22 SPM.

| TIME OF SSDI FOLLOW-UP BY MCR SUGGESTIVE OF MALIGNANCY[1] | NO | YES | p-value |
|---|---|---|---|
| Mean (Standard deviation) | 4.2 (3.1) | 11.3 (13.1) | 0.764[#] |
| Median (25th percentile – 75th percentile) | 4.6 (1.6 – 6.9) | 6.3 (0.8 - 14) | |
| Minimum - Maximum | 0.8 – 6.9 | 0 – 35.7 | |
| Total number of melanomas | 4 | 18 | |

[1]PMs and SPM that didn't undergo RCM prior to excision were excluded from the analyzes. [#]non-parametric Kruskal-Wallis test.

**Table S9.** Time correlation (in months) between the first to the last dermoscopy and size on pathology report (mm) of 57 SPM.

| TIME OF SSDI FOLLOW-UP BY SIZE (MM)[1] | TIME OF SSDI FOLLOW-UP (Months between first and last dermoscopy) | SIZE ON PATHOLOGY REPORT (MM) | COEFFICIENT | p-value |
|---|---|---|---|---|
| Mean (Standard deviation) | 19.8 (16.5) | 5.8 (2.1) | -0.162 | 0.228[*] |
| Median (25th percentile – 75th percentile) | 14 (3.5 – 35.7) | 5 (4 - 7) | | |
| Minimum - Maximum | 0 – 46.1 | 3 - 12 | | |
| Total number of melanomas | 57 | 57 | | |

[1]PMs and one unknown were excluded from the analyzes. [*]Spearman correlation coefficient.

**Table S10.** Time correlation (in months) between the first to the last dermoscopy and diagnosis of 58 SPM.

| TIME OF SSDI FOLLOW-UP BY DIAGNOSIS[1] | *IN-SITU* MELANOMA | INVASIVE MELANOMA (<1MM OR >1MM) | p-value |
|---|---|---|---|
| Mean (Standard deviation) | 19.6 (16.3) | 18.2 (18.9) | 0.731## |
| Median (25th percentile – 75th percentile) | 14 (3.5 – 35.5) | 6.9 (2.3 – 35.7) | |
| Minimum - Maximum | 0 – 46.1 | 0 – 43.9 | |
| Total number of melanomas | 49 | 9 | |

[1]PMs were excluded from the analyzes. ##non-parametric Mann-Whitney test.

**Table S11.** Time correlation (in months) between the first to the last dermoscopy and histological regression of 58 SPM.

| TIME OF SSDI FOLLOW-UP BY HISTOLOGICAL REGRESSION[1] | NO | YES | p-value |
|---|---|---|---|
| Mean (Standard deviation) | 19.5 (16.7) | 18.4 (1.5) | 0.866## |
| Median (25th percentile – 75th percentile) | 14 (2.3 – 35.7) | 18.5 (3.4 – 33.4) | |
| Minimum - Maximum | 0 – 46.1 | 0.8 – 35.7 | |
| Total number of melanomas | 54 | 4 | |

[1]PMs were excluded from the analyzes. ##non-parametric Mann-Whitney test.

**Table S12.** Correlation between clinical pattern and diagnosis of 58 SPM.

| CLINICAL PATTERN BY DIAGNOSIS[1] | *IN-SITU* MELANOMA | INVASIVE MELANOMA (<1MM OR >1MM) | Total | p-value |
|---|---|---|---|---|
| Amelanotic/nonmelanoma skin cancer-like | 4 (8.2%) | 2 (22.2%) | 6 (10.3%) | 0.327 ** |
| Nevus-like | 42 (85.7%) | 7 (77.8%) | 49 (84.5%) | |
| Typical melanoma | 3 (6.1%) | 0 (0%) | 3 (5.2%) | |
| Total | 49 (100%) | 9 (100%) | 58 (100%) | |

[1]PMs were excluded from the analyzes. ** likelihood ratio test.

**Table S13.** Correlation between global dermoscopic pattern and diagnosis of 58 SPM.

| GLOBAL DERMOSCOPIC PATTERN BY DIAGNOSIS[1] | *IN-SITU* MELANOMA | INVASIVE MELANOMA (<1MM OR >1MM) | Total | p-value |
|---|---|---|---|---|
| Asymmetric multiple components | 31 (63.3%) | 4 (44.4%) | 35 (60.3%) | 0.399** |
| Asymmetric bicomponent | 3 (6.1%) | 0 (0%) | 3 (5.2%) | |
| Homogeneous | 5 (10.2%) | 2 (22.2%) | 7 (12.1%) | |
| Symmetric multiple components or starburst | 7 (14.3%) | 1 (11.1%) | 8 (13.8%) | |
| Symmetric bicomponent | 3 (6.1%) | 2 (22.2%) | 5 (8.6%) | |
| Total | 49 (100%) | 9 (100%) | 58 (100%) | |

[1]PMs were excluded from the analyzes. ** likelihood ratio test.

**Table S14.** Correlation between location and diagnosis of 60 MPMs.

| LOCATION BY DIAGNOSIS | *IN-SITU* MELANOMA | INVASIVE MELANOMA (<1MM OR >1MM) | Total | p-value |
|---|---|---|---|---|
| Lower limbs | 23 (46%) | 4 (40%) | 27 (45%) | 0.898** |
| Trunk dorsal | 9 (18%) | 1 (10%) | 10 (16.7%) | |
| Trunk ventral | 8 (16%) | 2 (20%) | 10 (16.7%) | |
| Upper limbs | 8 (16%) | 2 (20%) | 10 (16.7%) | |
| Acral or head & neck or perineal | 2 (4%) | 1 (10%) | 3 (5%) | |
| Total | 50 (100%) | 10 (100%) | 60 (100%) | |

** likelihood ratio test.

**Table S15.** Correlation between location and laterality of 58 SPM.

| LOCATION BY LATERALITY[1] | LEFT | RIGHT | Total | p-value |
|---|---|---|---|---|
| Lower limbs | 20 (51.3%) | 7 (36.8%) | 27 (46.6%) | 0.186** |
| Trunk dorsal | 4 (10.3%) | 6 (31.6%) | 10 (17.2%) | |
| Trunk ventral | 6 (15.4%) | 2 (10.5%) | 8 (13.8%) | |
| Upper limbs | 8 (20.5%) | 2 (10.5%) | 10 (17.2%) | |
| Acral or head & neck or perineal | 1 (2.6%) | 2 (10.5%) | 3 (5.2%) | |
| Total | 39 (100%) | 19 (100%) | 58 (100%) | |

[1]SPM located on midline were excluded from the analyzes. ** likelihood ratio test.

**Table S16.** Time correlation (in months) between the last dermoscopy to the excisional biopsy and location of 58 SPM.

| TIME OF LAST DERMOSCOPY TO EXCISIONAL BIOPSY BY LOCATION[1] | LOWER LIMBS | TRUNK DORSAL | TRUNK VENTRAL | UPPER LIMBS | ACRAL OR HEAD & NECK OR PERINEAL | p-value |
|---|---|---|---|---|---|---|
| Mean (Standard deviation) | 1 (0.5) | 1.1 (1.1) | 1.6 (2.1) | 1.3 (1.3) | 0.7 (0.3) | 0.783# |
| Median (25th percentile – 75th percentile) | 1 (0.6 – 1.4) | 0.6 (0.4 – 1.1) | 1 (0.9 – 1.2) | 0.9 (0.4 – 1.5) | 0.6 (0.4 - 1) | |
| Minimum - Maximum | 0.1 – 1.6 | 0.3 – 3.6 | 0.3 – 7.4 | 0.4 – 4.5 | 0.4 - 1 | |
| Total number of melanomas | 26 | 10 | 10 | 9 | 3 | |

[1]PMs were excluded from the analyzes. #non-parametric Kruskal-Wallis test.

**Table S17.** Time correlation (in months) between the last dermoscopy to the excisional biopsy and pigmentation of 58 SPM.

| TIME OF LAST DERMOSCOPY TO EXCISIONAL BIOPSY BY PIGMENTATION[1] | AMELANOTIC | HYPERPIGMENTED | HYPOPIGMENTED | PIGMENTED | p-value |
|---|---|---|---|---|---|
| Mean (Standard deviation) | 1 (0.7) | 0.4 (0.1) | 1.1 (0.8) | 1.5 (1.8) | 0.303# |
| Median (25th percentile – 75th percentile) | 1 (0.4 – 1.2) | 0.4 (0.3 – 0.6) | 1 (0.7 – 1.2) | 1 (0.6 – 1.5) | |
| Minimum - Maximum | 0.1 – 2.3 | 0.3 – 0.6 | 0.1 – 4.5 | 0.3 – 7.4 | |
| Total number of melanomas | 13 | 3 | 26 | 16 | |

[1]PMs were excluded from the analyzes. #non-parametric Kruskal-Wallis test.

**Table S18.** Time correlation (in months) between the last dermoscopy to the excisional biopsy and palpable lesion of 58 SPM.

| TIME OF LAST DERMOSCOPY TO EXCISIONAL BIOPSY BY PALPABLE LESION[1] | NO | YES | p-value |
|---|---|---|---|
| Mean (Standard deviation) | 1.1 (1.3) | 1.2 (0.8) | 0.437[#] |
| Median (25th percentile – 75th percentile) | 1 (0.4 – 1.2) | 1 (0.7 – 1.2) | |
| Minimum - Maximum | 0.1 – 7.4 | 0.4 – 3.6 | |
| Total number of melanomas | 37 | 21 | |

[1]PMs were excluded from the analyzes. [#]non-parametric Kruskal-Wallis test.

**Table S19.** Relationship between *in-situ* vs. invasive multiple primary melanomas and the variables: location, laterality, clinical pattern, clinical pigmentation, palpable lesion, borders, global dermoscopic pattern, RCM performed, RCM suggestive of malignancy, associated nevus, size (mm), histological regression, intratumoral inflammatory infiltrate, margins after biopsy, and wide local excision.

| MULTIPLE PRIMARY MELANOMAS | *IN-SITU* MELANOMA | INVASIVE MELANOMA | TOTAL OF MPM | p-value |
|---|---|---|---|---|
| ***LOCATION*** | | | | |
| Special sites (head, neck, acral and perineal) | 2 (4%) | 1 (10%) | 3 (5%) | 0.878*** |
| Trunk | 17 (34%) | 3 (30%) | 20 (33.3%) | |
| Upper limbs | 8 (16%) | 2 (20%) | 10 (16.7%) | |
| Lower limbs | 23 (46%) | 4 (40%) | 27 (45%) | |
| Total | 50 (100%) | 10 (100%) | 60 (100%) | |
| ***LATERALITY*** [1] | | | | |
| Left | 34 (70.8%) | 5 (50%) | 39 (67.2%) | 0.270** |
| Right | 14 (29.2%) | 5 (50%) | 19 (32.8%) | |
| Total | 48 (100%) | 10 (100%) | 58 (100%) | |
| ***CLINICAL PATTERN*** [2] | | | | |
| Typical melanoma | 3 (6.1%) | 0 (0%) | 3 (5.2%) | 0.327*** |
| Nevus-like | 42 (85.7%) | 7 (77.8%) | 49 (84.5%) | |
| Amelanotic/nonmelanoma skin cancer-like | 4 (8.2%) | 2 (22.2%) | 6 (10.3%) | |
| Total | 49 (100%) | 9 (100%) | 58 (100%) | |
| ***CLINICAL PIGMENTATION*** [2] | | | | |
| Amelanotic | 11 (22.4%) | 2 (22.2%) | 13 (22.4%) | **0.035*** ** |
| Hypopigmented | 19 (38.8%) | 7 (77.8%) | 26 (44.8%) | |
| Pigmented | 16 (32.7%) | 0 (0%) | 16 (27.6%) | |
| Hyperpigmented | 3 (6.1%) | 0 (0%) | 3 (5.2%) | |
| Total | 49 (100%) | 9 (100%) | 58 (100%) | |
| ***PALPABLE LESION*** [3] | | | | |
| No | 32 (64%) | 5 (55.6%) | 37 (62.7%) | 0.715** |
| Yes | 18 (36%) | 4 (44.4%) | 22 (37.3%) | |
| Total | 50 (100%) | 9 (100%) | 59 (100%) | |
| ***BORDERS*** [2,4] | | | | |
| Ill-defined | 33 (67.3%) | 6 (75%) | 39 (68.4%) | 1.000** |
| Well-defined | 16 (32.7%) | 2 (25%) | 18 (31.6%) | |
| Total | 49 (100%) | 8 (100%) | 57 (100%) | |
| ***GLOBAL DERMOSCOPIC PATTERN*** [2,5] | | | | |

| | | | | |
|---|---|---|---|---|
| Asymmetric | 34 (70.8%) | 4 (44.4%) | 38 (66.7%) | 0.320*** |
| Homogeneous | 5 (10.4%) | 2 (22.2%) | 7 (12.3%) | |
| Symmetric | 9 (18.8%) | 3 (33.3%) | 12 (21.1%) | |
| Total | 48 (100%) | 9 (100%) | 57 (100%) | |
| ***RCM PERFORMED*** | | | | |
| No | 32 (64%) | 6 (60%) | 38 (63.3%) | 1.000** |
| Yes | 18 (36%) | 4 (40%) | 22 (36.7%) | |
| Total | 50 (100%) | 10 (100%) | 60 (100%) | |
| ***RCM SUGGESTIVE OF MALIGNANCY*** [2,6] | | | | |
| Missing | 32 | 6 | 38 | |
| No | 3 (16.7%) | 1 (25%) | 4 (18.2%) | 1.000** |
| Yes | 15 (83.3%) | 3 (75%) | 18 (81.8%) | |
| Total | 18 (100%) | 4 (100%) | 22 (100%) | |
| ***NEVUS ASSOCIATED MELANOMA*** | | | | |
| No - "*de novo*" | 7 (14%) | 7 (70%) | 14 (23.3%) | **0.001*** ** |
| Yes - common (juncional, compound and dermal) | 40 (80%) | 2 (20%) | 42 (70%) | |
| Yes - dysplastic | 3 (6%) | 1 (10%) | 4 (6.7%) | |
| Total | 50 (100%) | 10 (100%) | 60 (100%) | |
| ***SIZE (mm)*** | | | | |
| ≤ 5 | 25 (50%) | 6 (60%) | 31 (51.7%) | 0.732** |
| ≥ 6 | 25 (50%) | 4 (40%) | 29 (48.3%) | |
| Total | 50 (100%) | 10 (100%) | 60 (100%) | |
| ***HISTOLOGICAL REGRESSION*** | | | | |
| No | 47 (94%) | 9 (90%) | 56 (93.3%) | 0.528** |
| Yes | 3 (6%) | 1 (10%) | 4 (6.7%) | |
| Total | 50 (100%) | 10 (100%) | 60 (100%) | |
| ***INTRATUMORAL INFLAMMATORY INFILTRATE*** | | | | |
| No | 12 (24%) | 0 (0%) | 12 (20%) | 0.188** |
| Yes | 38 (76%) | 10 (100%) | 48 (80%) | |
| Total | 50 (100%) | 10 (100%) | 60 (100%) | |
| ***MARGINS AFTER BIOPSY*** | | | | |
| Free | 46 (92%) | 10 (100%) | 56 (93.3%) | 1.000** |
| Compromised or coincident | 4 (8%) | 0 (0%) | 4 (6.7%) | |
| Total | 50 (100%) | 10 (100%) | 60 (100%) | |
| ***WIDE LOCAL EXCISION*** | | | | |
| No | 41 (82%) | 7 (70%) | 48 (80%) | 0.403** |
| Yes | 9 (18%) | 3 (30%) | 12 (20%) | |
| Total | 50 (100%) | 10 (100%) | 60 (100%) | |

[1]Lesions located in the midline were excluded from the analyzes because they totaled less than 3 patients. [2]PMs were excluded from the analyzes. [3]The male PM was missing the information and was excluded from the analyzes. [4]One lesion was classified as border not defined and was excluded from the analyzes. [5]The starburst pattern was excluded from the analyzes and multicomponent or bicomponent patterns were combined into asymmetric and symmetric. [6]SPM that didn't undergo RCM prior to excision were excluded from the analyzes. *Chi-square test; **Fisher's Exact Test; *** Likelihood ratio test.

**Table S20.** Correlation between father and daughter and the number of melanoma specific dermoscopic structures of SPM.

| NUMBER OF MELANOMA SPECIFIC STRUCTURES PER LESION[1] | FEMALE | MALE | TOTAL | p-valor |
|---|---|---|---|---|
| Mean (Standard deviation) | 4 (1,3) | 3,5 (0,5) | 3,9 (1,2) | 0,305## |
| Median (25th percentile – 75th percentile) | 4 (3 - 5) | 3,5 (3 - 4) | 4 (3 - 5) | |
| Minimum - Maximum | 1 - 6 | 3 - 4 | 1 - 6 | |
| Total number of melanomas | 52 | 6 | 58 | |

[1]PMs were excluded from the analyzes. ##non-parametric Mann-Whitney test.

**Table S21.** Correlation between "de novo" melanoma and tumor anatomical location

| LOCATION BY NEVUS-ASSOCIATED MELANOMA[1] | NO – "DE NOVO" | YES | TOTAL | p-valor |
|---|---|---|---|---|
| Lower limbs | 4 (30,8%) | 23 (52,3%) | 27 (47,4%) | 0,412** |
| Trunk dorsal | 3 (23,1%) | 7 (15,9%) | 10 (17,5%) | |
| Trunk ventral | 4 (30,8%) | 6 (13,6%) | 10 (17,5%) | |
| Upper limbs | 2 (15,4%) | 8 (18,2%) | 10 (17,5%) | |
| Total | 13 (100%) | 44 (100%) | 57 (100%) | |

[1]Acral, head & neck, and perineal SPM were excluded from comparisons because there was only tumor per site. ** likelihood ratio test.

**Table S22.** Correlation between "*de novo*" melanoma and absence of histological regression of SPM.

| HISTOLOGICAL REGRESSION BY NEVUS-ASSOCIATED MELANOMA | NO, "*DE NOVO*" | YES | TOTAL | p-valor |
|---|---|---|---|---|
| NO | 13 (92,9%) | 43 (93,5%) | 56 (93,3%) | 1,000** |
| YES | 1 (7,1%) | 3 (6,5%) | 4 (6,7%) | |
| Total | 14 (100%) | 46 (100%) | 60 (100%) | |

**Fisher's Exact Test

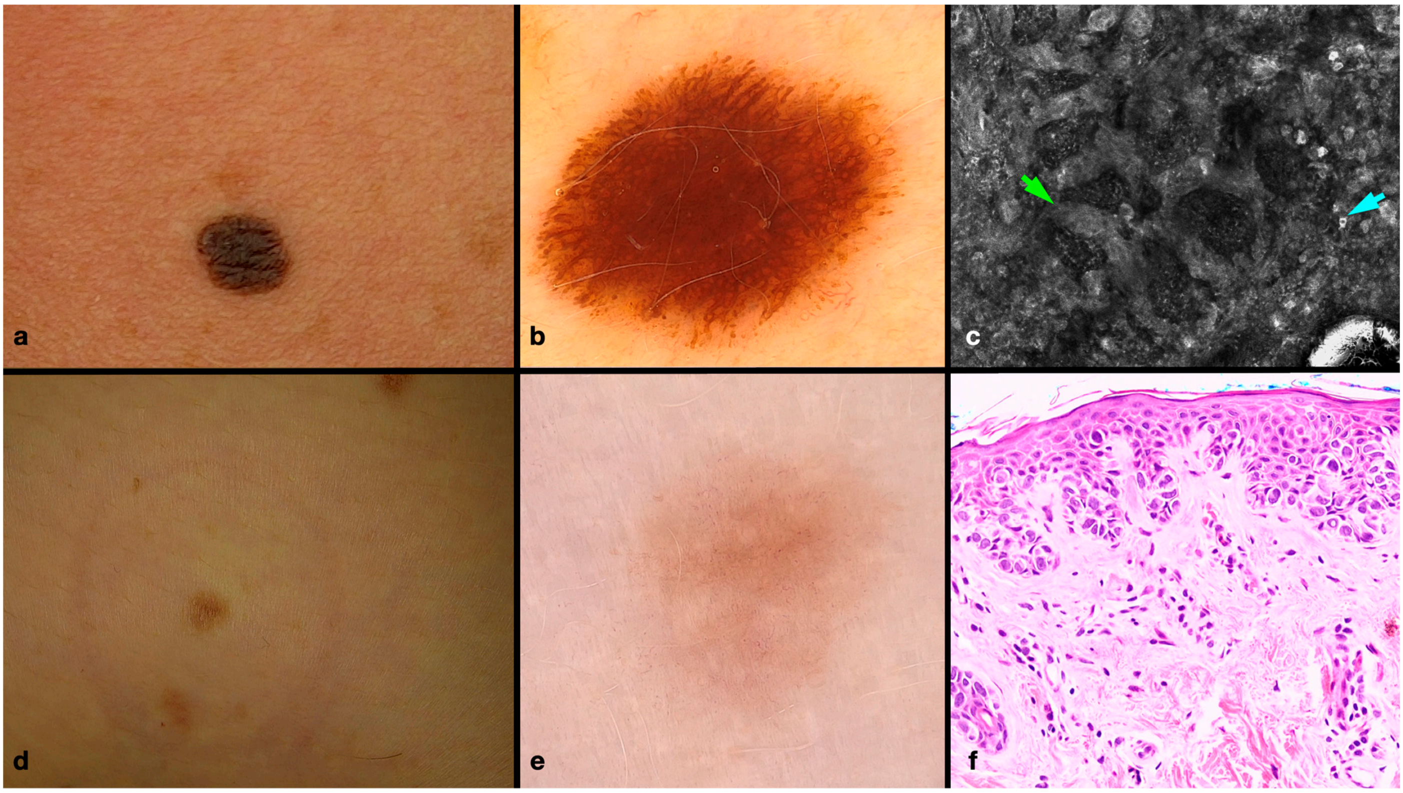


**Figure S1. Female patient: two *in-situ* melanoma, superficial spreading type, not associated with nevus ("*de novo*"). (a)** Clinical aspect of a well-defined, hyperpigmented nevus-like lesion on the right upper abdomen. (**b)** Contact polarized dermoscopy (CPD) with a magnification of 20x allows the observation of a starburst global pattern, with streaks, teared globules, central blotch (irregular hyperpigmented structureless areas), and peripheral brown circles (dark rim with tan central area). It was excised immediately after the first visit in June 2018. (**c)** RCM at the level of the DEJ reveals thickened interpapillary spaces (green arrow) and atypical round nucleated bright cells (teal arrow). **(d)** Clinical aspect of a left hypogastric lesion, well-defined, hypopigmented, nevus-like, that was followed for 3 years before suspicion and excision. **(e)** CPD (20x) shows a homogeneous global dermoscopic pattern, with structureless brown, targetoid structures (single dotted vessels in papillary spaces) and scattered polymorphous vessels (dotted and linear). The changes noticed over time were the gain of a very thin pigment network and an increase in the number of vessels. **(f)** Details of cytological atypia (hyperchromasia, evident nucleoli, karyomegaly, ample, and eosinophilic cytoplasm) (H&E 200x). *Clinical, dermoscopy and RCM hand-held probe individual image courtesy of Dra. Ana Maria Sortino. Histopathology images courtesy of Dr. Clovis Antônio Lopes Pinto and Dra. Rafaela Brito de Paula.*

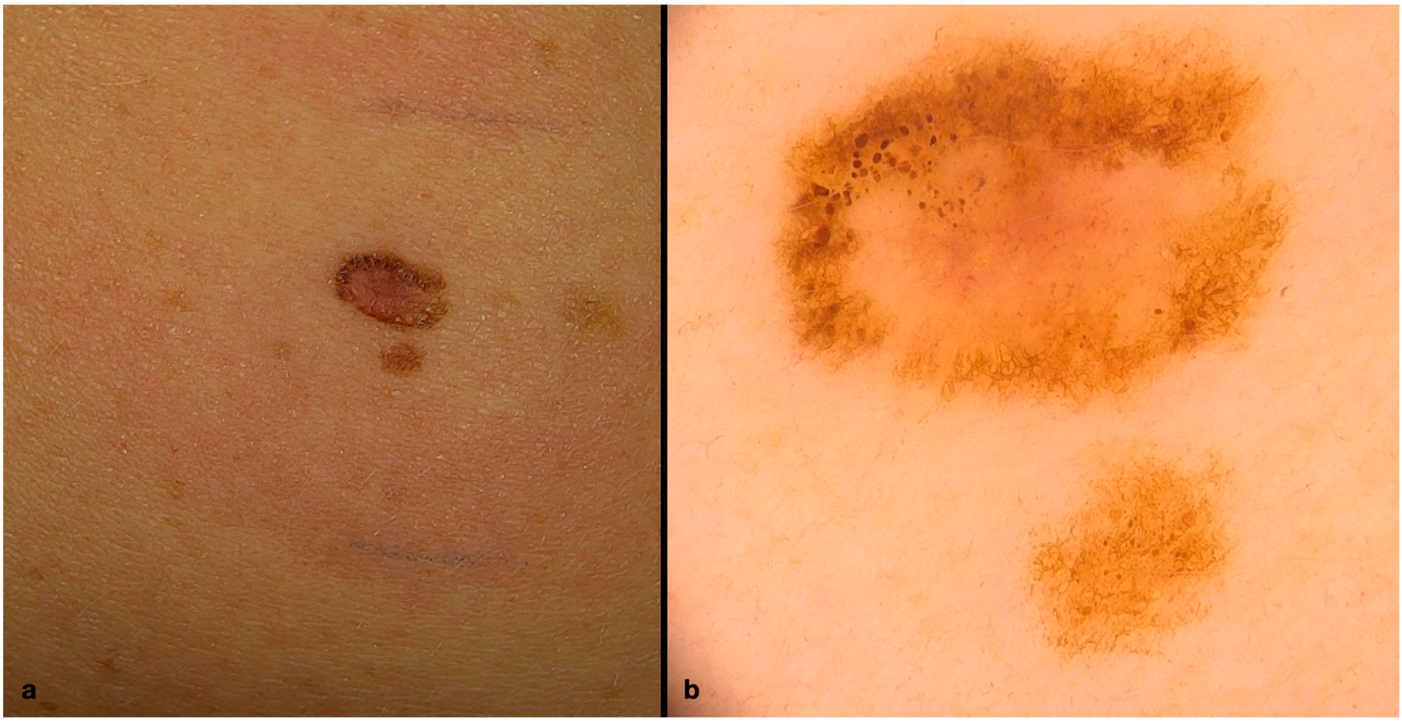


**Figure S2. Female patient: *in-situ* melanoma, superficial spreading type, associated with juncional nevus. (a)** Clinical image of a well-defined, synchronic typical (ABCD) melanoma, on the dorsal trunk. It was excised immediately after the first visit in June 2018. (**b)** Contact polarized dermoscopy (CPD) with a magnification of 20x allows the observation of focal irregular globules, peripheral atypical pigment network, central scar-like area with gray dots. *Clinical, and dermoscopy images courtesy of Dra. Ana Maria Sortino.*

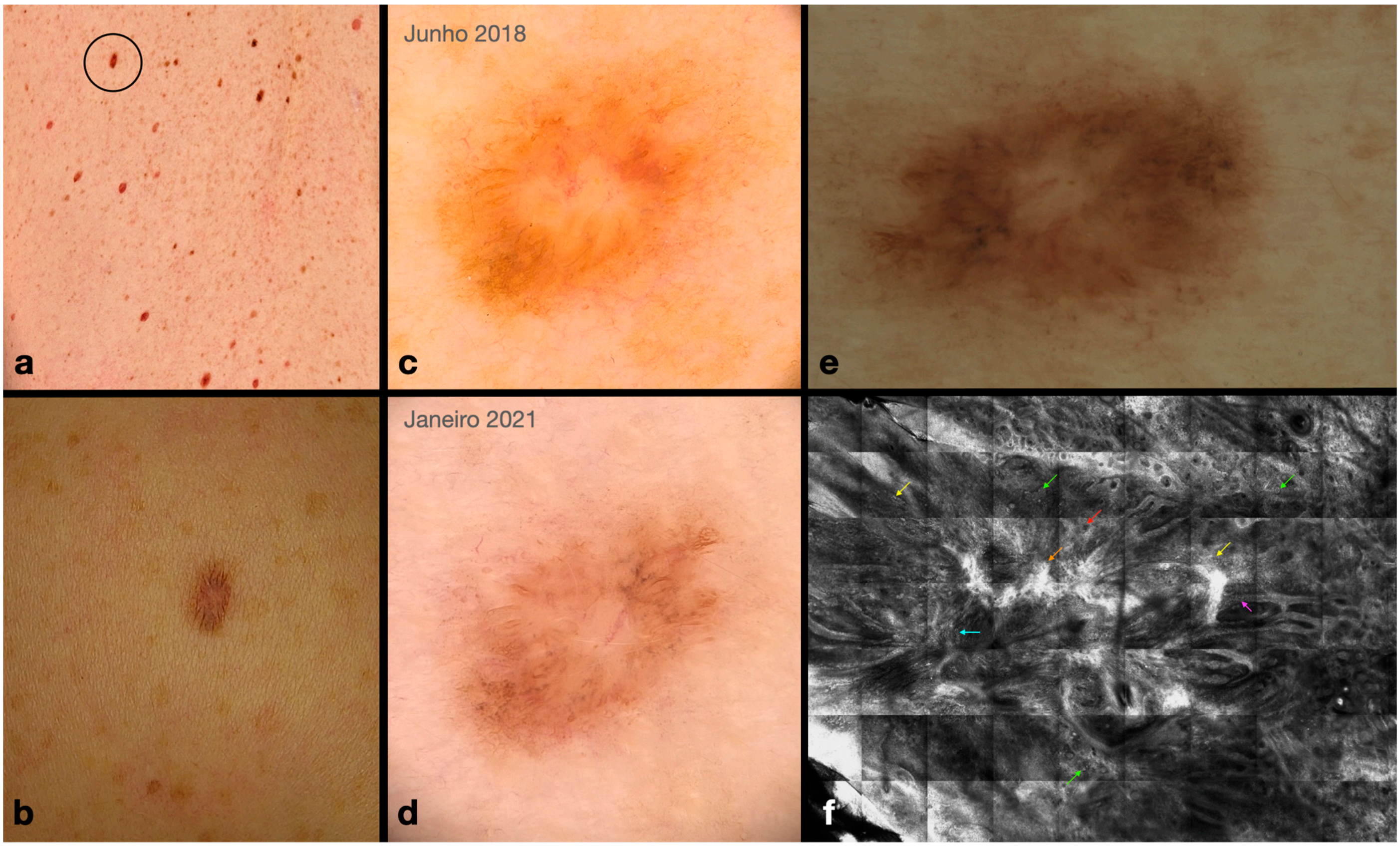


**Figure S3. Female patient: *in-situ* melanoma, lentigo maligna type, associated with dermal nevus. There is both histological inflammatory infiltrate and regression. (a)** Overview image of many nevi from the patient's back. The circle evidences the SPM. **(b)** Clinical image of a nevus-like pigmented lesion on the left dorsal trunk. **(c)** Baseline CPD (20x) allows the recognition of central structureless area with focal polymorphous vessels, an atypical pigmented network, and streaks at the periphery. **(d)** SDDI with CPD (20x) shows an increased central structureless area, peripheral atypical pigmented network, brown dots and granules, and brown circles. This significant dermoscopic regression was noticed after 31months of SDDI. **(e)** Non-polarized dermoscopy image built into the confocal machine. **(f)** RCM at the DEJ discloses a poorly demarcated lesion, with atypical meshwork (pink arrow), atypical cells as round nucleated, dendritic and hyporeflective (yellow arrows), multiple perpendicular vessels (red arrow), peripheral dense-sparse nests (green arrows), bright collagen (orange arrow), and small bright cells (inflammatory infiltrate). *Clinical, dermoscopy, and RCM wide-probe mosaic close-up images courtesy of Dra. Ana Maria Sortino.*

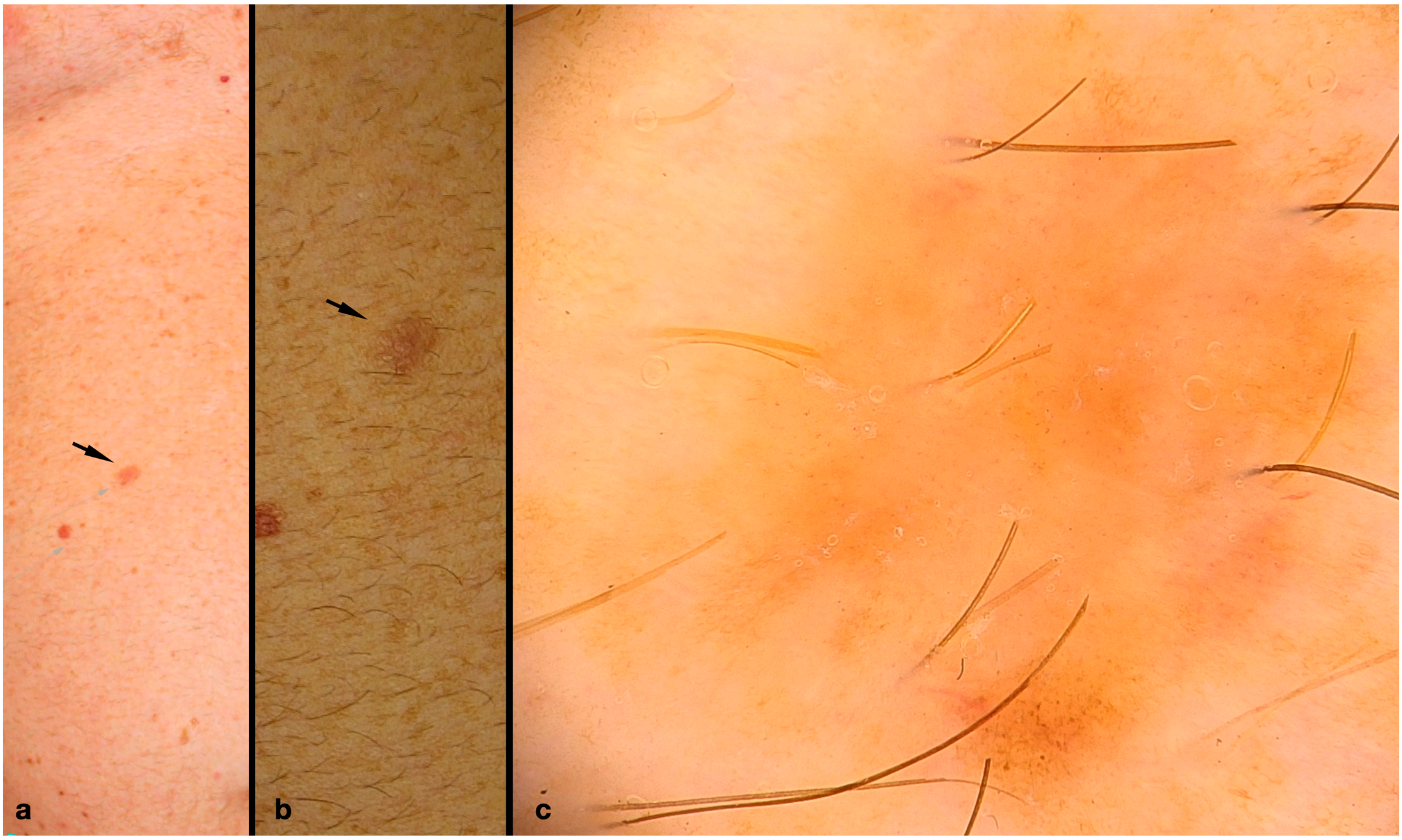


**Figure S4. Male patient: *in-situ* melanoma, lentiginous type, associated with a compound nevus that was immediately removed after the first TBM in July 2018. (a)** Amelanotic melanocytic lesion on the abdomen. **(b)** Close-up clinical image showing a skin-colored oval plaque. **(c)** Close-up of CPD (20x) allows the observation of a tan structureless background with scattered polymorphous vessels and peripheral focal gray dots and granules. *Clinical and dermoscopy images courtesy of Dra. Ana Maria Sortino.*

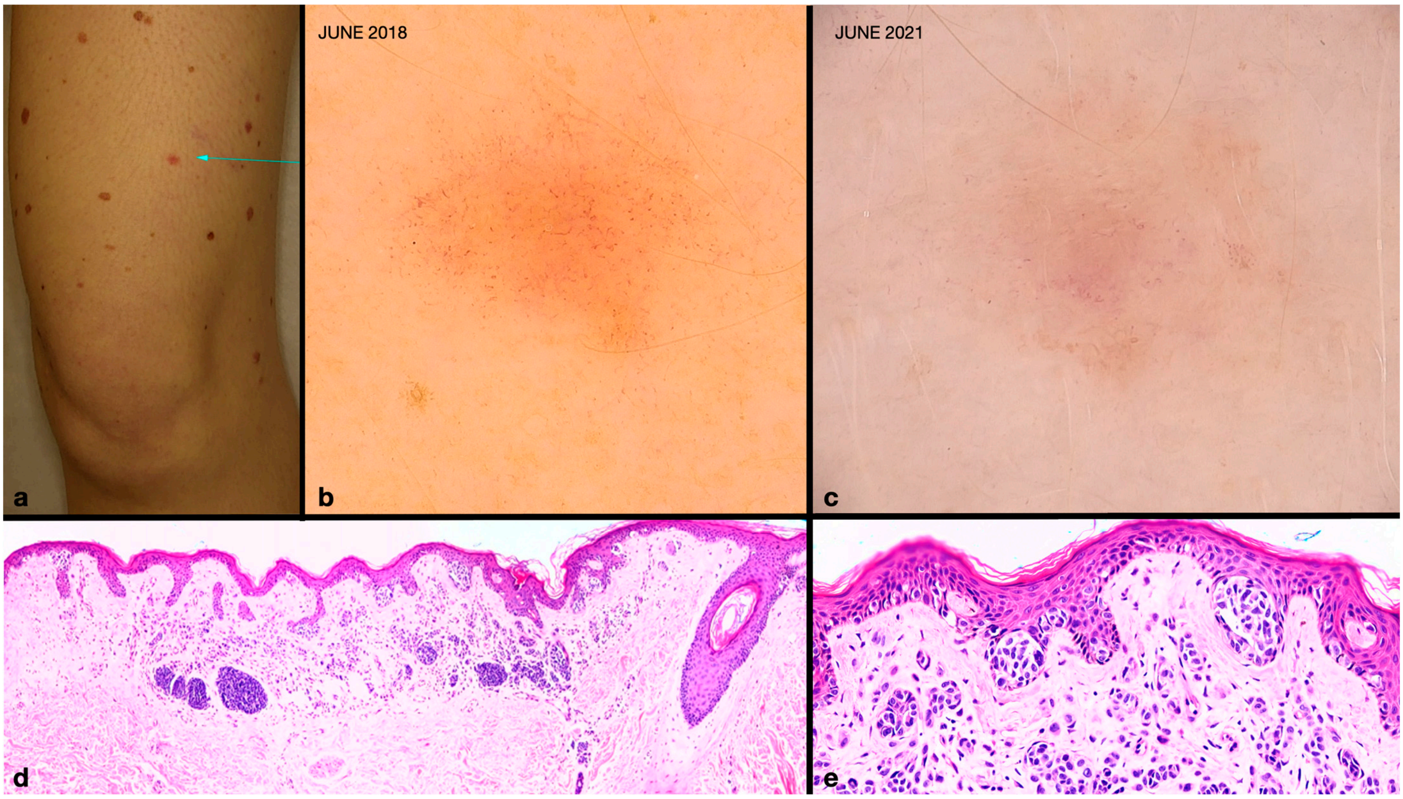


**Figure S5. Female patient: *in-situ* melanoma, superficial spreading type, associated with compound nevus. (a)** Clinical aspect of an amelanotic lesion on the leg. There are many lesions with this clinical morphology. (**b)** Baseline CPD (20x) allows the observation of a structureless background with scattered polymorphous vessels (dotted and linear). (**c)** The nevus was followed by SDDI for 3 years until significant change occurred by gain of peripheral brown dots and shiny white lines. **(d)** Panoramic view demonstrating compound melanocytic nevus (H&E 40x). **(e)** At higher magnification, in addition to the compound nevus, an isolated proliferation of atypical melanocytes can be seen in the epidermis, along with a pagetoid rise of atypical melanocytes (H&E 200x). *Clinical and dermoscopy images courtesy of Dra. Ana Maria Sortino. Histopathology images courtesy of Dr. Clovis Antônio Lopes Pinto and Dra. Rafaela Brito de Paula.*

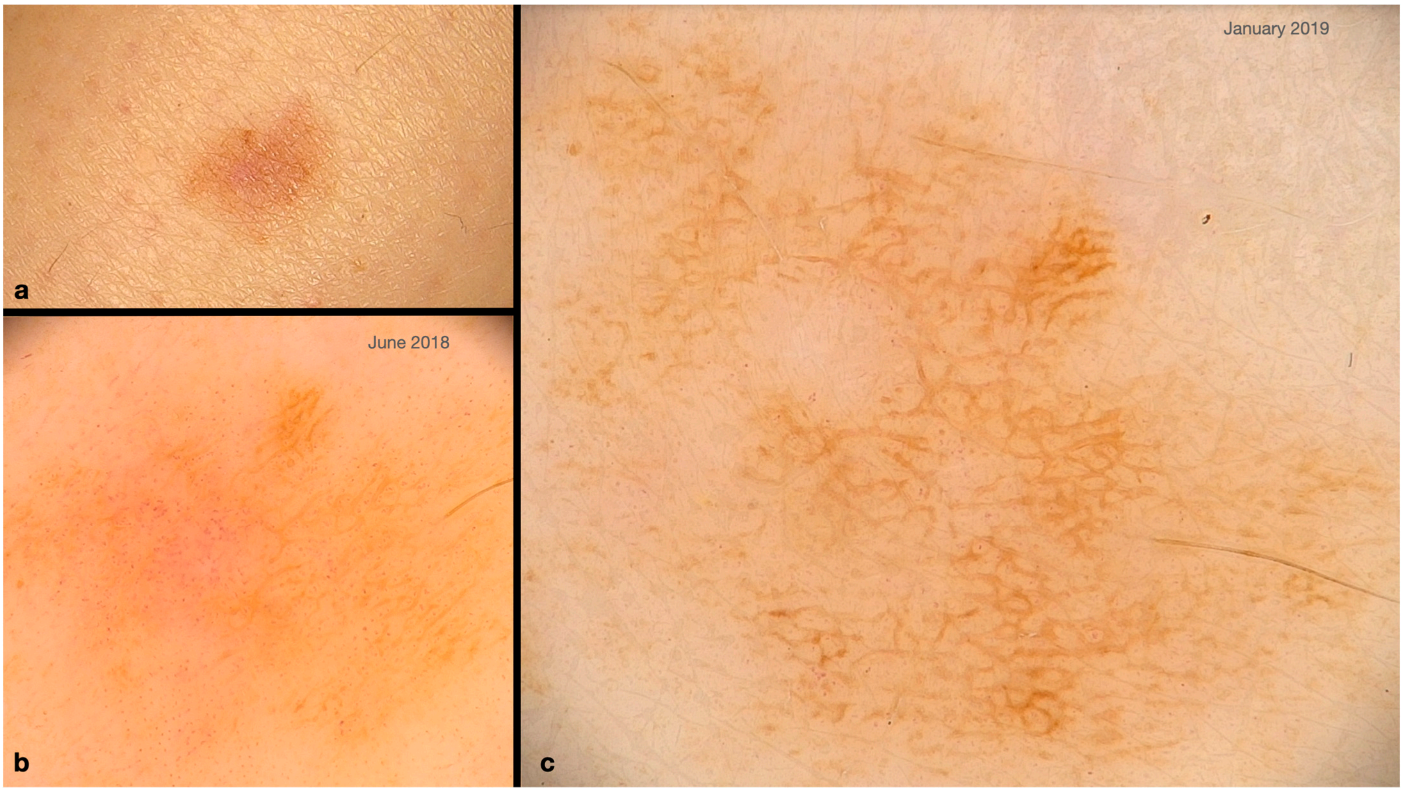


**Figure S6. Female patient: *in-situ* melanoma, superficial spreading type, associated with dermal nevus. (a)** Clinical image of a hypopigmented, ill-defined, melanocytic lesion on the left lateral lower leg. **(b)** Baseline CPD (20x) allows the identification of a very light atypical network and scattered dotted vessels. **(c)** Follow-up image close-up of CPD (20x), after seven months, shows increase, thickening and darkening of the irregular pigmented network. A structureless hypopigmented area replaced the dotted vessels. New targetoid structures (single dotted vessel or globule within a papillary space) appeared, along with globules in the network, and more visible streaks. *Clinical and dermoscopy images courtesy of Dra. Ana Maria Sortino.*

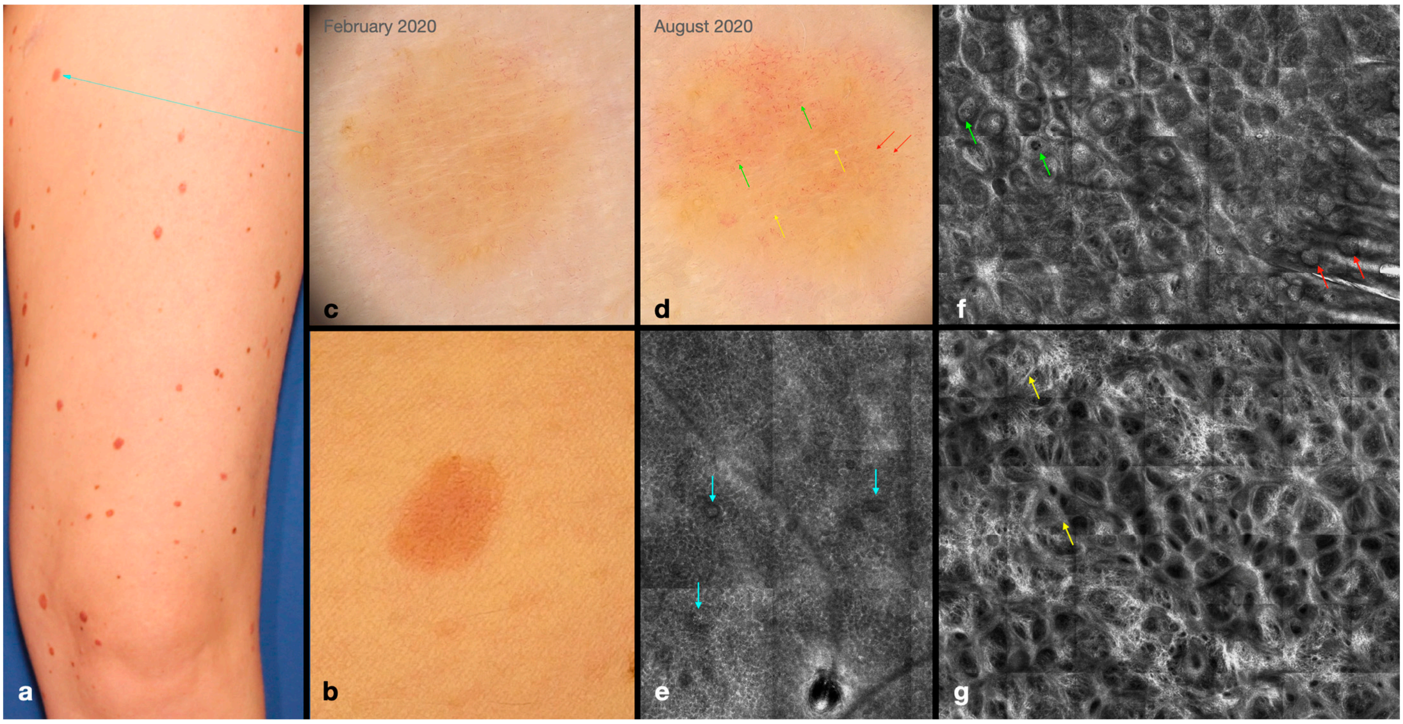


**Figure S7. Female patient: *in-situ* melanoma, superficial spreading type, associated with dermal nevus. (a)** On follow-up with TBSP a new brownish-pink melanocytic lesion was identified on the left inner thigh (teal arrow). (**b)** Close-up clinical image of a nevus-like melanoma. **(c)** Baseline CPD (20x) allows the observation of a tan structureless background with shiny white lines and scattered polymorphous vessels (dotted and linear). (**d**) SDDI (CPD-20x) after 6 months shows significant changes with increase in the number of polymorphous vessels (green arrows), shiny white lines (yellow arrows), and peripheral targetoid small globules is a linear fashion (red arrows). **(e)** RCM at the epidermal level evidence an atypical honeycomb pattern, with dark oval spaces, some with a central nest of small bright cells, that are surrounded by a dark rim (teal arrows). **(f)** At the DEJ multiple perpendicular vessels (green arrows) and irregular peripheral dense-sparse nests surrounded by a dark rim (red arrows). **(g)** At the superficial dermal level, an atypical meshwork is seen, with widening of interpapillary spaces and presence of thin dendritic bright cells (yellow arrows), along with areas of thickened collagen. *Clinical, dermoscopy, and RCM wide-probe mosaic close-up images courtesy of Dra. Ana Maria Sortino.*

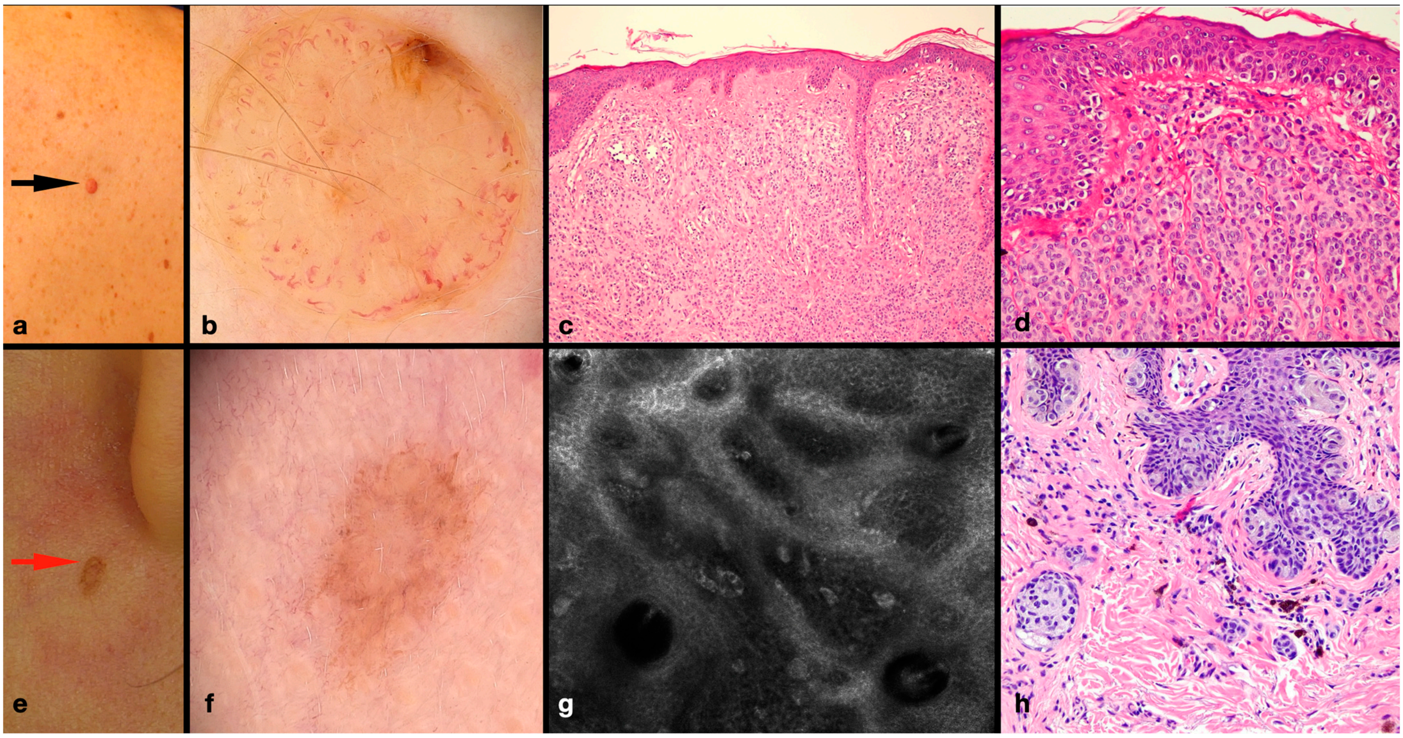


**Figure S8. Female patient. (a)** Dome shaped melanocytic lesion. Clinical impression was of a common dermal nevus, however the patient asked for the excision. **(b)** CPD (20x) image shows comma/curved vessels and a brown halo. **(c)** Photomicrograph demonstrating typical dermal nevus. In the upper right corner, there is a junctional melanocytic proliferation with cytological atypia and the beginning of pagetoid ascension (H&E 40x). **(d)** Contiguously proliferated junctional component, and with foci of pagetoid ascension, contrasting with the dermal component (H&E 200x). **The diagnosis was *in-situ* melanoma, superficial spreading type, associated with dermal nevus. (e)** New infra-auricular hypopigmented melanocytic lesion found by the dermatologist on TBSP. **(f)** CPD (20x) image of a structureless brown lesion with peripheral irregular streaks. **(g)** Under RCM there is thickening of the juncional spaces. Dermal nucleated round cells, multiple perpendicular vessels in the papillae, and dermal melanophages are seen. (**h)** In this histological field, one can note the small invasive focus in the papillary dermis, whose cells present the same atypical cytological characteristics of the junctional component (H&E 200x). **The final diagnosis was invasive melanoma, superficial spreading type, not associated with nevus ("*de novo*"), Breslow 0.2 mm, mitotic index of 0/10 CGA.** *Clinical, dermoscopy and RCM hand-held probe individual image courtesy of Dra. Ana Maria Sortino. Histopathology images courtesy of Dr. Clovis Antônio Lopes Pinto and Dra. Rafaela Brito de Paula.*

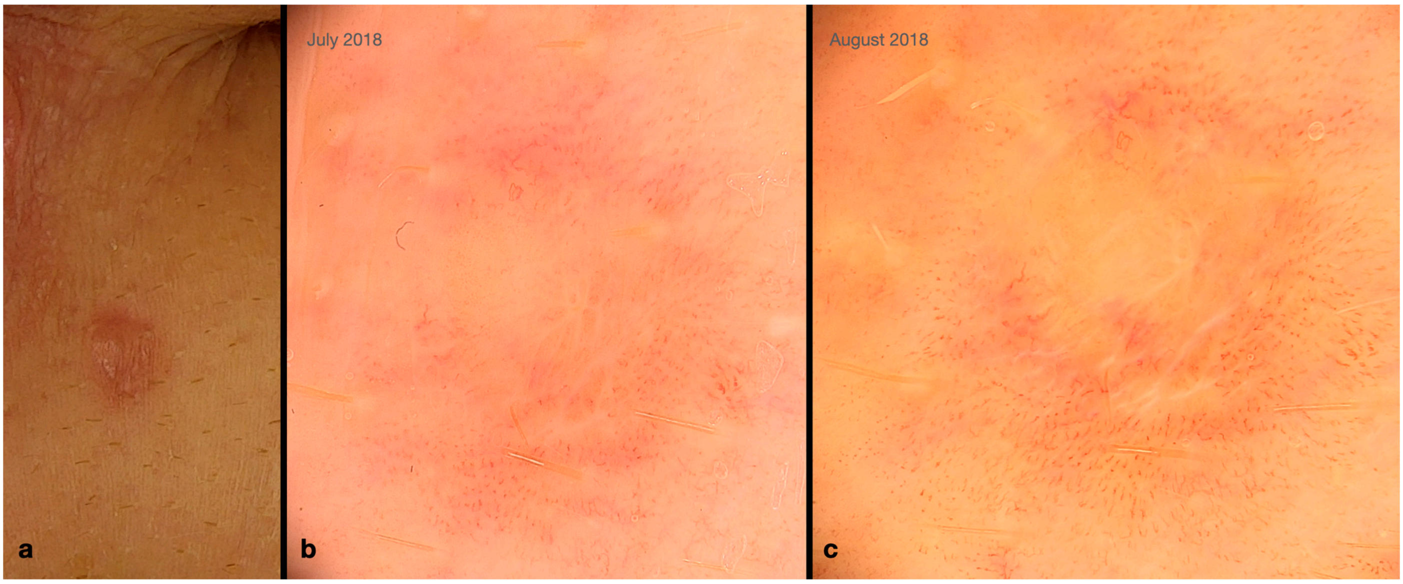


**Figure S9. Female patient: *in-situ* melanoma, superficial spreading type, associated with compound nevus. (a)** Clinical image of a perineal ill-defined, synchronic amelanotic melanoma. It was excised immediately after the third visit in August 2018. (**b)** Baseline CPD (20x) shows an asymmetric multiple component global pattern, with structureless areas, shiny white lines, and peripheral polymorphous vessels. (**c)** One month later, an increase of shiny white lines and polymorphous vessels is noticed on CPD (20x). Excisional biopsy with 2mm margins was performed. *Clinical and dermoscopy images courtesy of Dra. Ana Maria Sortino.*

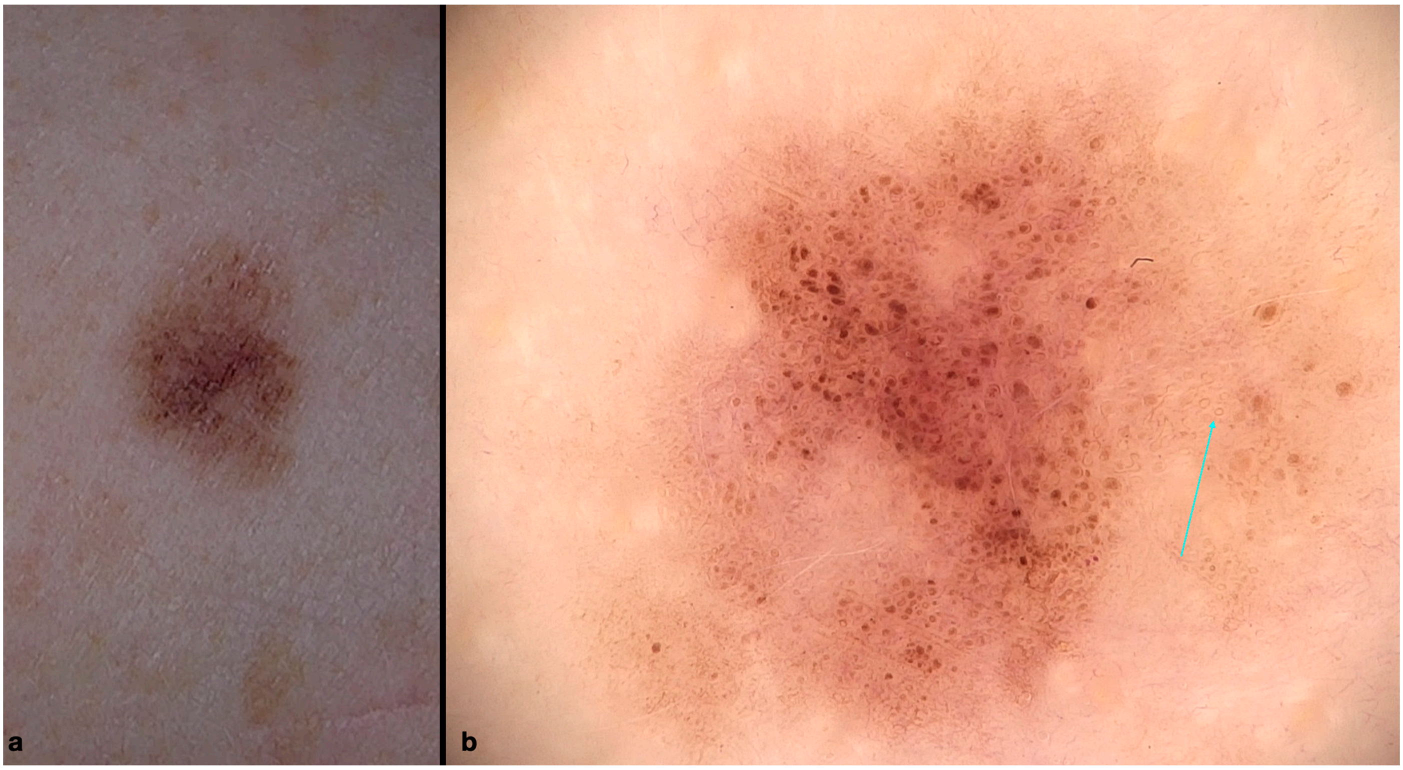


**Figure S10. Female patient: *in-situ* melanoma, superficial spreading type, associated with dysplastic compound nevus. (a)** Clinical image of an ill-defined melanocytic lesion on the ventral trunk. (**b)** CPD (20x) shows an asymmetric multiple component global pattern, with irregular and targetoid globules, structureless tan areas and peripheral brown circles (teal arrow). *Clinical and dermoscopy images courtesy of Dra. Ana Maria Sortino.*

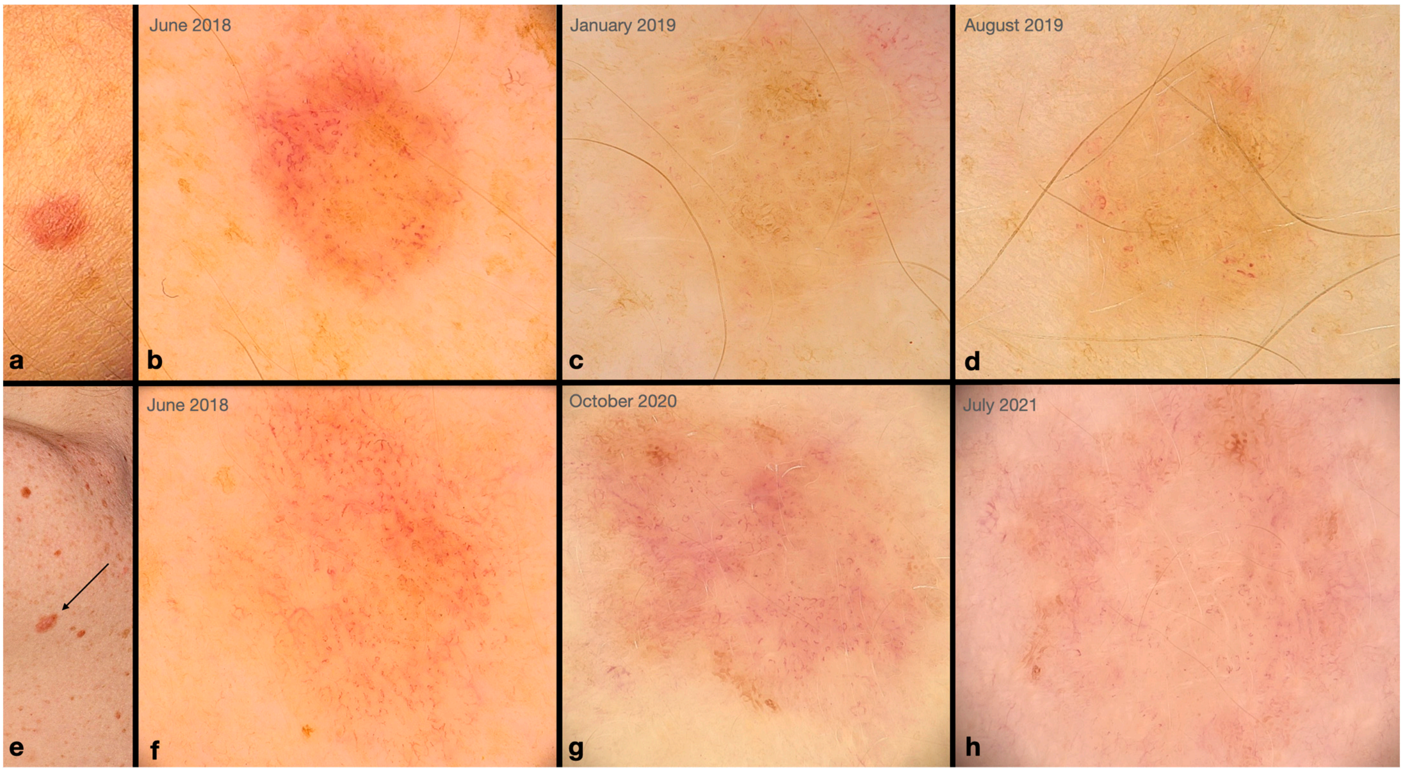


**Figure S11. Examples of two melanomas from the female patient with histological inflammatory infiltrate and no regression. (a)** Clinical image of a nevus-like amelanotic lesion on the left lower arm. Change was noticed after 12.2 months of SDDI. **(b)** Baseline CPD (20x) allows the recognition of central brown circles and dotted vessels. At the periphery focal polymorphous vessels are seen. **(c)** SDDI with CPD (20x) shows an increased number of central brown circles, targetoid structures (dotted vessel), brown dots and granules, and shiny white lines. **(d)** After 7 months, with digital dermoscopy (CPD-20x), a less impressive change is noticed, yet excision was recommended due to previous modifications. **The diagnosis was *in-situ* melanoma, superficial spreading type, associated with compound nevus. (e)** Clinical image of a nevus-like hypopigmented lesion on the left posterior shoulder (arrow). **(f)** Baseline CPD (20x) shows a tan background with brown dots and granules, hypopigmented structureless areas and polymorphous vessels. **(g)** SDDI with CPD (20x) evidencing the gain of focal atypical pigment network, streaks, and peripheral brown circles. In the center there in an increase in structureless areas and polymorphous vessels. **(h)** The final SDDI with CPD (20x) allows to see the continuous enlargement of the melanocytic lesion, with acquired central white shiny lines. It was followed for 31 months by SDDI prior to excision. **The diagnosis was an invasive melanoma, superficial spreading type, associated with a dermal nevus, Breslow 0.4 mm, and mitotic index of 1/10 CGA.** *Clinical and dermoscopy images courtesy of Dra. Ana Maria Sortino.*

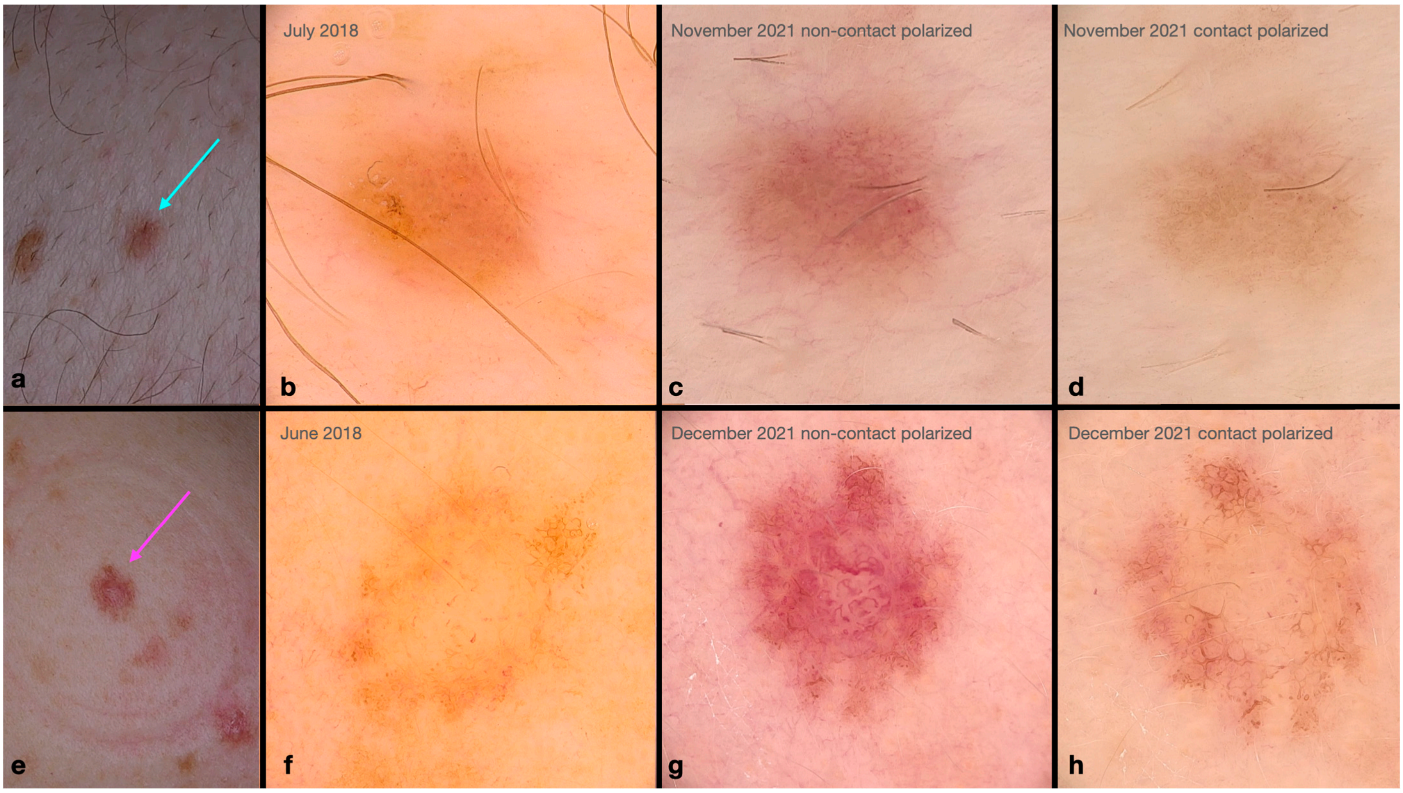


**Figure S12. Examples of two *in-situ* melanomas without histological inflammatory infiltrate and no regression. (a) Male patient:** lateral trunk with multiple tan symmetric nevi. Change was noticed after 38.8 months of SDDI. **(b)** Baseline CPD (20x) allows the observation of a structureless tan lesion with grey dots and granules. (c) Follow-up NCPD (20x) shows irregular pinkish-brown globules and dotted vessels. **(d)** CPD (20x) presents a negative network, brown circles, and dotted vessels. **The diagnosis was an *in-situ* melanoma, superficial spreading type, not associated with nevus ("*de novo*"). (e) Female patient:** lateral left upper arm with multiple Clark nevi. Change was noticed after 40.2 months of SDDI. **(f)** Baseline CPD (20x) allows the observation of a peripheral atypical pigment network, hypopigmented center with linear vessels. **(g)** Follow-up NCPD (20x) shows an increase in number of polymorphous vessels throughout the lesion. **(h)** CPD (20x) presents a thickened peripheral atypical pigment network, forming polygons, brown circles, targetoid structures (central globule or dotted vessel), hypopigmented center with shiny white lines. **The diagnosis was an *in-situ* melanoma, superficial spreading type, associated with a dysplastic nevus.** *Clinical and dermoscopy images courtesy of Dra. Ana Maria Sortino.*

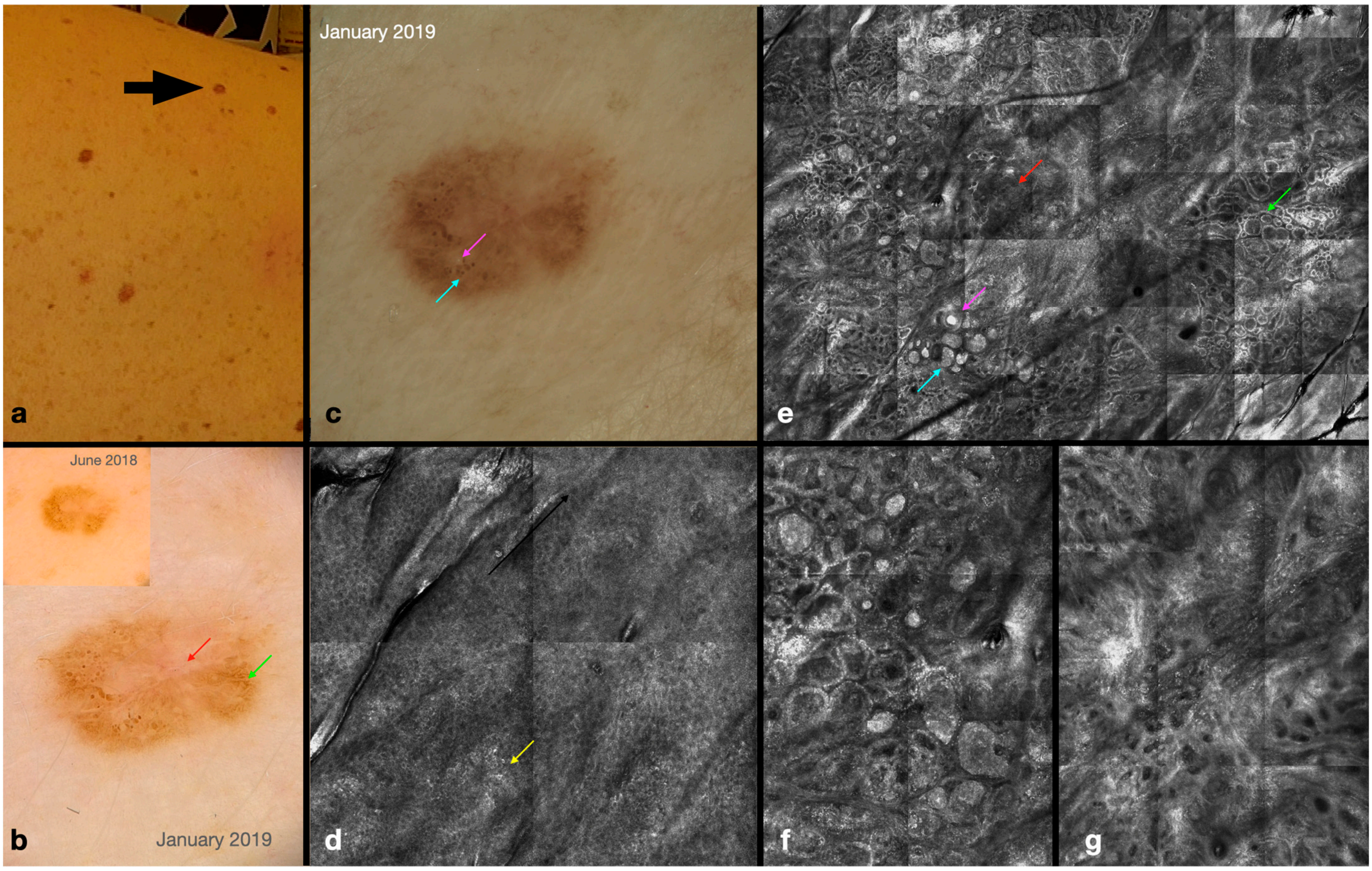


**Figure S13. Female patient: invasive melanoma, superficial spreading type, not associated with nevus ("*de novo*"), Breslow 0.2 mm, mitotic index of 0/10 CGA. (a)** Clinical image of a nevus-like melanocytic lesion on the trunk. **(b)** CPD (20x) shows, on the upper corner, the baseline dermoscopy with an asymmetric multiple component global pattern (June 2018). Follow-up with SDDI, after 6,9 months (January 2019), disclosures gain of focal irregular and targetoid globules, peripheral atypical pigment network (green arrow), and a central scar-like area (red arrow). **(c)** Non-polarized dermoscopy image built into the RCM for lesion location referral. There is a milia-like cyst (magenta arrow) and irregular focal globules (teal arrow). **(d)** RCM at the epidermal level shows an atypical honeycomb pattern with scattered small bright polymorphous cells (yellow arrow). **(e)** At the DEJ, the milia cyst is evident (magenta arrow), surrounded by irregular dense-sparse nests (teal arrow), an area of non-edged papillae in the center (red arrow) and edged papillae in the periphery (green arrow). The abrupt transition can be visualized. (f) Close-up RCM image of irregular peripheral dense-sparse nests. (g) At the superficial dermal level, an atypical meshwork is seen, with widening of interpapillary spaces and presence of thin dendritic bright cells, along with areas of thickened collagen, and dermal small bright cells (inflammatory infiltrate). *Clinical, dermoscopy, and RCM wide-probe mosaic close-up images courtesy of Dra. Ana Maria Sortino.*

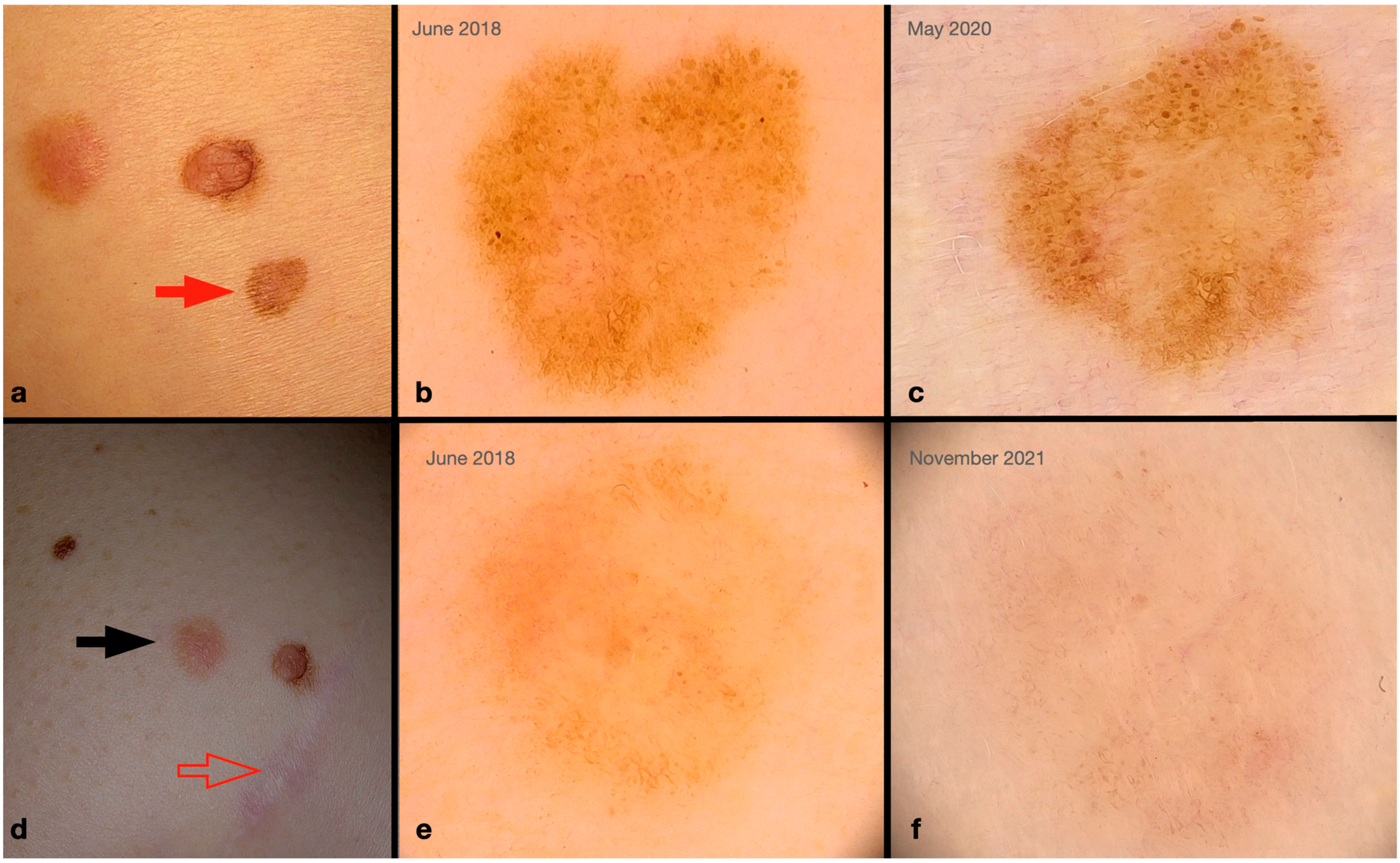


**Figure S14. Female patient. (a)** Three different melanocytic nevi on the left lower lateral trunk. The macular brown nevi-like lesion (red arrow) was the first to have a noticeable change. **(b)** Dermoscopy (CPD-20x) image shows an asymmetric multicomponent pattern, with peripheral atypical pigment network and irregular globules. In the center a negative network and polymorphous vessels are seen. **(c)** SDDI (CPD-20x) after 22,9 months showed loss of central structures, replaced by a structureless area. **The diagnosis was *in-situ* melanoma, superficial spreading type, associated with compound nevus. (d)** A clinically unchanged, oval amelanotic melanocytic lesion (black arrow) near the scar of the excised CM (outlined red arrow). **(e)** CPD (20x) baseline image shows a symmetric multicomponent lesion, with peripheral atypical network and central globules with few dotted vessels. **(f)** SDDI (CPD-20x) after 40,2 months showed loss of central structures, replaced by a structureless area with shiny white lines, scattered dotted vessels, peripheral brown circles, and targetoid globules. **The diagnosis was invasive melanoma, superficial spreading type, associated with dysplastic compound nevus, Breslow 0.5 mm, mitotic index of 0/10 CGA.** *Clinical, dermoscopy and RCM hand-held probe individual image courtesy of Dra. Ana Maria Sortino.*

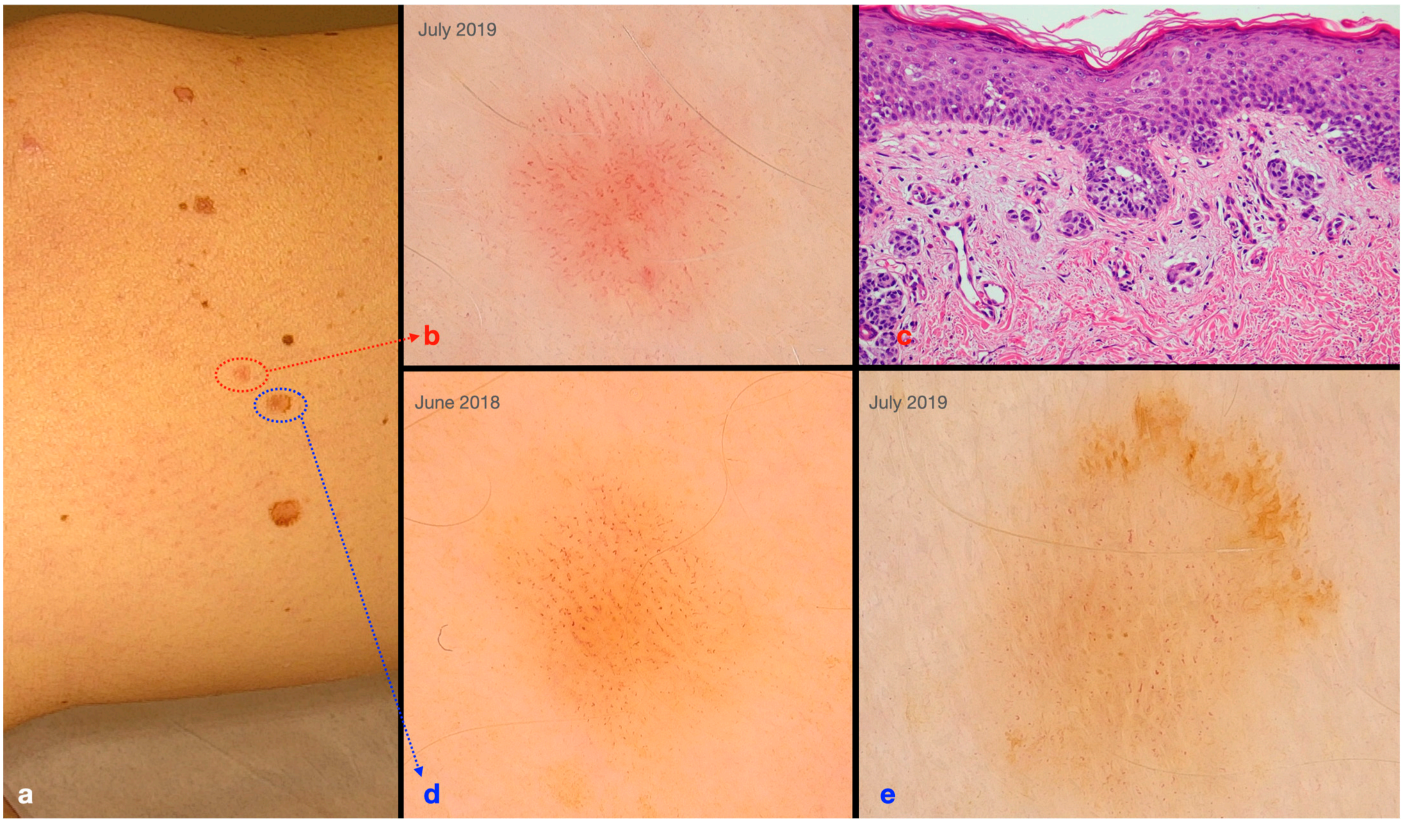


**Figure S15. Female patient. (a)** Clinical picture of the medial right medial knee showing a new pink lesion (red dotted circle) found on follow-up TBSP, and a growing nevus (blue dotted circle). **(b)** CPD (20x) of the pink lesion shows polymorphous vessels in a starburst disposition. The lesion was immediately excised. **(c)** Compound melanocytic proliferation is observed, and atypical melanocytes in superficial portions of the epidermis (pagetoid ascension) are seen. Both junctional and dermal components show nuclear atypia. The invasive area is focal and restricted to the papillary dermis (H&E 200x). **The diagnosis was an invasive melanoma, superficial spreading type, Breslow 0.3 mm, mitotic index of 0/10 CGA, associated with a dermal nevus. (d)** Baseline CPD (20x) allows the observation of a tan structureless background with dotted vessels in a linear and parallel distribution **(e)** SDDI with CPD (20x) shows gain of a negative network, targetoid dotted vessels, and irregular peripheral streaks. The lesion was excised after one year. **Histology showed an *in-situ* melanoma, superficial spreading type, associated with a dermal nevus.** *Clinical and dermoscopy images courtesy of Dra. Ana Maria Sortino. Histopathology images courtesy of Dr. Clovis Antônio Lopes Pinto and Dra. Rafaela Brito de Paula.*

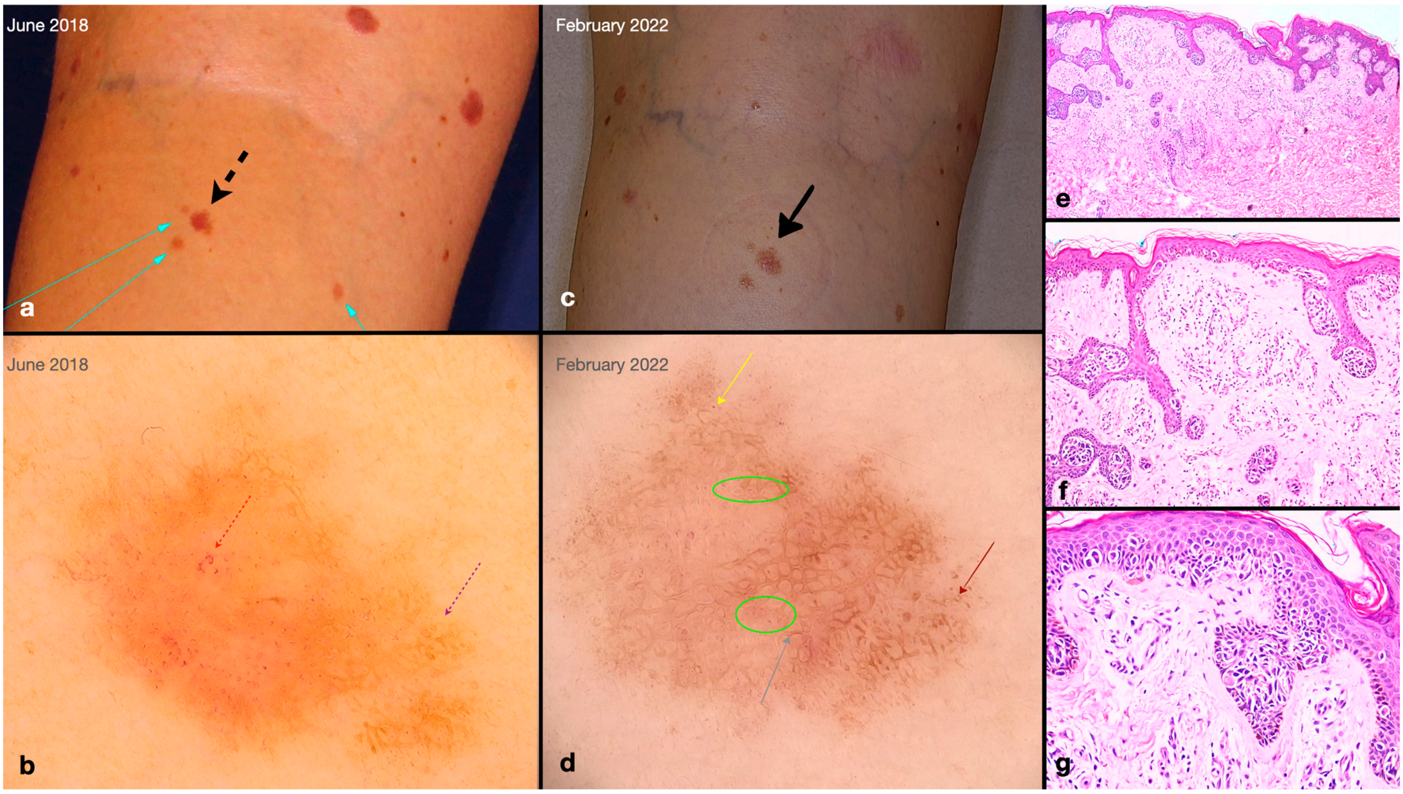


**Figure S16. Female patient: invasive melanoma, superficial spreading type, not associated with nevus ("*de novo*"), Breslow 0.94 mm, mitotic index of 0/10 CGA. (a).** Clinical aspect of a popliteal pinkish-tan melanocytic lesion. (**b).** Baseline CPD (20x) allows the observation of a tan structureless background, with polymorphous vessels (red dotted arrow), peripheral atypical pigment network (purple dotted arrow), irregular globules, and streaks. (**c).** Clinical enlargement and darkening of the lesion are noticed over time. **(d).** The lesion was followed by SDDI for 43.9 months with subtle yet constant modifications, until changes were significant enough for removal approval by the patient. CPD (20x) shows gain of a thicker and larger network (gray arrow), targetoid structures (yellow arrow), peripheral brown circles (brown arrow), and multiple (2 to 5) dotted vessels within the dermal papilla (green circles). **(e).** Panoramic view demonstrating compound melanocytic proliferation with prominent atypia in both components (junctional and dermal), characterizing an invasive melanoma (H&E 40x). **(f).** Photomicrograph with evidence of pleomorphic melanocytes in the epidermis, pagetoid dissemination, and nest formation. In the dermis, small focus of invasion (H&E 100x). **(g).** Higher magnification showing melanocytes with intense nuclear atypia and pagetoid rise (H&E 200x). *Clinical and dermoscopy images courtesy of Dra. Ana Maria Sortino. Histopathology images courtesy of Dr. Clovis Antônio Lopes Pinto and Dra. Rafaela Brito de Paula.*

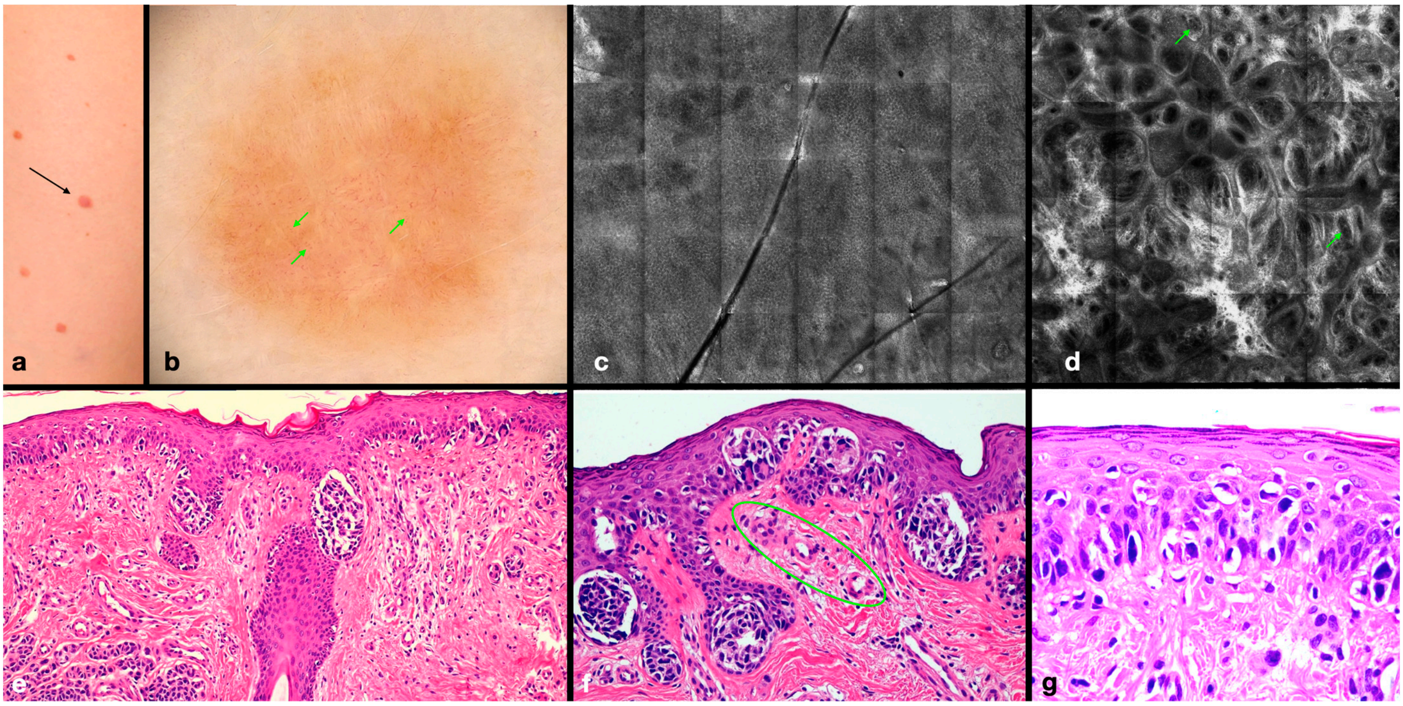


**Figure S17. Female patient: *in-situ* melanoma, superficial spreading type, associated with compound nevus. (a)** posterior right thigh. **(b)** Baseline CPD (20x) shows multiple dotted vessels within the dermal papillae, often aligned (green arrow). **(c)** RCM at epidermal level shows an atypical honeycomb pattern with scattered dark oval areas. **(d)** RCM at dermal level evidence of an atypical meshwork. Multiple perpendicular vessels are seen as multiple dark spaces with a central bright dot (green arrow) within the papillae. **(e)** Extensive architectural and cytological atypia of the junctional component are observed, spreading throughout the epidermis, and characterizing an *in-situ* melanoma. In the lower left corner, presence of associated nevus (H&E 100x). **(f)** At higher magnification, epidermal cytological atypia is better evaluated, with intense hyperchromasia of the nuclei and the presence of multinucleation. Focus of epidermal consumption by the *in-situ* melanoma, distributed in nests and isolated cells. At the dermis, vessels in a linear arrangement (green circle) (H&E 200x). (g) Higher magnification showing pagetoid spread of melanocytes (H&E 400x). *Clinical, dermoscopy, and RCM wide-probe mosaic close-up images courtesy of Dra. Ana Maria Sortino. Histopathology images courtesy of Dr. Clovis Antônio Lopes Pinto and Dra. Rafaela Brito de Paula.*

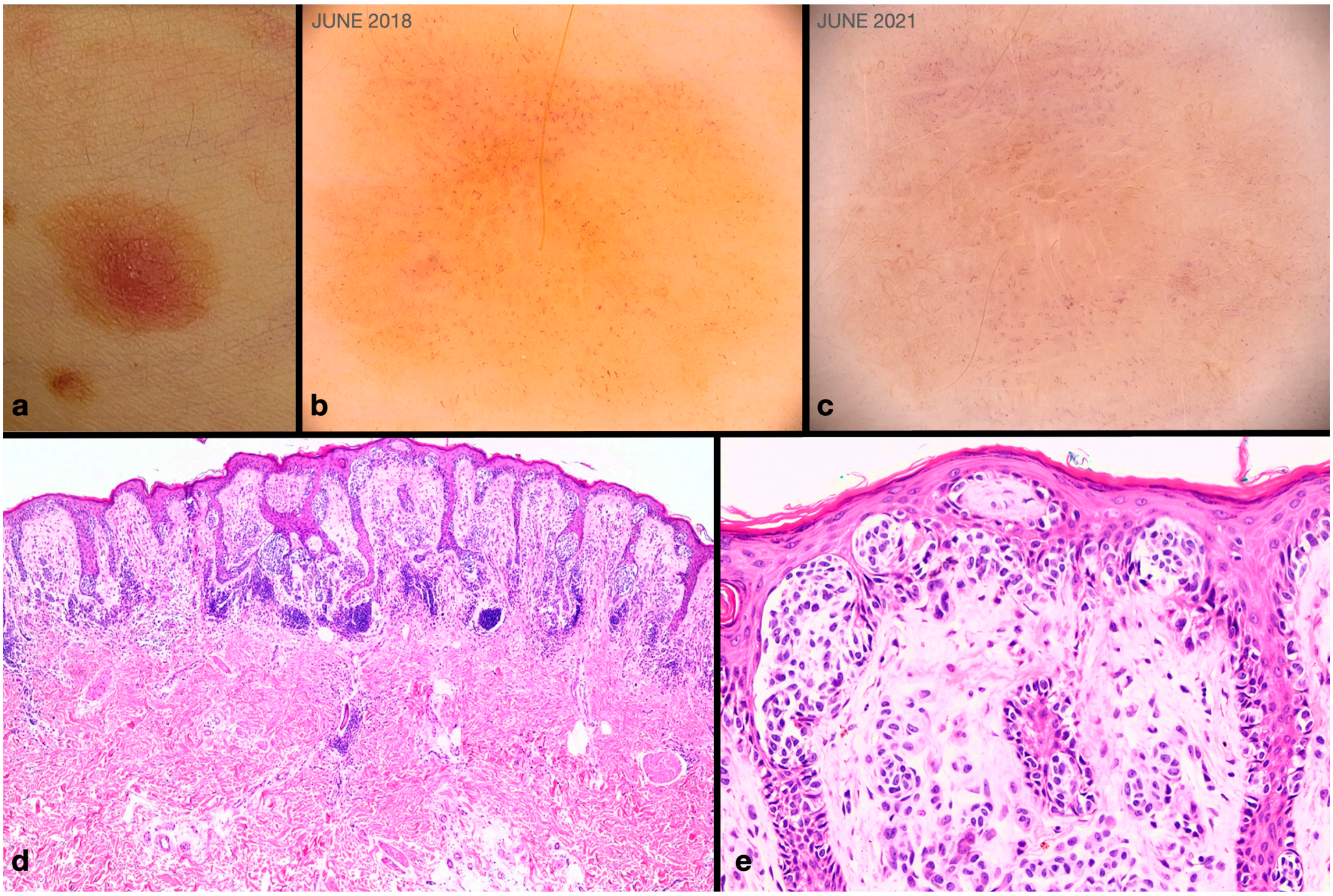


**Figure S18. Female patient: *in-situ* melanoma, superficial spreading type, associated with compound nevus. (a)** Clinically hypopigmented nevus-like melanocytic lesion on left knee. **(b)** Baseline CPD (20x) allows the identification of scattered polymorphous vessels and a central negative network over a structureless tan background. **(c)** Follow-up image (CPD-20x), after three years, shows multiple dotted vessels with papillary spaces, central shiny white lines, peripheral focal irregular pigmented network, and brown circles. **(d)** Panoramic view demonstrating compound melanocytic nevus (H&E 40x). **(e)** Epidermal consumption is observed and, in the corner on the right, one can see the spread of *in-situ* melanoma to the adnexal epithelium (H&E 200x). *Clinical and dermoscopy images courtesy of Dra. Ana Maria Sortino. Histopathology images courtesy of Dr. Clovis Antônio Lopes Pinto and Dra. Rafaela Brito de Paula.*

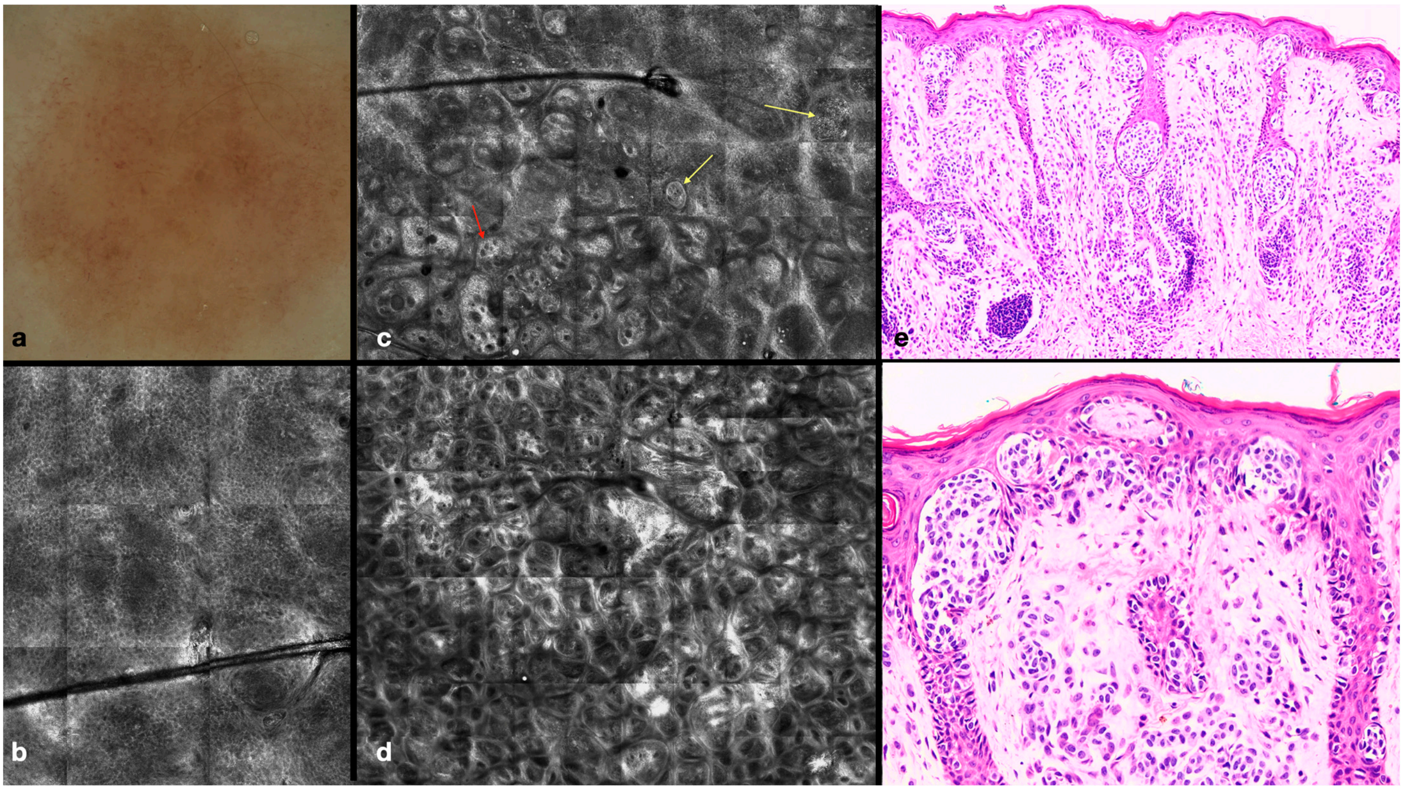


**Figure S19. Female patient: *in-situ* melanoma, superficial spreading type, associated with compound nevus (same lesion of Figure S18).** **(a)** lesion previously described on Figure A14.1. Non-polarized dermoscopy image built into the confocal machine. **(b)** At the epidermal level, RCM shows an atypical honeycomb pattern with scattered dark oval areas. **(c)** RCM close-up image of the DEJ shows irregular dense-sparse nests (yellow arrows) and multiple perpendicular vessels within the papillae (red arrow). **(d)** RCM at dermal level evidence an atypical meshwork and thick bright collagen. **(e)** Junctional and intradermal nests with maturation are observed, contrasting with the continuous junctional proliferation of isolated melanocytes (H&E 100x). **(f)** spread of *in-situ* melanoma to the adnexal epithelium (H&E 200x). *Clinical, dermoscopy, and RCM wide-probe mosaic close-up images courtesy of Dra. Ana Maria Sortino. Histopathology images courtesy of Dr. Clovis Antônio Lopes Pinto and Dra. Rafaela Brito de Paula.*

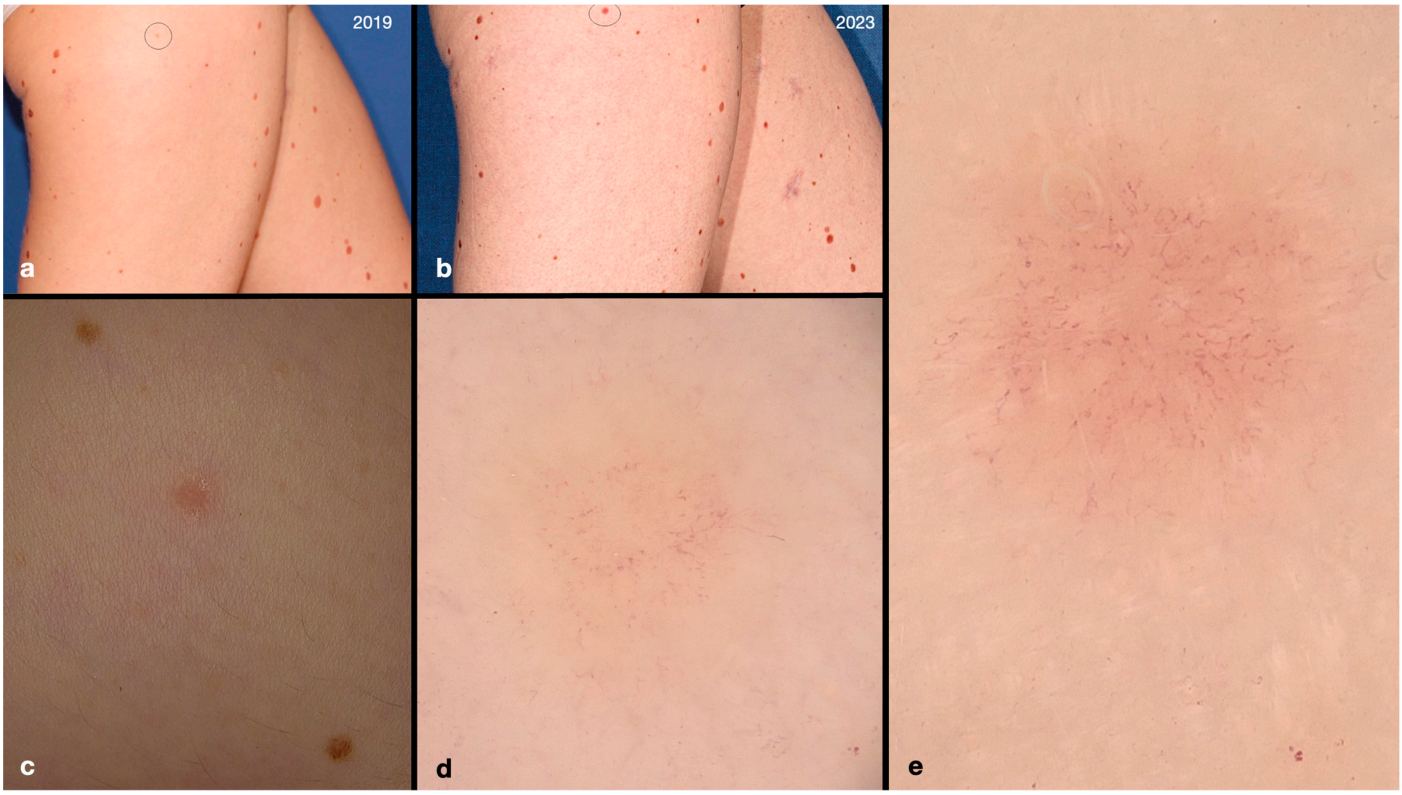


**Figure S20. Female patient: *in-situ* melanoma, superficial spreading type, associated with compound nevus. (a)** TBSP from 2019 showing a lesion that was not marked for follow-up at the time. It wasn't present at baseline TBSP from 2018. **(b)** TBSP from 2023 showing growth of an amelanotic lesion, that didn't show any change on previous TBSPs. **(c)** Clinical close-up image of a slightly palpable oval lesion, with a central pink color and a whitish rim. **(d)** Baseline contact non-polarized dermoscopy (CNPD-20x) shows thin polymorphous vessels over a skin-colored background. **(d)** A close-up image of CPD (20x) allows the visualization of white shiny lines and scattered polymorphous vessels. *Clinical and dermoscopy images courtesy of Dra. Ana Maria Sortino.*

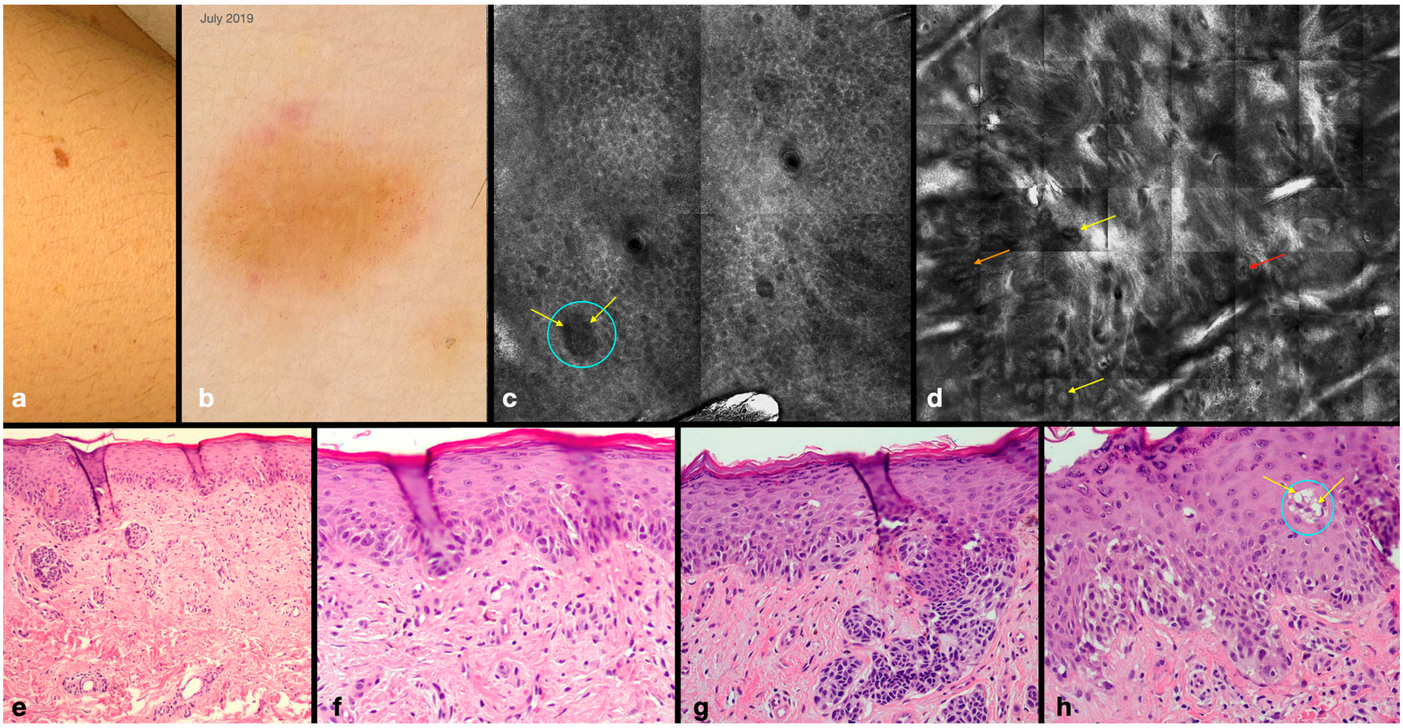


**Figure S21. Female patient: invasive melanoma, superficial spreading type, not associated with nevus ("*de novo*"), Breslow 0.4 mm, mitotic index of 0/10 CGA.** **(a)** Right lateral lower leg with a new pigmented oval melanocytic lesion found on TBSP. **(b)** Baseline CPD (20x) allows the observation of a brown structureless background, targetoid dotted vessels, central hypopigmented lines, peripheral light brown globules, and streaks. **(c)** RCM at epidermal level presents an atypical honeycomb pattern with a dark oval structure (teal circle) composed of hyporeflective pagetoid cells (yellow arrows). (d) RCM at DEJ evidence globular (yellow arrows) and linear (orange arrow) dense-sparse nests of small cells, and multiple perpendicular vessels within the papillae (red arrow). **(e)** Photomicrograph demonstrating composite melanocytic lesion with relevant cytological and architectural atypia in both the junctional and dermal components (H&E 100x). **(f)** At the highest magnification, a junctional component can be seen with contiguous proliferation foci through the basal layer of the epidermis and areas of pagetoid ascension (H&E 200x). **(g)** In this field, the proliferation of the junctional component is even more intense, with the formation of nests and a greater number of melanocytes with pagetoid distribution, characteristic of superficial extensive melanomas (H&E 200x). **(h)** In this field, an epidermal nest (teal circle) of atypical melanocytes (yellow arrows) is seen, in correlation with the structures seen on RCM. This magnification allows the observation of a focus of invasion, consisting of melanocytes with the same cytological characteristics of the junctional component: atypia represented by nuclear hyperchromasia, karyomegaly, evident nucleoli, ample cytoplasm (H&E 200x). *Clinical, dermoscopy, and RCM wide-probe mosaic close-up images courtesy of Dra. Ana Maria Sortino. Histopathology images courtesy of Dr. Clovis Antônio Lopes Pinto and Dra. Rafaela Brito de Paula.*

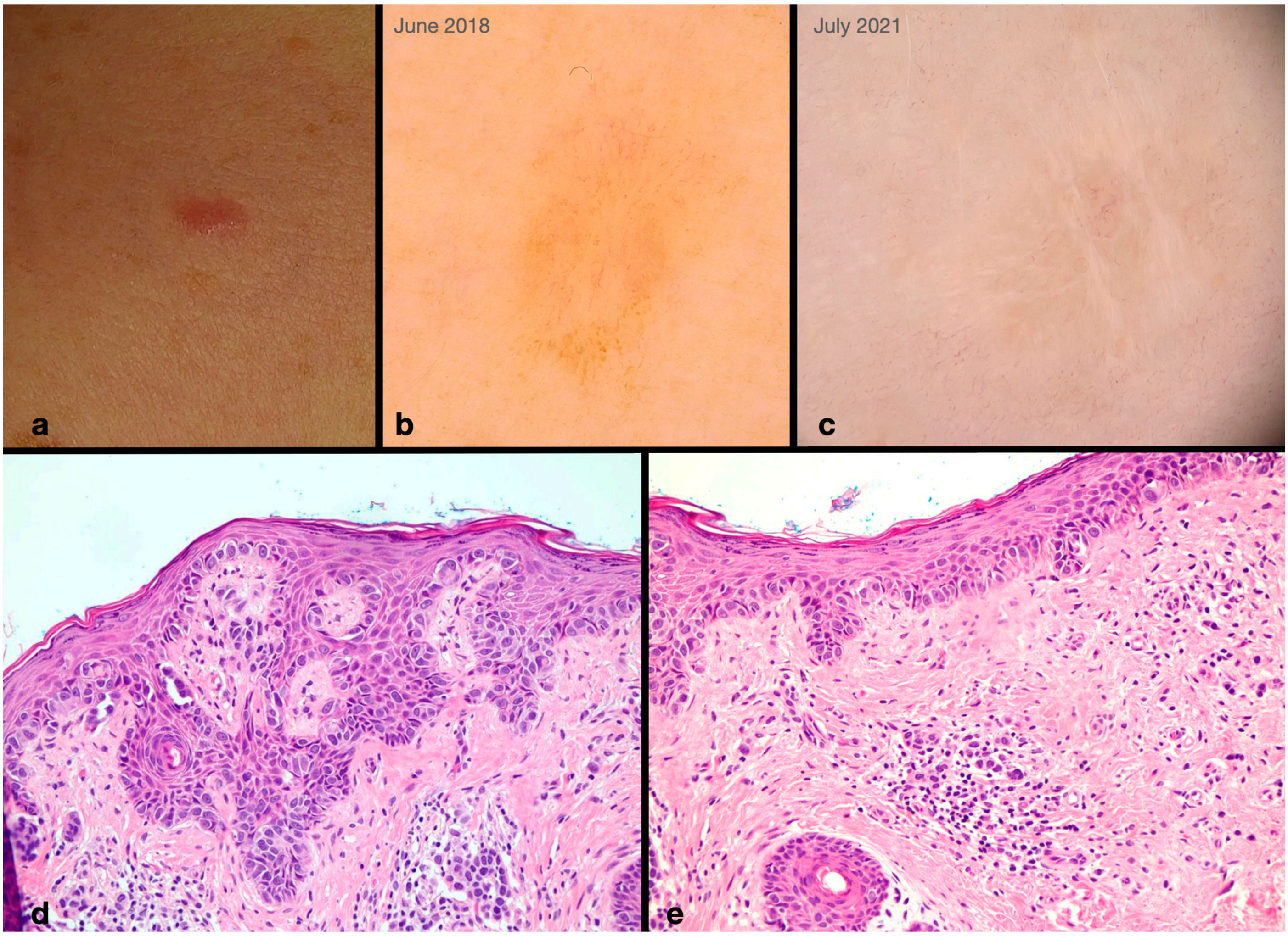


**Figure S22. Female patient: invasive melanoma, superficial spreading type, not associated with nevus ("*de novo*"), Breslow 0.4 mm, mitotic index of 0/10 CGA, with histological inflammatory infiltrate and regression. (a)** Pink lesion on the left ventral trunk. **(b)** Baseline CPD (20x) allows the observation of a tan background with peripheral atypical globules and linear vessels. **(c)** Follow-up CPD (20x) shows an amelanotic lesion with shiny white lines and polymorphous vessels. **(d)** Photomicrograph showing an extensive *in-situ* component of melanoma, with intense cytological atypia, thinning of the epidermis and fusion of the epidermal cones. In the lower right corner, a small focus of invasion in the papillary dermis, associated with a lymphocytic infiltrate (H&E 200x). **(e)** A continuous melanocytic proliferation through the basal layer of the epidermis and prominent nucleoli and presence of a minimal focus of invasion in the papillary dermis, not associated with nevus cells (H&E 200x). *Clinical and dermoscopy images courtesy of Dra. Ana Maria Sortino. Histopathology images courtesy of Dr. Clovis Antônio Lopes Pinto and Dra. Rafaela Brito de Paula.*

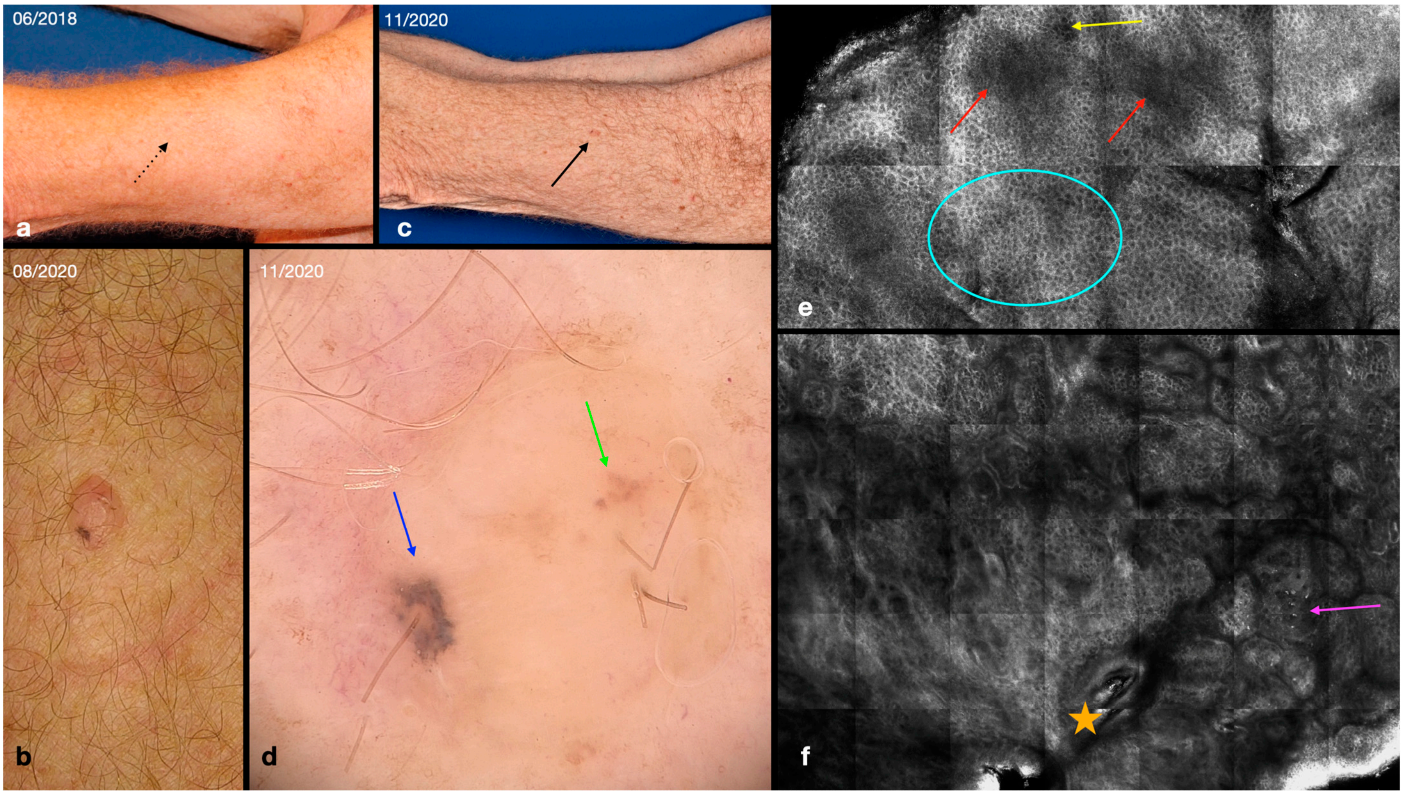


**Figure S23. Male patient: invasive melanoma, superficial spreading type, not associated with nevus ("*de novo*"), Breslow 1.5 mm, mitotic index of 1/10 CGA. (a)** Baseline 2018 TBSP of the left arm, dotted arrow showing the inexistence of the lesion. **(b)** Clinical close-up image of a palpable and firm oval pink lesion, with gray-blue pigmentation around the hair follicle. It was noticed during clinical short-term follow-up in August 2020. Immediate excision was recommended, along with other suspect nevi. However, patient's perception was of a common nevus. He had the other lesions excised but decided not to remove this one. A new follow-up was scheduled for TBSP and RCM. **(c)** TBSP from November 2020 showing growth of an amelanotic/hypopigmented dome-shaped lesion. (**d)** Close-up of CPD (20x) allows the observation of a tan structureless background and perifollicular gray dots and granules (blue arrow). In November the lesion gained irregular brown globules (green arrow) that weren't present at baseline digital dermoscopy from August. **(e)** RCM was read as inconclusive at the examination time. In retrospect, at the epidermal level an atypical honeycomb pattern (teal circle), dark silhouettes (red arrows) and hyporeflective pagetoid cells (yellow arrow) are identified. **(f)** RCM at dermal level, evidencing the hair follicle (orange star), aggregated irregular hyporeflective cells forming nests and, focal bright cells (pink arrow). *Clinical, dermoscopy, and RCM wide-probe mosaic close-up images courtesy of Dra. Ana Maria Sortino.*
